# Nitrosonium Ion-Catalyzed Oxidative Chlorination of Arenes

**DOI:** 10.1021/acs.joc.5c01914

**Published:** 2026-01-05

**Authors:** Ju-Ching Hsu, Yan-Shiun Chen, Chen-Hung Hsiao, Duen-Ren Hou

**Affiliations:** Department of Chemistry, 34911 National Central University, 300 Jhong-Da Road, Jhong-Li, Taoyuan 320317, Taiwan

## Abstract

A nitrosonium-catalyzed chlorination of electron-rich arenes has been developed, employing sodium chloride as the chlorine source, molecular oxygen as the terminal oxidant, and either nitric acid or sulfuric acid as the acid promoter. This transition-metal-free protocol proceeds in acetic acid under mild conditions (25–80 °C), affording chlorinated arenes in good to excellent yields. Substrate scope studies highlighted the roles of hydrogen bonding for methoxy-substituted benzoic acids, and the experimental results indicated the formation of H_2_O_2_ as the byproduct and supported a nitrosonium ion (NO^+^)-catalyzed mechanism. A kinetic isotope effect (KIE) of 1.1 was observed using pentadeuteriophenyl phenyl ether, consistent with the pathway involving a Wheland intermediate.

## Graphical abstract

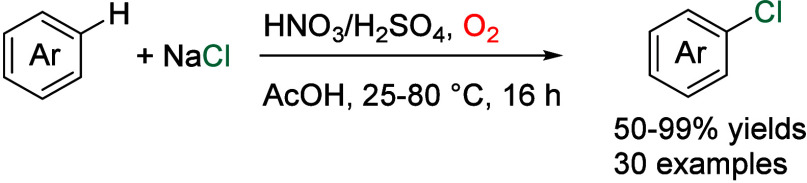

## Introduction

Chlorinated aromatic compounds are valuable synthetic building blocks widely used in cross-coupling,^1^ halogen–metal exchange,^2^ and elimination reactions.^3^ Besides, the incorporation of chloro substituents into aromatic systems significantly alters the electronic and physical properties of molecules, which is crucial for tailoring the performance of pharmaceuticals and functional materials.^4,5^ Given their impact on both reactivity and molecular behavior, chlorination reactions rank among the most frequently employed transformations in the pharmaceutical industry.^6^ Conventional chlorination of arenes involves highly reactive reagents such as Cl_2_ or SO_2_Cl_2_;^7^ however, the hazardous nature of these chemicals makes them impractical for routine laboratory use. Other reagents to give the electrophilic “Cl^+^” source under an ambient condition, including *N*-chlorosuccinimide (NCS) (Scheme 1),^8,9^ 1,3-dichloro-5,5-dimethylhydantoin (DCDMH), and trichloroisocyanuric acid (TCCA),^10^ are commonly employed; however, they generally exhibit reduced chlorination activity, and additional catalysts and/or promoters are often required. Oxidative chlorination is another method to prepare aryl chlorides;^11^ thus, a combination of *p*-toluenesulfonyl chloride (TsCl)/hypervalent iodine reagents,^12^ NCS/2,2,6,6-tetramethylpiperidine nitroxide (TEMPO),^13^ HCl or trimethylsilyl chloride/dimethyl sulfoxide (DMSO),^14^ photocatalyzed^15^ and electrochemical reactions^16^ have been developed for this purpose. Aryl chlorides were also prepared through metal-mediated chlorination reactions.^17^ Chlorination reactions using a single reagent are simple and convenient. Notably, chlorobis­(methoxycarbonyl)­guanidine (CBMG, “Palau’chlor”),^18^ *N*-chloro-*N*-fluorobenzenesulfonylamine (CFBSA),^19^ and 1-chloro-1,2-benziodoxol-3-one^20^ demonstrate high reactivity and enable selective chlorination of arenes. Nevertheless, developing chlorination reactions that utilize readily available reagents and simple procedures and generate minimal waste remains a significant focus in synthetic chemistry. Recently, we employed the nitrosonium ion to catalyze C–Br and C–N bond formation in electron-rich arenes, using oxygen as the terminal oxidant, which significantly reduced the need for additional oxidizing agents.^21^ In 1988, Radner reported moderate yields for the chlorination of naphthalene (58%) and mesitylene (17%) using ammonium chloride (NH_4_Cl), NOBF_4_, and trifluoroacetic acid (TFA) during his study of the iodination of aromatic compounds.^22^ More recently, Bondarenko’s group demonstrated the chlorination of N-heterocycles such as isoxazoles and pyrazoles, employing tetramethylammonium chloride (Me_4_NCl) as the chlorine source and nitrosylsulfuric acid (NOHSO_4_) as the oxidant.^23^ Inspired by these precedents, we investigated a nitrosonium-catalyzed chlorination of arenes using sodium chloride (NaCl) as the chlorine source and molecular oxygen as the oxidant, which simplifies the protocol, reduces chemical waste, and exploits sodium chloride as the most economical and abundant source of chlorine atoms (Scheme 1).^24^

**Scheme 1:**
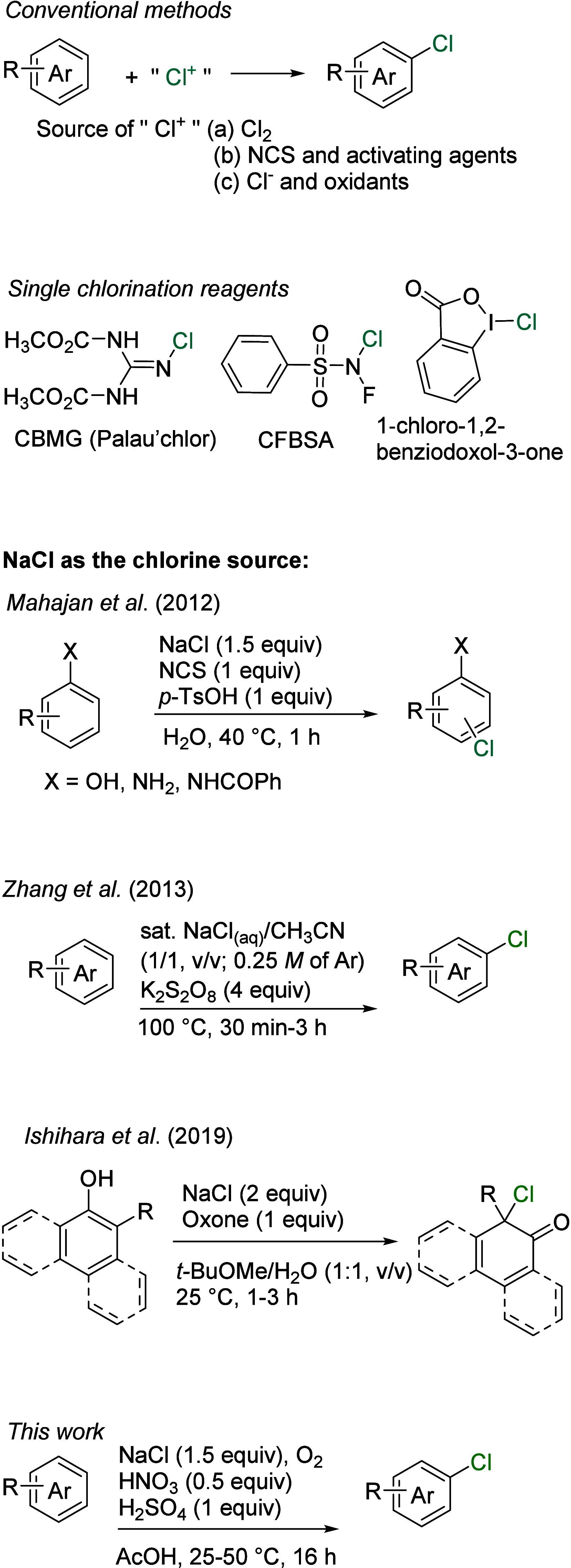
Approaches to Prepare Aryl Chlorides

## Results and Discussion

We found that adding a catalytic amount (50 mol %) of nitric acid (ACS reagent, 70%) to a mixture of anisole (**1a**), sodium chloride (1.5 equiv), and sulfuric acid in glacial acetic acid, under an oxygen atmosphere, followed by stirring at 50 °C for 16 h, produced 4- and 2-chloroanisole (**2a**) in a combined yield of 99% (entry 1, Table 1). The reaction workup was straightforward. Dilution with water, extraction with ethyl acetate, drying, and concentration directly afforded the product. The remaining waste after neutralization was the aqueous acetic acid solution of sodium chloride, nitrate, and sulfate salts, highlighting the efficiency and minimal environmental impact of this protocol. A gram-scale (10 mmol) reaction afforded *p*-**2a** and *o*-**2a** in 65% and 27% yields, respectively (Experimental Section). Without oxygen, i.e., under a nitrogen atmosphere, the reaction was sluggish with 39% of starting **1a** recovered (entry 2). Nitric acid was essential, as no reaction occurred without it (entry 3). On the other hand, the reaction in which the amount of nitric acid increased while that of sulfuric acid decreased, thus maintaining a constant proton concentration, yielded a significant amount of nitroanisole (entry 4). Conducting the reaction at room temperature slightly improved the *para*:*ortho* ratio of product **2a** despite the lower conversion (entry 5). The reaction did not reach completion with less sulfuric acid (entry 6), but the nitration reaction became prevalent when more sulfuric acid (2.0 mmol) was applied (entry 7). Adding water (60 μL) to obtain a homogeneous solution did not improve the chlorination reaction (entry 8). All other reagents leading to the nitrosonium ion, including NaNO_2_, NaNO_3_, NOBF_4_, NO_2_BF_4_, and *tert*-butyl nitrite, could be applied to catalyze this reaction, although they were less effective than HNO_3_ (entries 9–13, respectively). Both TFA and hydrogen chloride could also be used (entries 14 and 15, respectively); however, reactant **1a** was retrieved, compared with the reaction using sulfuric acid. Interestingly, no chlorination was observed when using TFA and NaNO_3_, the same reagents and reaction condition we developed for the bromination of arenes except that NaBr was replaced with NaCl and a higher reaction temperature (25 °C vs 50 °C) was used (entries 16 and 17).^21b^

**Table 1:**
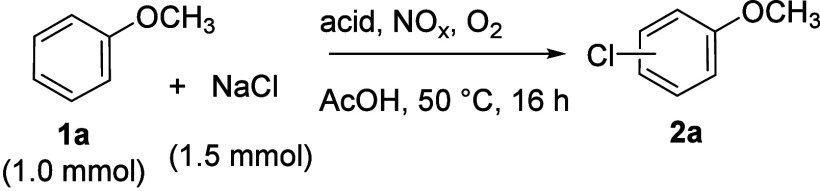
Oxidative Chlorination of Anisole^a^

entry	acid (mmol)	source of NO_*x*_ (mmol)	yields of *p*-**2a**, *o*-**2a**, recovered **1a** (%)
1	H_2_SO_4_ (1.0)	HNO_3_ (0.50)	72, 27, 0
2	H_2_SO_4_ (1.0)	HNO_3_ (0.50)	42, 17, 39^b^
3	H_2_SO_4_ (1.0)	–	0, 0, 99
4	–	HNO_3_ (2.5)	58, 20, 0^c^
5^d^	H_2_SO_4_ (1.0)	HNO_3_ (0.50)	68, 19, 12
6	H_2_SO_4_ (0.75)	HNO_3_ (0.50)	65, 22, 10
7^d^	H_2_SO_4_ (2.0)	HNO_3_ (0.50)	25, 17, 20^e^
8^f^	H_2_SO_4_ (1.0)	HNO_3_ (0.50)	46, 24, 24
9	H_2_SO_4_ (1.0)	NaNO_2_ (0.50)	58, 21, 20
10	H_2_SO_4_ (1.0)	NaNO_3_ (0.50)	20, 16, 43^g^
11	H_2_SO_4_ (1.0)	NOBF_4_ (0.50)	55, 25, 17
12	H_2_SO_4_ (1.0)	NO_2_BF_4_ (0.50)	50, 28, 19
13	H_2_SO_4_ (1.0)	*t*-BuONO (0.50)	63, 18, 16
14	TFA (2.0)	HNO_3_ (0.50)	53, 25, 19
15	HCl^h^ (2.0)	HNO_3_ (0.50)	51, 17, 31
16	TFA (2.2)	NaNO_3_ (0.50)	0, 0, 50^i^
17^d^	TFA (2.2)	NaNO_3_ (0.50)	0, 0, 100

a NO_*x*_ was added to a mixture of **1a** (108.1 mg, 1.0 mmol), NaCl (87.7 mg, 1.5 mmol), an acid, and glacial acetic acid (1.5 mL), and the reaction mixture was stirred at 50 °C under an oxygen atmosphere (balloon) for 16 h.

b The reaction was conducted under a nitrogen atmosphere.

c With *o*- and *p*-nitroanisole (20%).

d Reaction temperature of 25 °C.

e With *o*- and *p*-nitroanisole (37%).

f Water (60 μL) was added before the addition of HNO_3_.

g With *o*- and *p*-nitroanisole (15%).

h Anhydrous HCl_(g)_ in acetic acid.

i With *o*- and *p*-nitroanisole (49%).

The reaction conditions, sodium chloride with HNO_3_/H_2_SO_4_ under an O_2_ atmosphere at 50 °C (Table 1, entry 1), were applied to synthesize a variety of chlorinated arenes (Table 2). Electron-rich arenes, including diphenyl ether, 2-isopropyl-anisole, 2,6-dimethylanisole, and 1- and 2-methoxynaphthalene, afforded the corresponding chlorinated products (**2b–2f**, respectively) in good to excellent yields (75–95%) under these standard conditions. For substrates bearing multiple methoxy groups, such as 1,4-dimethoxybenzene and 1,2,3-trimethoxybenzene, the reactions were conducted at room temperature, yielding products **2g** and **2h**, respectively; notably, **2h** was obtained in 99% yield with a regioisomeric ratio of 3.5. However, when the same conditions were applied to 1,2- and 1,3-dimethoxybenzene, nitration products 1,2-dimethoxy-4-nitrobenzene and 2,4-dimethoxy-1-nitrobenzene, respectively, were obtained instead, each in 0.5 equiv (99% yield, based on nitric acid as the limiting reagent), along with the recovery of 0.5 equiv of the starting material (see Table S1). In fact, 1,4-dimethoxybenzene (**1g**) has the lowest oxidation potential (1.28 V vs SCE) among the three dimethoxybenzenes, although the oxidation potentials of 1,2- and 1,3-dimethoxybenzene (1.42 and 1.55 V, respectively) are still lower than that of anisole (1.77 V).^25^ These results indicate that arene chlorination is influenced by not only the oxidation potentials of the substrates but also the competing nitration reaction, which is likewise initiated by NO^+^ in acetic acid.^26^ Toluene remained inert under these conditions, as did benzene, fluorobenzene, and substrates that are readily protonated under acidic conditions (aniline, imidazole, and phenol (see Table S1)). This lack of reactivity is attributed to their high oxidation potentials and is consistent with our previous observations of the bromination reaction.^21b,27^ In contrast, chlorination of xylenes proceeded with 2 equiv of nitric acid, giving products **2i–2k** in moderate yields (60–63%). For *o*-xylene, both 3- and 4-chloro regioisomers were formed in a 1.2:1 ratio. Reactions with 1,3,5-, 1,2,4-, and 1,2,3-trimethylbenzenes gave slightly improved yields of **2l–2n** (65–76%), respectively, although dichlorinated product **2m′** was also observed from 1,2,4-trimethylbenzene. Using the reagents *tert*-butyl nitrite and hydrogen chloride in acetic acid significantly reduced the level of formation of **2m′** (footnote [Table-fn t2fn4] of Table 2). Additionally, 1,2,4,5-tetramethylbenzene afforded **2o** in good yield (90%), and naphthalene was selectively chlorinated to give **2p** in 67% yield. We noticed that the reaction of naphthalene was sensitive to the amount of nitric acid, and 1-nitronaphthalene was formed as the dominant product when >2 equiv of HNO_3_ was used.

**Table 2:**
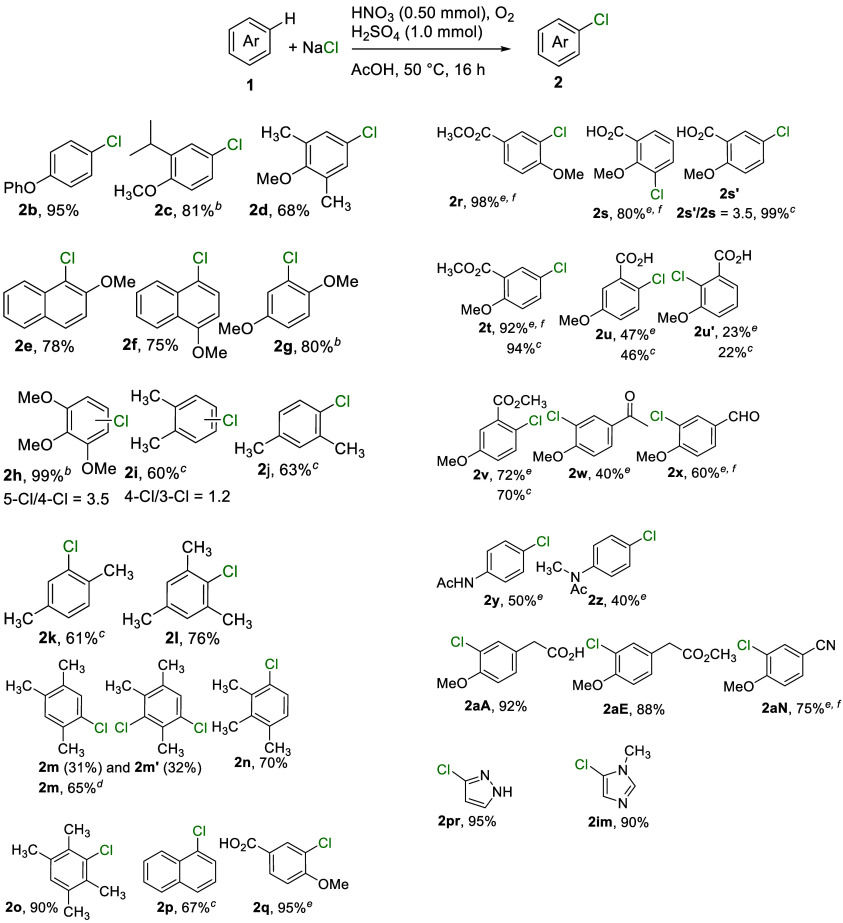
Preparation of Chlorinated Arenes^a^

a Nitric acid (45.0 mg, 0.50 mmol) was added to a mixture of arene **1** (1.0 mmol), NaCl (87.7 mg, 1.5 mmol), sulfuric acid (98.1 mg, 1.0 mmol), and glacial acetic acid (1.5 mL), and the reaction mixture was stirred at 50 °C under an oxygen atmosphere (balloon) for 16 h.

b Reaction conducted at 25 °C.

c Nitric acid (180.0 mg, 2.0 mmol) was added to a mixture of arene **1** (1.0 mmol), NaCl (87.7 mg, 1.5 mmol), and glacial acetic acid (1.5 mL), and the reaction mixture was stirred at 50 °C under an oxygen atmosphere (balloon) for 16 h.

d *tert*-Butyl nitrite (103.1 mg, 1.0 mmol) was added to a mixture of arene **1** (1.0 mmol) and HCl_(g)_ in glacial acetic acid (6.3 wt %, 1.9 mL, 3.6 mmol), and the reaction mixture was stirred at 50 °C under an oxygen atmosphere (balloon) for 16 h.

e Same as condition a with HNO_3_ (90.0 mg, 1.0 mmol) and H_2_SO_4_ (147.1 mg, 1.5 mmol).

f Reaction conducted at 80 °C.

The reactions of *p*-methoxy-benzoic acid and its methyl ester gave expected compounds **2q** and **2r** in 95% and 98% yields, respectively. In contrast, the corresponding reaction of the *o*-methoxy-benzoic acid was intriguing. Under the modified standard conditions (footnotes [Table-fn t2fn5] and [Table-fn t2fn6] of Table 2), only 3-Cl-**2s** was formed, whereas 5-Cl-**2s′** became the dominant product in the absence of sulfuric acid (footnote [Table-fn t2fn3] of Table 2). This outcome can be attributed to the differing abilities of the conjugate bases, sulfate and nitrate, to perturb the intramolecular hydrogen-bonding interactions between the carboxylic acid and the *o*-methoxy group.^28^ Accordingly, both conditions yielded a single product, methyl 5-chloro-2-methoxybenzoate (**2t**), from methyl *o*-methoxybenzoate (**1t**), in which intramolecular hydrogen bonding is absent. The intramolecular hydrogen bonding in *m*-methoxybenzoic acid (**1u**) should also be minimized,^28^ resulting in similar chlorination outcomes regardless of the acid used. However, the formation of two products (**2u** and **2u′**) from the chlorination of **1u**, compared to the exclusive formation of methyl 2-chloro-5-methoxybenzoate (**2v**) from its methyl ester **1v**, suggests the presence of intermolecular hydrogen bonding between the carboxylic acid and the reaction medium.^29^ Therefore, hydrogen bonding complements the *ortho*-metalation strategy in the chlorination of methoxy-substituted benzoic acids.^30^

Anisoles bearing *para*-electron-withdrawing groups, such as acetyl and formyl moieties, afforded products **2w** and **2x** in 40% and 60% yields, respectively. Although aniline was inactive under the acidic conditions due to the protonation under acidic conditions (Table S1), *N*-phenylacetamide and its N-methylated derivatives underwent chlorination successfully, affording **2y** and **2z**, respectively, in moderate yields. It was interesting to note that *o*-, *m*-, and *p*-methylanisoles did not undergo chlorination but nitration (Table S1); however, the functionalized *p*-methylanisoles participated in the reaction smoothly, providing products **2aA** and **2aE** in good yields. 3-Chloro-4-methoxybenzonitrile (**2aN**) was prepared in 75% yield under the same conditions employed for the formation of formyl-substituted **2x**. Nitrogen hetrocycles, such as pyrazole and *N*-methylimidazole, were also suitable substrates for this chlorination, affording chloro-substituted **2pr** (95%) and **2im** (90%), respectively.

This chlorination was used to prepare pharmaceutical intermediates. For example, 4-amino-5-chloro-2-methoxybenzoic acid (**3**)—a precursor to metoclopramide, a dopamine D_2_ receptor antagonist used to treat nausea, vomiting, and gastroparesis^31^—was synthesized from carbamate **4** through a sequence of chlorination and hydrogenolysis (Scheme 2). In addition, oxazepam, a benzodiazepine used to treat anxiety,^32^ was formally prepared via the regioselective, late-stage chlorination of amide **5**, rather than using 4-chloroaniline as the starting material in the current protocol (Scheme 3).^32,33^

**Scheme 2:**
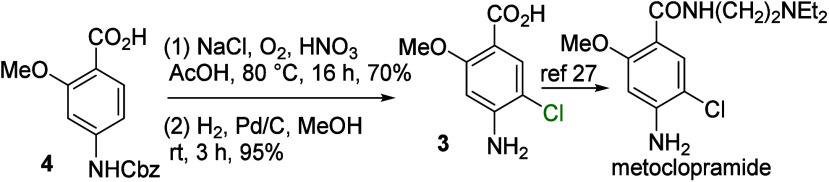
Formal Synthesis of Metoclopramide

**Scheme 3:**
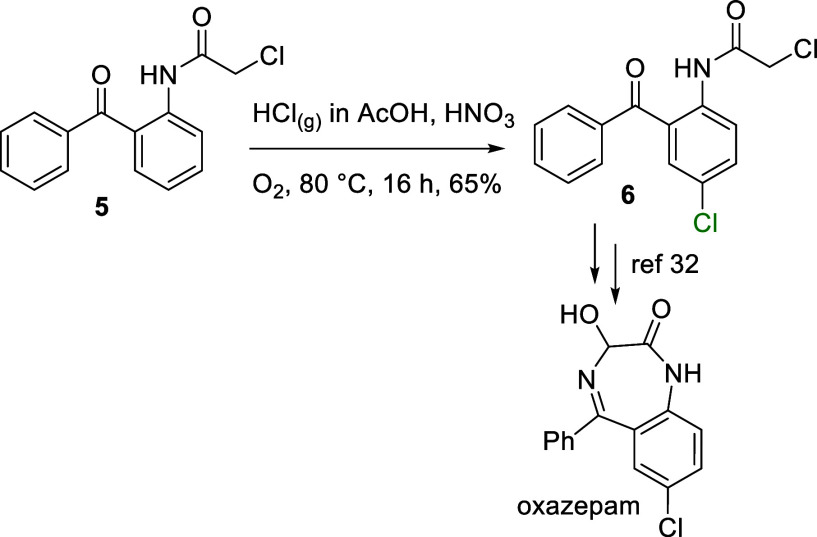
Late-Stage Chlorination and Formal Synthesis of Oxazepam

We found that this chlorination was inhibited by 20 mol % sodium azide, a NO^+^ scavenger,^34^ and *trans*-stilbene (eqs 1 and 2).
1

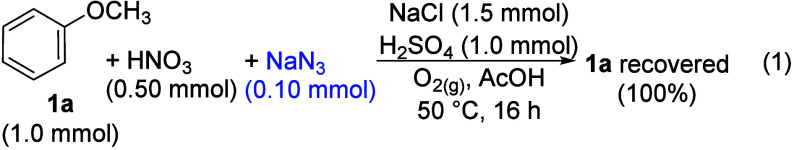



2

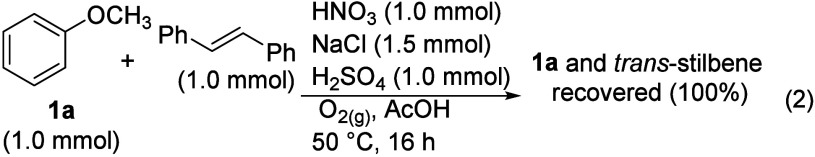

The lower oxidation potential of stilbene, compared to that of anisole (**1a**),^35^ and the inertness of the resulting cationic stilbene radical toward the chloride ion may explain the inhibitory effect of stilbene. Hydrogen peroxide (H_2_O_2_) was detected in the conversion of **1g** into **2g** by using a starch-iodide test. The concentration of H_2_O_2_ in the aqueous phase after extraction was determined to be 12.1 mM by the FOX assay,^36^ corresponding to a 12% yield (see the Supporting Information). The low yield of H_2_O_2_ is likely due to the prolonged reaction time (16 h) and the strongly acidic conditions, which are known to promote the decomposition of H_2_O_2_ (eq 3).^37^

3

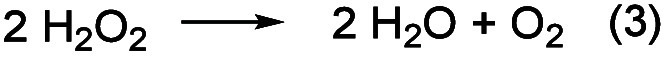

Nevertheless, the formation of H_2_O_2_ as a byproduct in the chlorination reaction is well-supported. In contrast, the corresponding reaction conducted without NaCl afforded 1,4-dimethoxy-2-nitrobenzene (0.48 mmol, 96% yield, eq 4), and no H_2_O_2_ was detected (Figure S1), indicating that the presence of chloride suppressed the nitration and facilitated the chlorination pathway and H_2_O_2_ was a practical indicator for distinguishing between nitration and chlorination pathways.
4

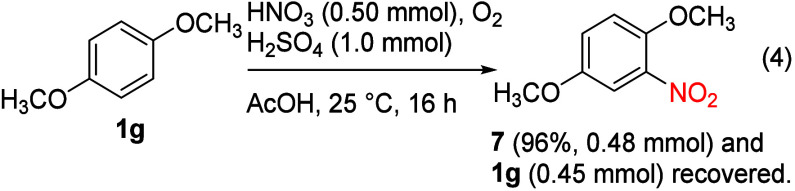

 In this case, nitration was stoichiometrically dependent on nitric acid, but chlorination of **1g** is a catalytic process (Table 2). The coexistence of NO_2_^+^ and NO^+^ in HNO_3_ is well established,^38^ with NO^+^ being recognized as an efficient single-electron acceptor.^39^ These results suggested the reaction mechanism shown in eqs 5–8 and Scheme 4.
5

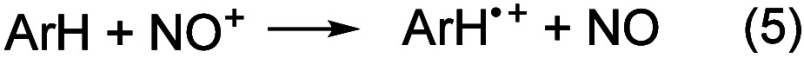



6

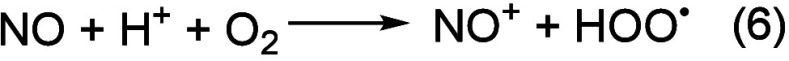



7

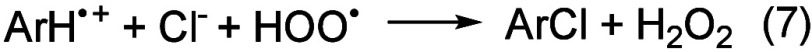



8

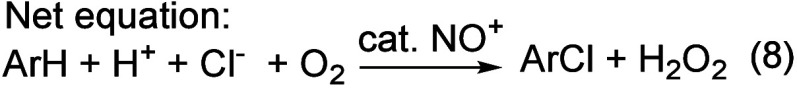




**Scheme 4:**
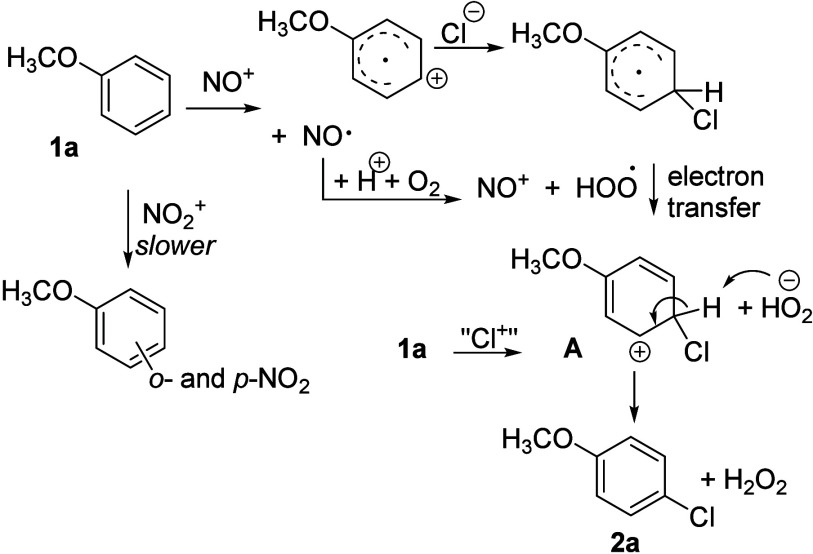
Two Routes to Intermediate **A** and Formation of Product **2a**

To investigate the kinetic isotope effect (KIE) in this chlorination reaction, a competitive experiment was carried out using pentadeuteriophenyl phenyl ether (*d*_5_-**1b**). The results were analyzed by comparing the ^13^C­{^1^H} NMR spectra of **1b**, *d*_5_ **-1b**, **2b**, and the products of chlorination using *d*_5_ **-1b** (panels a–d, respectively, of Figure 1). Panels a and b of Figure 1 show that the absorptions of the phenyl groups in **1b** and the C_6_H_5_ group in *d*_5_-**1b** were indistinguishable, and the integrations of C_*o*_, C_*m*_, and C_*p*_ were consistent with their respective carbon counts. By comparing the ^13^C NMR spectra of **1b** and its chlorinated product **2b** (Figure 1a,c), the signals corresponding to the phenyl and *p*-chlorophenyl groups in **2b** were assigned as C_*o′*_, C_*m′*_, C_*p′*_ and C_2_, C_3_, and C_4_, respectively. These same assignments were applied to the chlorination product mixture (Figure 1d), which contained two products, *d*_5_-**2b** and *d*_4_-**2b**, along with unreacted *d*_5_-**1b**. The ratio of *d*_5_-**2b** to *d*_4_-**2b** (i.e., *k*_H_/*k*_D_) was determined to be 1.1, based on the integrations of C_2′_ and twice that of C_*p″*_, whose signals were well resolved and free from overlapping. This kinetic isotope effect is consistent with reported values of 1.2 and 1.1 for the chlorination of anisole using Palau’chlor and *N*-chlorosuccinimide (NCS), respectively.^9b,18^ The small KIE suggests that C–H bond cleavage from Wheland intermediate **A** to **2a** (Scheme 4) is not rate-determining. While typical chlorination uses electrophilic “Cl^+^” to form **A** directly, this reaction proceeds via sequential one-electron transfers from anisole to nitrosonium and then to the hydroperoxyl radical, to give the same intermediate **A**.

**Figure 1:**
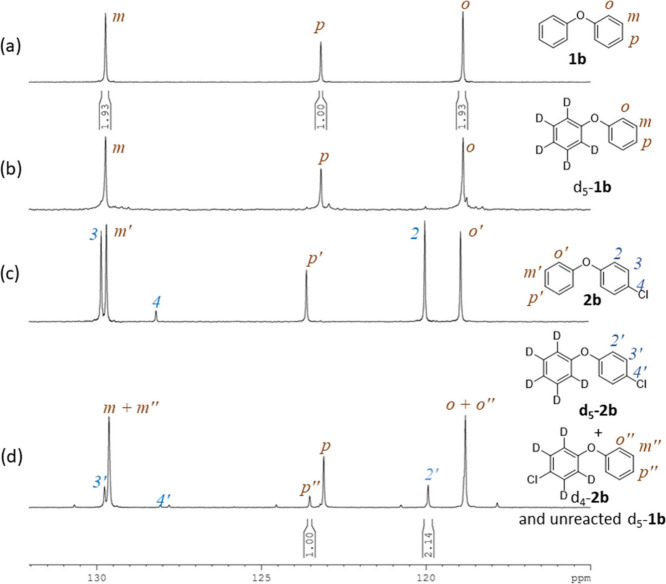
^13^C­{^1^H} NMR spectra (126 MHz, CDCl_3_) of (a) **1b**, (b) *d*_5_ - **1b**, (c) **2b**, and (d) chlorination reaction products derived from *d*_5_ - **1d**.

## Conclusion

In summary, we have developed a convenient and environmentally friendly chlorination method for arenes. Unlike bromination, this reaction employs nitric acid as a more effective NO^+^ source, with sulfuric acid preferred over persistent trifluoroacetic acid.^40^ Readily available sodium chloride serves as the chlorine source, while molecular oxygen acts as the terminal oxidant, regenerating nitrosonium (NO^+^) from nitric oxide (NO) and producing hydrogen peroxide as the byproduct. The formation of H_2_O_2_ provides a useful marker to distinguish between the nitration and chlorination pathways. The effects of hydrogen bonding were also observed in the chlorinations of methoxy-substituted benzoic acids. The observed KIE, comparable to that of electrophilic Cl^+^ reactions, supports a mechanism involving a Wheland intermediate. The broad substrate scope of this method enables late-stage chlorination, as demonstrated in the syntheses of two pharmaceutical precursors. Importantly, this method avoids the use of hazardous Cl_2_ or Cl^+^ reagents, which enhances operational safety. The exclusion of transition metals and organic catalysts significantly simplifies waste management and improves the overall sustainability of the process.

## Experimental Section

### General Information

Reagents, such as NaCl, NaNO_3_, NaNO_2_, NOBF_4_, NO_2_BF_4_, *tert*-butyl nitrite, TFA, sulfuric acid, nitric acid, and acetic acid, were purchased from commercial sources (ACS grade) and used without further purification. Thin-layer chromatography (TLC) was conducted using precoated silica gel 60 F254 plates containing a fluorescent indicator. Spots were examined under UV light or revealed by a KMnO_4_ solution. Purification by chromatography was conducted using silica gel (230–400 mesh). The ^1^H and ^13^C NMR spectra were recorded in a CDCl_3_ solution using Bruker Avance 500 and 300 NMR spectrometers. Chemical shifts for ^1^H and ^13^C­{^1^H} NMR spectra are reported in δ units (parts per million) with reference to residual solvent peaks. High-resolution mass spectrometry (HRMS) data were recorded on a JMS-700 quadrupole mass spectrometer. Compound *d*_5_ - **1b** was prepared according to the procedure outlined in the literature.^41^ The concentration of H_2_O_2_ was determined by measuring the amount of FOX reagent based on its absorbance at 560 nm using an Epoch 2 microplate spectrophotometer.

#### 1-Chloro-4-methoxybenzene (*p*-**2a**) and 1-Chloro-2-methoxybenzene (*o*-**2a**)

Nitric acid (70%, 45.0 mg, 0.50 mmol) was added to a mixture of anisole (**1a**, 108.1 mg, 1.0 mmol), sodium chloride (87.7 mg, 1.5 mmol), sulfuric acid (98.1 mg, 1.0 mmol), and glacial acetic acid (1.5 mL). The reaction mixture was stirred at 50 °C (oil bath) under an oxygen atmosphere (balloon) for 16 h, water (1.5 mL) added, and the mixture extracted with ethyl acetate (3 × 5 mL). The combined organic layers were dried with sodium sulfate, filtered, and concentrated to give a mixture of chloroanisoles *p*-**2a** and *o*-**2a** (2.7:1, 141.2 mg, 0.99 mmol, 99%) as a colorless liquid. *p*-**2a**: ^1^H NMR (300 MHz, CDCl_3_) δ 7.23 (d, *J* = 9.0 Hz, 2H), 6.82 (d, *J* = 9.0 Hz, 2H), 3.76 (s, 3H); ^13^C­{^1^H} NMR (75 MHz, CDCl_3_) δ 158.1, 129.1, 125.3, 115.0, 55.2. *o*-**2a**: ^1^H NMR (300 MHz, CDCl_3_) δ 7.37 (dd, *J* = 8.0, 1.5 Hz, 1H), 7.20 (d, *J* = 1.5 Hz, 1H), 6.91 (d, *J* = 8.0 Hz, 1H), 6.87 (d, *J* = 1.5 Hz, 1H), 3.87 (s, 3H); ^13^C­{^1^H} NMR (75 MHz, CDCl_3_) δ 154.9, 130.1, 127.6, 125.7, 121.1, 111.9, 55.8. The spectroscopic data were consistent with the reported values.^42^ For the gram-scale synthesis of **2a**, nitric acid (0.45 g, 5.0 mmol) was added to a mixture of anisole (**1a**, 1.20 g, 10.0 mmol), sodium chloride (0.88 g, 15.0 mmol), sulfuric acid (0.98 g, 10.0 mmol), and acetic acid (15 mL) in a 100 mL round-bottom flask. The reaction mixture was stirred at 50 °C (oil bath) under an oxygen atmosphere for 16 h, water (20 mL) added, and the mixture extracted with ethyl acetate (3 × 20 mL). The combined organic layers were dried with sodium sulfate, filtered, and concentrated to give *p*-**2a** and *o*-**2a** (2.4:1, 1.32 g, 9.2 mmol, 92%).

#### 1-Chloro-4-phenoxybenzene (**2b**)

The procedure to prepare **2a** was followed. Starting with NaCl (87.7 mg, 1.5 mmol), acetic acid (1.5 mL), diphenyl ether (**1b**, 170.2 mg, 1.0 mmol), sulfuric acid (98.1 mg, 1.0 mmol), and HNO_3_ (45.0 mg, 0.50 mmol), product **2b** (194.4 mg, 0.95 mmol, 95%) was harvested after column chromatography (SiO_2_, hexanes, *R*_*f*_ = 0.8) as a colorless liquid: ^1^H NMR (500 MHz, CDCl_3_) δ 7.39 (t, *J* = 8.0 Hz, 2H), 7.33 (d, *J* = 8.0 Hz, 2H), 7.18 (t, *J* = 7.4 Hz, 1H), 7.06 (d, *J* = 7.4 Hz, 2H), 6.99 (d, *J* = 7.4 Hz, 2H); ^13^C­{^1^H} NMR (126 MHz, CDCl_3_) δ 156.8, 155.9, 129.8, 129.6, 128.1, 123.6, 120.0, 118.9. The spectroscopic data were consistent with the reported values.^43^

#### 4-Chloro-2-isopropylanisole (**2c**)

Nitric acid (70%, 45.0 mg, 0.50 mmol) was added to a mixture of 2-isopropylanisole (**1c**, 150.2 mg, 1.0 mmol), sodium chloride (87.7 mg, 1.5 mmol), sulfuric acid (98.1 mg, 1.0 mmol), and glacial acetic acid (1.5 mL). The reaction mixture was stirred at 25 °C under an oxygen atmosphere (balloon) for 16 h, water (1.5 mL) added, and the mixture extracted with ethyl acetate (3 × 5 mL). The combined organic layers were dried with sodium sulfate, filtered, and concentrated. The crude product was purified by column chromatography (SiO_2_, 1:9 EtOAc/hexanes, *R*_*f*_ = 0.8) to give **2c** (149.2 mg, 0.81 mmol, 81%) as a light yellow liquid: ^1^H NMR (300 MHz, CDCl_3_) δ 7.16 (d, *J* = 2.7 Hz, 1H), 7.11 (dd, *J* = 8.4, 2.7 Hz, 1H), 6.76 (d, *J* = 8.4 Hz, 1H), 3.81 (s, 1H), 3.29 (sept, *J* = 7.3 Hz, 1H), 1.2 (d, *J* = 7.3 Hz, 6H); ^13^C­{^1^H} NMR (75 MHz, CDCl_3_) δ 155.3, 138.9, 127.4, 126.2, 126.1, 125.5, 111.4, 55.6, 26.7, 22.4; HRMS (EI) *m*/*z* [M]^+^ calcd for C_10_H_13_ClO 184.0655, found 184.0655.

#### 4-Chloro-2,6-dimethylanisole (**2d**)

The procedure to prepare **2a** was followed. Starting with NaCl (87.7 mg, 1.5 mmol), acetic acid (1.5 mL), 2,6-dimethylanisole (**1d**, 136.2 mg, 1.0 mmol), sulfuric acid (98.1 mg, 1.0 mmol), and HNO_3_ (45.0 mg, 0.50 mmol), product **2d** (116.7 mg, 0.68 mmol, 68%) was harvested after column chromatography (SiO_2_, 1:9 EtOAc/hexanes, *R*_*f*_ = 0.8) as a light yellow liquid: ^1^H NMR (300 MHz, CDCl_3_) δ 7.00 (s, 2H), 3.71 (s, 3H), 2.27 (s, 6H); ^13^C­{^1^H} NMR (75 MHz, CDCl_3_) δ 155.6, 132.6, 128.8, 128.4, 59.8, 16.0. It was difficult to spearate **2d** from the starting material; however, the spectroscopic data were consistent with the reported values.^44^

#### 1-Chloro-2-methoxynaphthalene (**2e**)

The procedure to prepare **2a** was followed. Starting with NaCl (87.7 mg, 1.5 mmol), acetic acid (1.5 mL), 2-methoxynaphthalene (**1e**, 158.2 mg, 1.0 mmol), sulfuric acid (98.1 mg, 1.0 mmol), and HNO_3_ (45.0 mg, 0.50 mmol), product **2e** (151.1 mg, 0.78 mmol, 78%) was harvested after column chromatography (SiO_2_, 1:9 EtOAc/hexanes, *R*_*f*_ = 0.5) as a colorless solid: mp 60.0–62.0 °C; ^1^H NMR (300 MHz, CDCl_3_) δ 8.24 (d, *J* = 9.2 Hz, 1H), 7.78 (t, *J* = 9.2 Hz, 2H), 7.61–7.55 (m, 1H), 7.44–7.38 (m, 1H), 7.28 (d, *J* = 9.0 Hz, 1H), 4.02 (s, 3H); ^13^C­{^1^H} NMR (75 MHz, CDCl_3_) δ 152.4, 131.8, 129.4, 127.9, 127.4, 124.2, 123.3, 116.6, 113.5, 56.8. The spectroscopic data were consistent with the reported values.^14c^

#### 1-Chloro-4-methoxynaphthalene (**2f**)

The procedure to prepare **2a** was followed. Starting with NaCl (87.7 mg, 1.5 mmol), acetic acid (3.0 mL), 1-methoxynaphthalene (**1f**, 158.2 mg, 1.0 mmol), sulfuric acid (98.1 mg, 1.0 mmol), and HNO_3_ (45.0 mg, 0.50 mmol), product **2f** (144.5 mg, 0.75 mmol, 75%) was harvested after column chromatography (SiO_2_, 1:9 EtOAc/hexanes, *R*_*f*_ = 0.5) as a colorless liquid: ^1^H NMR (300 MHz, CDCl_3_) δ 8.29 (d, *J* = 7.8 Hz, 1H), 8.20 (d, *J* = 7.8 Hz, 1H), 7.65–7.59 (m, 1H), 7.57–7.53 (m, 1H), 7.46 (d, *J* = 8.1 Hz, 1H), 6.73 (d, *J* = 8.1 Hz, 1H), 4.00 (s, 3H); ^13^C­{^1^H} NMR (75 MHz, CDCl_3_) δ 154.5, 131.2, 127.4, 126.3, 125.8, 125.7, 124.1, 123.1, 122.3, 103.7, 55.5. The spectroscopic data were consistent with the reported values.^12c^

#### 2-Chloro-1,4-dimethoxybenzene (**2g**)

Nitric acid (70%, 45.0 mg, 0.50 mmol) was added to a mixture of 1,4-dimethoxybenzene (**1g**, 138.2 mg, 1.0 mmol), sodium chloride (87.7 mg, 1.5 mmol), sulfuric acid (98.1 mg, 1.0 mmol), and glacial acetic acid (1.5 mL). The reaction mixture was stirred at 25 °C under an oxygen atmosphere (balloon) for 16 h, water (3 mL) added, and the mixture extracted with ethyl acetate (3 × 5 mL). The combined organic layers were dried over sodium sulfate, filtered, and concentrated. The crude product was purified by column chromatography (SiO_2_, 1:9 EtOAc/hexanes, *R*_*f*_ = 0.6) to give **2g** (137.4 mg, 0.80 mmol, 80%) as a colorless liquid: ^1^H NMR (300 MHz, CDCl_3_) δ 6.95 (d, *J* = 3.0 Hz, 1H), 6.86 (d, *J* = 9.0 Hz, 1H), 6.76 (dd, *J* = 9.0, 3.0 Hz, 1H), 3.85 (s, 3H), 3.76 (s, 3H); ^13^C­{^1^H} NMR (75 MHz, CDCl_3_) δ 153.7, 149.3, 116.1, 114.6, 113.1, 112.8, 56.7, 55.8. The spectroscopic data were consistent with the reported values.^14c^

#### 5-Chloro-1,2,3-trimethoxybenzene (**2h**)

Nitric acid (70%, 45.0 mg, 0.50 mmol) was added to a mixture of 1,2,3-trimethoxybenzene (**1h**, 168.2 mg, 1.0 mmol), sodium chloride (87.7 mg, 1.5 mmol), sulfuric acid (98.1 mg, 1.0 mmol), and glacial acetic acid (1.5 mL). The reaction mixture was stirred at 25 °C under an oxygen atmosphere (balloon) for 16 h, water (3 mL) added, and the mixture extracted with ethyl acetate (3 × 5 mL). The combined organic layers were dried over sodium sulfate, filtered, and concentrated. The crude product was purified by column chromatography (SiO_2_, 1:9 EtOAc/hexanes) to give **2h** (156.1 mg, 0.77 mmol, 77%) and 4-chloro-1,2,3-trimethoxybenzene (**2h′**, 45.1 mg, 0.22 mmol, 22%). **2h** was a light yellow solid: *R*_*f*_ = 0.44; mp 58.0–60.0 °C; ^1^H NMR (300 MHz, CDCl_3_) δ 6.55 (s, 2H), 3.82 (s, 6H), 3.79 (s, 3H); ^13^C­{^1^H} NMR (75 MHz, CDCl_3_) δ 153.5, 136.6, 128.8, 105.8, 60.8, 56.1. The spectroscopic data were consistent with the reported values.^45^ **2h′** was a colorless liquid: *R*_*f*_ = 0.36; ^1^H NMR (300 MHz, CDCl_3_) δ 7.03 (d, *J* = 9.0 Hz, 1H), 6.60 (d, *J* = 9.0 Hz, 1H), 3.90 (s, 3H), 3.87 (s, 3H), 3.83 (s, 3H); ^13^C­{^1^H} NMR (75 MHz, CDCl_3_) δ 152.6, 149.9, 143.5, 123.8, 119.8, 107.7, 61.1, 56.1. The spectroscopic data were consistent with the reported values.^12c^

#### 4-Chloro-1,2-dimethylbenzene (**2i**)

Nitric acid (70%, 180.0 mg, 2.0 mmol) was added to a mixture of *o*-xylene (**1i**, 106.2 mg, 1.0 mmol), sodium chloride (87.7 mg, 1.5 mmol), and glacial acetic acid (1.5 mL). The reaction mixture was stirred at 50 °C under an oxygen atmosphere (balloon) for 16 h, water (3 mL) added, and the mixture extracted with ethyl acetate (3 × 5 mL). The combined organic layers were dried with sodium sulfate, filtered, and concentrated. The crude product was purified by column chromatography (SiO_2_, 1:3 EtOAc/hexanes, *R*_*f*_ = 0.8) to give a mixture of **2i** and 3-chloro-1,2-dimethylbenzene (**2i′**, 84.4 mg, 0.60 mmol, 60%) as a light yellow liquid. **2i**: ^1^H NMR (500 MHz, CDCl_3_) δ 7.10–7.06 (m, 3H), 2.26 (s, 3H), 2.25 (s, 3H); ^13^C­{^1^H} NMR (126 MHz, CDCl_3_) δ 138.2, 134.8, 131.1, 130.7, 129.3, 125.6, 19.6, 19.1. **2i′**: ^1^H NMR (500 MHz, CDCl_3_) δ 7.24 (d, *J* = 8.5 Hz, 1H), 7.16 (d, *J* = 8.5 Hz, 2H), 2.35 (s, 3H), 2.34 (s, 3H); ^13^C­{^1^H} NMR (126 MHz, CDCl_3_) δ 138.4, 136.1, 131.0, 128.1, 126.8, 126.3, 20.8, 16.1. The spectroscopic data were consistent with the reported values.^42^

#### 1-Chloro-2,4-dimethylbenzene (**2j**)

Nitric acid (70%, 180.0 mg, 2.0 mmol) was added to a mixture of *m*-xylene (**1i**, 106.2 mg, 1.0 mmol), sodium chloride (87.7 mg, 1.5 mmol), and glacial acetic acid (1.5 mL). The reaction mixture was stirred at 50 °C under an oxygen atmosphere (balloon) for 16 h, water (3 mL) added, and the mixture extracted with ethyl acetate (3 × 5 mL). The combined organic layers were dried with sodium sulfate, filtered, and concentrated. The crude product was purified by column chromatography (SiO_2_, 1:3 EtOAc/hexanes, *R*_*f*_ = 0.8) to give **2j** (88.6 mg, 0.63 mmol, 63%) as a light yellow liquid: ^1^H NMR (300 MHz, CDCl_3_) δ 7.18 (d, *J* = 8.0 Hz, 1H), 7.04 (s, 1H), 6.90 (d, *J* = 8.0 Hz, 1H), 2.31 (s, 3H), 2.26 (s, 3H); ^13^C­{^1^H} NMR (75 MHz, CDCl_3_) δ 136.2, 135.5, 131.6, 128.7, 128.3, 127.7, 20.7, 19.9. The spectroscopic data were consistent with the reported values.^42^

#### 2-Chloro-1,4-dimethylbenzene (**2k**)

Nitric acid (70%, 180.0 mg, 2.0 mmol) was added to a mixture of *p*-xylene (**1k**, 106.2 mg, 1.0 mmol), sodium chloride (87.7 mg, 1.5 mmol), and glacial acetic acid (1.5 mL). The reaction mixture was stirred at 50 °C under an oxygen atmosphere (balloon) for 16 h, water (3 mL) added, and the mixture extracted with ethyl acetate (3 × 5 mL). The combined organic layers were dried with sodium sulfate, filtered, and concentrated. The crude product was purified by column chromatography (SiO_2_, 1:3 EtOAc/hexanes, *R*_*f*_ = 0.8) to give **2k** (85.3 mg, 0.61 mmol, 61%) as a light yellow liquid: ^1^H NMR (300 MHz, CDCl_3_) δ 7.16 (s, 1H), 7.09 (d, *J* = 7.5 Hz, 1H), 6.95 (d, *J* = 7.5 Hz, 1H), 2.34 (s, 3H), 2.29 (s, 3H); ^13^C­{^1^H} NMR (75 MHz, CDCl_3_) δ 136.9, 133.9, 132.6, 130.6, 129.4, 127.3, 20.5, 19.4. The spectroscopic data were consistent with the reported values.^42^

#### 2-Chloro-1,3,5-trimethylbenzene (**2l**)

The procedure to prepare **2a** was followed. Starting with NaCl (87.7 mg, 1.5 mmol), acetic acid (1.5 mL), 1,3,5-trimethylbenzene (**1l**, 120.2 mg, 1.0 mmol), sulfuric acid (98.1 mg, 1.0 mmol), and HNO_3_ (45.0 mg, 0.50 mmol), product **2l** (117.2 mg, 0.76 mmol, 76%) was harvested after column chromatography (SiO_2_, 1:9 EtOAc/hexanes, *R*_*f*_ = 0.85) as a colorless liquid: ^1^H NMR (300 MHz, CDCl_3_) δ 6.91 (s, 2H), 2.36 (s, 6H), 2.27 (s, 3H); ^13^C­{^1^H} NMR (75 MHz, CDCl_3_) δ 135.8, 135.5, 131.5, 129.1, 20.6, 20.5. The spectroscopic data were consistent with the reported values.^42^

#### 1-Chloro-2,4,5-trimethylbenzene (**2m**)

*tert*-Butyl nitrite (103.1 mg, 1.0 mmol) was added to a mixture of 1,2,4-trimethylbenzene (**1m**, 120.2 mg, 1.0 mmol) and hydrogen chloride (6.3 wt % in acetic acid, 1.9 mL, 3.6 mmol). The reaction mixture was stirred at 50 °C under an oxygen atmosphere (balloon) for 16 h, water (3 mL) added, and the mixture extracted with ethyl acetate (3 × 5 mL). The combined organic layers were dried with sodium sulfate, filtered, and concentrated. The crude product was purified by column chromatography (SiO_2_, 1:9 EtOAc/hexanes, *R*_*f*_ = 0.8) to give **2m** (100.5 mg, 0.65 mmol, 65%) as a light yellow liquid: ^1^H NMR (300 MHz, CDCl_3_) δ 7.06 (s, 1H), 6.99 (s, 1H), 2.31 (s, 3H), 2.26 (s, 6H); ^13^C­{^1^H} NMR (75 MHz, CDCl_3_) δ 136.3, 135.1, 133.3, 130.4, 129.5, 126.4, 20.9, 19.6, 19.2; HRMS (ESI) *m*/*z* [M + H]^+^ calcd for C_9_H_12_Cl 155.0622, found 155.0612. Under the standard conditions, a 1:1 mixture (108.4 mg, 0.63 mmol) of **2m** and 1,3-dichloro-2,4,5-trimethylbenzene (**2m′**) was produced. **2m′**: ^1^H NMR (300 MHz, CDCl_3_) δ 7.01 (s, 1H), 2.41 (s, 3H), 2.39 (s, 3H), 2.33 (s, 3H); ^13^C­{^1^H} NMR (75 MHz, CDCl_3_) δ 134.4, 133.6, 131.0, 129.1, 127.5, 126.0, 20.8, 20.6, 16.6; HRMS (ESI) *m*/*z* [M + H]^+^ calcd for C_9_H_11_Cl_2_ 189.0232, found 189.0240.

#### 1-Chloro-2,3,4-trimethylbenzene (**2n**)

The procedure to prepare **2a** was followed. Starting with NaCl (87.7 mg, 1.5 mmol), acetic acid (1.5 mL), 1,2,3-trimethylbenzene (**1n**, 120.2 mg, 1.0 mmol), sulfuric acid (98.1 mg, 1.0 mmol), and HNO_3_ (45.0 mg, 0.50 mmol), product **2n** (107.6 mg, 0.70 mmol, 70%) was harvested after column chromatography (SiO_2_, 1:9 EtOAc/hexanes, *R*_*f*_ = 0.8) as a light yellow liquid: ^1^H NMR (300 MHz, CDCl_3_) δ 7.16 (d, *J* = 8.1 Hz, 1H), 6.96 (d, *J* = 8.1 Hz, 1H), 2.40 (s, 3H), 2.30 (s, 3H), 2.26 (s, 3H); ^13^C­{^1^H} NMR (75 MHz, CDCl_3_) δ 136.8, 134.8, 133.9, 132.0, 128.0, 126.0, 20.5, 16.7, 16.4; HRMS (ESI) *m*/*z* [M + H]^+^ calcd for C_9_H_12_Cl 155.0622, found 155.0632.

#### 3-Chloro-1,2,4,5-tetramethylbenzene (**2o**)

The procedure to prepare **2a** was followed. Starting with NaCl (87.7 mg, 1.5 mmol), acetic acid (1.5 mL), 1,2,4,5-tetramethylbenzene (**1o**, 134.2 mg, 1.0 mmol), sulfuric acid (98.1 mg, 1.0 mmol), and HNO_3_ (45.0 mg, 0.50 mmol), product **2o** (151.8 mg, 0.90 mmol, 90%) was harvested after column chromatography (SiO_2_, 1:9 EtOAc/hexanes, *R*_*f*_ = 0.7) as a colorless solid: mp 48.0–50.0 °C; ^1^H NMR (300 MHz, CDCl_3_) δ 6.87 (s, 1H), 2.32 (s, 6H), 2.27 (s, 6H); ^13^C­{^1^H} NMR (75 MHz, CDCl_3_) δ 134.6, 133.0, 131.9, 129.4, 20.5, 16.8. The spectroscopic data were consistent with the reported values.^46^

#### 1-Chloronaphthalene (**2p**)

Nitric acid (70%, 135.0 mg, 1.5 mmol) was added to a mixture of naphthalene (**1p**, 128.2 mg, 1.0 mmol), sodium chloride (87.7 mg, 1.5 mmol), and glacial acetic acid (1.5 mL). The reaction mixture was stirred at 50 °C under an oxygen atmosphere (balloon) for 16 h, water (3 mL) added, and the mixture extracted with ethyl acetate (3 × 5 mL). The combined organic layers were dried with sodium sulfate, filtered, and concentrated. The crude product was purified by column chromatography (SiO_2_, 1:9 EtOAc/hexanes, *R*_*f*_ = 0.8) to give **2p** (109.3 mg, 0.67 mmol, 67%) as a colorless liquid: ^1^H NMR (300 MHz, CDCl_3_) δ 8.36 (d, *J* = 6.9 Hz, 1H), 7.89 (d, *J* = 4.8 Hz, 1H), 7.80 (d, *J* = 4.9 Hz, 1H), 7.66–7.63 (m, 2H), 7.60–7.55 (m, 1H), 7.42 (t, *J* = 4.9 Hz, 1H); ^13^C­{^1^H} NMR (75 MHz, CDCl_3_) δ 135.5, 131.9, 130.8, 128.2, 127.1, 127.0, 126.6, 126.1, 125.6, 124.3. The spectroscopic data were consistent with the reported values.^47^

#### 3-Chloro-4-methoxybenzoic Acid (**2q**)

Nitric acid (70%, 90.0 mg, 1.0 mmol) was added to a mixture of *p*-anisic acid (**1q**, 152.2 mg, 1.0 mmol), sodium chloride (87.7 mg, 1.5 mmol), sulfuric acid (147.1 mg, 1.5 mmol), and glacial acetic acid (1.5 mL). The reaction mixture was stirred at 50 °C (oil bath) under an oxygen atmosphere (balloon) for 16 h, water (3 mL) added, and the mixture extracted with ethyl acetate (3 × 5 mL). The combined organic layers were dried with sodium sulfate, filtered, and concentrated. The crude product was purified by column chromatography (SiO_2_, 1:3 EtOAc/hexanes, *R*_*f*_ = 0.6) to give **2q** (177.3 mg, 0.95 mmol, 95%) as a colorless solid: mp 210.0–213.0 °C; ^1^H NMR (300 MHz, *d*_6_-DMSO) δ 7.87–7.85 (m, 2H), 7.18 (d, *J* = 9.3 Hz, 1H), 4.49 (brs, 1H), 3.89 (s, 3H); ^13^C­{^1^H} NMR (75 MHz, *d*_6_-DMSO) δ 166.3, 158.2, 130.9, 130.3, 124.0, 121.2, 112.5, 56.6. The spectroscopic data were consistent with the reported values.^8a^

#### Methyl 3-Chloro-4-methoxybenzoate (**2r**)

Nitric acid (70%, 90.0 mg, 1.0 mmol) was added to a mixture of methyl 4-methoxybenzoate (**1r**, 166.2 mg, 1.0 mmol), sodium chloride (87.7 mg, 1.5 mmol), sulfuric acid (147.1 mg, 1.5 mmol), and glacial acetic acid (1.5 mL). The reaction mixture was stirred at 80 °C (oil bath) under an oxygen atmosphere (balloon) for 16 h, water (3 mL) added, and the mixture extracted with ethyl acetate (3 × 5 mL). The combined organic layers were dried with sodium sulfate, filtered, and concentrated. The crude product was purified by column chromatography (SiO_2_, 1:3 EtOAc/hexanes, *R*_*f*_ = 0.7) to give **2r** (196.6 mg, 0.98 mmol, 98%) as a colorless solid: mp 97.0–99.0 °C; ^1^H NMR (300 MHz, CDCl_3_) δ 7.97 (d, *J* = 2.1 Hz, 1H), 7.86 (dd, *J* = 8.7, 2.1 Hz, 1H), 6.88 (d, *J* = 8.7 Hz, 1H), 3.89 (s, 3H), 3.84 (s, 3H); ^13^C­{^1^H} NMR (75 MHz, CDCl_3_) δ 165.6, 158.4, 131.4, 129.7, 123.1, 122.2, 111.0, 56.1, 51.9. The spectroscopic data were consistent with the reported values.^15c^

#### 3-Chloro-2-methoxybenzoic Acid (**2s**)

Nitric acid (70%, 90.0 mg, 1.0 mmol) was added to a mixture of 4-methoxybenzoic acid (**1s**, 152.2 mg, 1.0 mmol), sodium chloride (87.7 mg, 1.5 mmol), sulfuric acid (147.1 mg, 1.5 mmol), and glacial acetic acid (1.5 mL). The reaction mixture was stirred at 80 °C (oil bath) under an oxygen atmosphere (balloon) for 16 h, water (3 mL) added, and the mixture extracted with ethyl acetate (3 × 5 mL). The combined organic layers were dried with sodium sulfate, filtered, and concentrated. The crude product was purified by column chromatography (SiO_2_, 3:7 EtOAc/hexanes, *R*_*f*_ = 0.3) to give **2s** (149.3 mg, 0.80 mmol, 80%) as a colorless solid: mp 139.0–141.0 °C; ^1^H NMR (300 MHz, CDCl_3_) δ 10.05 (brs, 1H), 7.96 (d, *J* = 7.8 Hz, 1H), 7.62 (d, *J* = 7.8 Hz, 1H), 7.20 (t, *J* = 7.8 Hz, 1H), 4.03 (s, 3H); ^13^C­{^1^H} NMR (75 MHz, CDCl_3_) δ 167.8, 155.9, 135.6, 131.1, 130.0, 125.2, 124.5, 62.5. The spectroscopic data were consistent with the reported values.^48^ When nitric acid (180.0 mg, 2.0 mmol) was applied without sulfuric acid, a 1:3.5 mixture (184.7 mg, 0.99 mmol, 99%) of **2s** and 5-chloro-2-methoxybenzoic acid (**2s′**) was produced. **2s′**: ^1^H NMR (300 MHz, CDCl_3_) δ 10.09 (brs, 1H), 7.97 (d, *J* = 2.4 Hz, 1H), 7.44 (dd, *J* = 8.8, 2.4 Hz, 1H), 6.97 (d, *J* = 8.8 Hz, 1H), 3.99 (s, 3H); ^13^C­{^1^H} NMR (75 MHz, CDCl_3_) δ 165.8, 157.0, 134.5, 132.6, 126.6, 118.7, 113.2, 56.8. The spectroscopic data were consistent with the reported values.^49^

#### Methyl 5-Chloro-2-methoxybenzoate (**2t**)

Nitric acid (70%, 90.0 mg, 1.0 mmol) was added to a mixture of methyl 2-methoxybenzoate (**1t**, 166.2 mg, 1.0 mmol), sodium chloride (87.7 mg, 1.5 mmol), sulfuric acid (147.1 mg, 1.5 mmol), and glacial acetic acid (1.5 mL). The reaction mixture was stirred at 80 °C (oil bath) under an oxygen atmosphere (balloon) for 16 h, water (3 mL) added, and the mixture extracted with ethyl acetate (3 × 5 mL). The combined organic layers were dried with sodium sulfate, filtered, and concentrated. The crude product was purified by column chromatography (SiO_2_, 1:5 EtOAc/hexanes, *R*_*f*_ = 0.75) to give **2t** (184.6 mg, 0.92 mmol, 92%) as a colorless liquid: ^1^H NMR (300 MHz, CDCl_3_) δ 7.70 (d, *J* = 2.5 Hz, 1H), 7.34 (dd, *J* = 9.0, 2.5 Hz, 1H), 6.85 (d, *J* = 9.0 Hz, 1H), 3.83 (s, 3H), 3.82 (s, 3H); ^13^C­{^1^H} NMR (75 MHz, CDCl_3_) δ 165.2, 157.5, 132.9, 131.1, 124.9, 120.9, 113.2, 56.1, 52.0. The spectroscopic data were consistent with the reported values.^50^

#### 2-Chloro-5-methoxybenzoic Acid (**2u**)

Nitric acid (70%, 90.0 mg, 1.0 mmol) was added to a mixture of 3-methoxybenzoic acid (**1u**, 152.2 mg, 1.0 mmol), sodium chloride (87.7 mg, 1.5 mmol), sulfuric acid (147.1 mg, 1.5 mmol), and glacial acetic acid (1.5 mL). The reaction mixture was stirred at 50 °C (oil bath) under an oxygen atmosphere (balloon) for 16 h, water (3 mL) added, and the mixture extracted with ethyl acetate (3 × 5 mL). The combined organic layers were dried with sodium sulfate, filtered, and concentrated. The crude product was purified by column chromatography (SiO_2_, 1:1 EtOAc/hexanes, *R*_*f*_ = 0.5) to give **2u** and 2-chloro-3-methoxybenzoic acid (**2u′**, 2:1, 130.1 mg, 0.70 mmol, 70%) as a colorless solid. **2u**: ^1^H NMR (300 MHz, CDCl_3_) δ 9.84 (brs, 1H), 7.53 (d, *J* = 3.2 Hz, 1H), 7.38 (d, *J* = 8.7 Hz, 1H), 7.03 (dd, *J* = 8.7, 3.2 Hz, 1H), 3.84 (s, 3H); ^13^C­{^1^H} NMR (75 MHz, CDCl_3_) δ 170.9, 157.9, 132.2, 127.1, 120.1, 116.7, 113.4, 55.6. **2u′**: ^1^H NMR (300 MHz, CDCl_3_) δ 9.84 (brs, 1H), 7.57 (d, *J* = 8.1 Hz, 1H), 7.31 (d, *J* = 8.1 Hz, 1H), 7.13 (d, *J* = 8.1 Hz, 1H), 3.95 (s, 3H); ^13^C­{^1^H} NMR (75 MHz, CDCl_3_) δ 171.5, 155.9, 130.3, 126.0, 123.4, 115.6, 113.0, 56.5. The spectroscopic data were consistent with the reported values.^30^

#### Methyl 2-Chloro-5-methoxybenzoate (**2v**)

Nitric acid (70%, 90.0 mg, 1.0 mmol) was added to a mixture of 3-methoxybenzoate (**1v**, 166.2 mg, 1.0 mmol), sodium chloride (87.7 mg, 1.5 mmol), sulfuric acid (147.1 mg, 1.5 mmol), and glacial acetic acid (1.5 mL). The reaction mixture was stirred at 50 °C (oil bath) under an oxygen atmosphere (balloon) for 16 h, water (3 mL) added, and the mixture extracted with ethyl acetate (3 × 5 mL). The combined organic layers were dried with sodium sulfate, filtered, and concentrated. The crude product was purified by column chromatography (SiO_2_, 1:1 EtOAc/hexanes, *R*_*f*_ = 0.5) to give **2v** (135.1 mg, 0.72 mmol, 72%) as a colorless liquid: ^1^H NMR (300 MHz, CDCl_3_) δ 7.29–7.27 (m, 2H), 6.91 (dd, *J* = 9.0, 2.7 Hz, 1H), 3.88 (s, 3H), 3.77 (s, 3H); ^13^C­{^1^H} NMR (75 MHz, CDCl_3_) δ 165.8, 157.8, 131.7, 127.0, 122.2, 118.7, 115.9, 55.5, 52.3. The spectroscopic data were consistent with the reported values.^9c^

#### 1-(3-Chloro-4-methoxyphenyl)­ethan-1-one (**2w**)

Nitric acid (70%, 90.0 mg, 1.0 mmol) was added to a mixture of 4-methoxyacetophenone (**1w**, 150.2 mg, 1.0 mmol), sodium chloride (87.7 mg, 1.5 mmol), sulfuric acid (147.1 mg, 1.5 mmol), and glacial acetic acid (1.5 mL). The reaction mixture was stirred at 50 °C (oil bath) under an oxygen atmosphere (balloon) for 16 h, water (3 mL) added, and the mixture extracted with ethyl acetate (3 × 5 mL). The combined organic layers were dried with sodium sulfate, filtered, and concentrated. The crude product was purified by column chromatography (SiO_2_, 1:9 EtOAc/hexanes, *R*_*f*_ = 0.7) to give **2w** (73.8 mg, 0.40 mmol, 40%) as a colorless liquid: ^1^H NMR (300 MHz, CDCl_3_) δ 7.68 (d, *J* = 3.5 Hz, 1H), 7.57 (dd, *J* = 8.5, 3.5 Hz, 1H), 6.70 (d, *J* = 8.5 Hz, 1H), 3.71 (s, 3H), 2.31 (s, 3H); ^13^C­{^1^H} NMR (75 MHz, CDCl_3_) δ 194.9, 158.0, 130.0, 129.7, 128.3, 122.0, 110.7, 55.7, 25.6. The spectroscopic data were consistent with the reported values.^10b^

#### 3-Chloro-4-methoxybenzaldehyde (**2x**)

Nitric acid (70%, 90.0 mg, 1.0 mmol) was added to a mixture of 4-methoxybenzaldehyde (**1x**, 136.2 mg, 1.0 mmol), sodium chloride (87.7 mg, 1.5 mmol), sulfuric acid (147.1 mg, 1.5 mmol), and glacial acetic acid (1.5 mL). The reaction mixture was stirred at 80 °C (oil bath) under an oxygen atmosphere (balloon) for 16 h, water (3 mL) added, and the mixture extracted with ethyl acetate (3 × 5 mL). The combined organic layers were dried with sodium sulfate, filtered, and concentrated. The crude product was purified by column chromatography (SiO_2_, 1:1 EtOAc/hexanes, *R*_*f*_ = 0.9) to give **2x** (102.4 mg, 0.60 mmol, 60%) as a colorless liquid: ^1^H NMR (300 MHz, CDCl_3_) δ 9.80 (s, 1H), 7.84 (d, *J* = 2.0 Hz, 1H), 7.73 (dd, *J* = 8.5, 2.0 Hz, 1H), 7.01 (d, *J* = 8.5 Hz, 1H), 3.95 (s, 3H); ^13^C­{^1^H} NMR (75 MHz, CDCl_3_) δ 189.6, 159.6, 130.9, 130.5, 130.1, 123.5, 111.6, 56.4. The spectroscopic data were consistent with the reported values.^12c^

#### *N*-(4-Chlorophenyl)­acetamide (**2y**)

Nitric acid (70%, 90.0 mg, 1.0 mmol) was added to a mixture of acetanilide (**1y**, 135.2 mg, 1.0 mmol), sodium chloride (87.7 mg, 1.5 mmol), sulfuric acid (147.1 mg, 1.5 mmol), and glacial acetic acid (1.5 mL). The reaction mixture was stirred at 50 °C (oil bath) under an oxygen atmosphere (balloon) for 16 h, water (3 mL) added, and the mixture extracted with ethyl acetate (3 × 5 mL). The combined organic layers were dried with sodium sulfate, filtered, and concentrated. The crude product was purified by column chromatography (SiO_2_, 1:9 EtOAc/hexanes, *R*_*f*_ = 0.4) to give **2y** (84.8 mg, 0.50 mmol, 50%) as a colorless liquid: ^1^H NMR (500 MHz, CDCl_3_/*d*_6_-DMSO) δ 7.45 (d, *J* = 8.5 Hz, 2H), 7.27 (d, *J* = 8.5 Hz, 2H), 7.20 (br, 1H), 2.17 (s, 3H); ^13^C­{^1^H} NMR (126 MHz, CDCl_3_/*d*_6_-DMSO) δ 168.9, 137.4, 128.6, 128.3, 121.0, 24.2. The spectroscopic data were consistent with the reported values.^51^

#### *N*-(4-Chlorophenyl)-*N*-methylacetamide (**2z**)

Nitric acid (70%, 90.0 mg, 1.0 mmol) was added to a mixture of *N*-methylacetanilide (**1z**, 75.0 mg, 0.50 mmol), sodium chloride (58.4 mg, 2.0 mmol), sulfuric acid (73.5 mg, 0.75 mmol), and glacial acetic acid (1.5 mL). The reaction mixture was stirred at 50 °C (oil bath) under an oxygen atmosphere (balloon) for 16 h, water (3 mL) added, and the mixture extracted with ethyl acetate (3 × 5 mL). The combined organic layers were dried with sodium sulfate, filtered, and concentrated. The crude product was purified by column chromatography (SiO_2_, 1:3 EtOAc/hexanes, *R*_*f*_ = 0.4) to give **2z** (38.0 mg, 0.20 mmol, 40%) as a light yellow liquid: ^1^H NMR (300 MHz, CDCl_3_) δ 7.37 (d, *J* = 8.4 Hz, 2H), 7.13 (d, *J* = 8.4 Hz, 2H), 3.23 (s, 3H), 1.86 (s, 3H); ^13^C­{^1^H} NMR (75 MHz, CDCl_3_) δ 170.3, 143.1, 133.5, 129.9, 128.4, 37.1, 22.3. It was difficult to spearate **2z** from the starting material; however, the spectroscopic data were consistent with the reported values.^52^

#### 3-Chloro-4-methoxyphenylacetic Acid (**2aA**)

The procedure to prepare **2a** was followed. Starting with NaCl (87.7 mg, 1.5 mmol), acetic acid (1.5 mL), 4-methoxyphenylacetic acid (**1aA**, 166.2 mg, 1.0 mmol), sulfuric acid (98.1 mg, 1.0 mmol), and HNO_3_ (45.0 mg, 0.50 mmol), product **2aA** (185.1 mg, 0.92 mmol, 92%) was harvested after column chromatography (SiO_2_, 1:9 EtOAc/hexanes, *R*_*f*_ = 0.2) as a light yellow solid: mp 91.0–93.0 °C; ^1^H NMR (300 MHz, CDCl_3_) δ 8.78 (br, 1H), 7.30 (d, *J* = 2.1 Hz, 1H), 7.13 (dd, *J* = 8.4, 2.1 Hz, 1H), 6.88 (d, *J* = 8.4 Hz, 1H), 3.89 (s, 3H), 3.57 (s, 2H); ^13^C­{^1^H} NMR (75 MHz, CDCl_3_) δ 177.6, 154.3, 131.1, 128.7, 126.2, 122.5, 112.1, 56.1, 39.7. The spectroscopic data were consistent with the reported values.^52^

#### Methyl 2-(3-Chloro-4-methoxyphenyl)­acetate (**2aE**)

The procedure to prepare **2a** was followed. Starting with NaCl (87.7 mg, 1.5 mmol), acetic acid (1.5 mL), methyl 2-(4-methoxyphenyl)­acetate (**1aE**, 180.2 mg, 1.0 mmol), sulfuric acid (98.1 mg, 1.0 mmol), and HNO_3_ (45.0 mg, 0.50 mmol), product **2aE** (189.1 mg, 0.88 mmol, 88%) was harvested after column chromatography (SiO_2_, 1:9 EtOAc/hexanes, *R*_*f*_ = 0.3) as a light yellow liquid: ^1^H NMR (300 MHz, CDCl_3_) δ 7.29 (d, *J* = 2.1 Hz, 1H), 8.13 (dd, *J* = 8.5, 2.1 Hz, 1H), 6.87 (d, *J* = 8.5 Hz, 1H), 3.88 (s, 3H), 3.69 (s, 3H), 3.54 (s, 2H); ^13^C­{^1^H} NMR (75 MHz, CDCl_3_) δ 171.1, 154.1, 131.0, 128.5, 127.0, 122.3, 112.0, 56.1, 52.1, 39.8; HRMS (ESI) *m*/*z* [M + Na]^+^ calcd for C_10_H_11_O_3_ClNa 237.0289, found 237.0294.

#### 3-Chloro-4-methoxybenzonitrile (**2aN**)

Nitric acid (70%, 90.0 mg, 1.0 mmol) was added to a mixture of 4-methoxybenzonitrile (**1aN**, 133.2 mg, 1.0 mmol), sodium chloride (87.7 mg, 1.5 mmol), sulfuric acid (147.1 mg, 1.5 mmol), and glacial acetic acid (1.5 mL). The reaction mixture was stirred at 80 °C (oil bath) under an oxygen atmosphere (balloon) for 16 h, water (3 mL) added, and the mixture extracted with ethyl acetate (3 × 5 mL). The combined organic layers were dried with sodium sulfate, filtered, and concentrated. The crude product was purified by column chromatography (SiO_2_, 1:3 EtOAc/hexanes, *R*_*f*_ = 0.7) to give **2aN** (125.7 mg, 0.75 mmol, 75%) as a colorless solid: mp 105.0–108.0 °C; ^1^H NMR (300 MHz, CDCl_3_) δ 7.57 (d, *J* = 1.9 Hz, 1H), 7.50 (dd, *J* = 8.7, 1.9 Hz, 1H), 6.95 (d, *J* = 8.7 Hz, 1H), 3.92 (s, 3H); ^13^C­{^1^H} NMR (75 MHz, CDCl_3_) δ 158.4, 133.2, 132.3, 123.2, 117.7, 112.1, 104.4, 56.3. The spectroscopic data were consistent with the reported values.^53^

#### 4-Chloro-1*H*-pyrazole (**2pr**)

The procedure to prepare **2a** was followed. Starting with NaCl (87.7 mg, 1.5 mmol), acetic acid (1.5 mL), 1*H*-pyrazole (68.1 mg, 1.0 mmol), sulfuric acid (98.1 mg, 1.0 mmol), and HNO_3_ (45.0 mg, 0.50 mmol), product **2pr** (97.4 mg, 0.95 mmol, 95%) was harvested after concentration as a colorless solid: mp 75.0–77.0 °C; ^1^H NMR (300 MHz, CDCl_3_) δ 9.75 (br, 1H), 7.55 (s, 2H); ^13^C­{^1^H} NMR (75 MHz, CDCl_3_) δ 132.1, 110.4. The spectroscopic data were consistent with the reported values.^54^

#### 5-Chloro-1-methyl-1*H*-imidazole (**2im**)

The procedure to prepare **2a** was followed. Starting with NaCl (87.7 mg, 1.5 mmol), acetic acid (1.5 mL), 1-methyl-1*H*-imidazole (82.1 mg, 1.0 mmol), sulfuric acid (98.1 mg, 1.0 mmol), and HNO_3_ (45.0 mg, 0.50 mmol), product **2im** (104.9 mg, 0.90 mmol, 90%) was harvested after concentration as a colorless liquid: ^1^H NMR (300 MHz, CDCl_3_) δ 7.30 (s, 1H), 6.92 (s, 1H), 3.56 (s, 3H); ^13^C­{^1^H} NMR (75 MHz, CDCl_3_) δ 137.3, 128.7, 119.7, 32.8; HRMS (ESI) *m*/*z* [M + H]^+^ calcd for C_4_H_6_ClN_2_ 117.0214, found 117.0213. The spectroscopic data were consistent with the reported values.^55^

#### 4-(((Benzyloxy)­carbonyl)­amino)-2-methoxybenzoic Acid (**4**)

Benzyl chloroformate (0.16 mL, 1.1 mmol) was added to a solution of 4-amino-2-methoxybenzoic acid (167.2 mg, 1.0 mmol), THF (4.0 mL), and saturated NaHCO_3(aq)_ (4.0 mL). The reaction mixture was stirred at 25 °C for 4 h and acidified with HCl_(aq)_ (1 N, 10 mL). The organic layer was separated, dried over sodium sulfate, filtered, and concentrated. The crude product was purified by column chromatography (SiO_2_, 1:3 EtOAc/hexanes, *R*_*f*_ = 0.6) to give **4** (299.7 mg, 0.99 mmol, 99%) as a colorless solid: mp 148.0–150.0 °C; ^1^H NMR (300 MHz, *d*_6_-DMSO) δ 10.10 (s, 1H), 7.67 (d, *J* = 8.7 Hz, 1H), 7.44–7.32 (m, 6H), 7.07 (d, *J* = 8.7 Hz, 1H), 5.17 (s, 2H), 3.77 (s, 3H); ^13^C­{^1^H} NMR (75 MHz, *d*_6_-DMSO) δ 166.8, 159.8, 153.4, 144.3, 136.5, 132.7, 128.7, 128.3, 114.1, 109.5, 101.7, 66.2, 55.6; HRMS (ESI) *m*/*z* [M]^+^ calcd for C_16_H_16_NO_5_ 302.1023, found 302.1008.

#### 4-Amino-5-chloro-2-methoxybenzoic Acid (**3**)

Nitric acid (70%, 180.0 mg, 2.0 mmol) was added to a mixture of **4** (167.2 mg, 1.0 mmol), sodium chloride (87.7 mg, 1.5 mmol), and glacial acetic acid (1.5 mL). The reaction mixture was stirred at 80 °C (oil bath) under an oxygen atmosphere (balloon) for 16 h, water (3 mL) added, and the mixture extracted with ethyl acetate (3 × 5 mL). The combined organic layers were dried with sodium sulfate, filtered, and concentrated. The crude product was purified by column chromatography (SiO_2_, 1:5 EtOAc/hexanes, *R*_*f*_ = 0.4) to give 4-(((benzyloxy)­carbonyl)­amino)-5-chloro-2-methoxybenzoic acid (235.0 mg, 0.70 mmol, 70%) as a colorless liquid. The suspension of the above acid (40.0 mg, 0.12 mmol), Pd/C (10%, 12.8 mg, 0.1 mmol), and methanol (1 mL) was stirred under a hydrogen atmosphere (1 atm) for 3 h, filtered, and concentrated. The crude product was purified by column chromatography (SiO_2_, 5:1 EtOAc/methanol, *R*_*f*_ = 0.9) to give **3** (23.0 mg, 0.11 mmol, 95%) as a dark brown liquid: ^1^H NMR (300 MHz, *d*_6_-DMSO) δ 10.98 (br, 1H), 7.54 (s, 1H), 6.38 (s, 1H), 6.00 (s, 2H), 3.67 (s, 3H); ^13^C­{^1^H} NMR (75 MHz, *d*_6_-DMSO) δ 179.4, 165.4, 159.7, 149.7, 132.7, 108.0, 97.8, 55.6; HRMS (ESI) *m*/*z* [M + H]^+^ calcd for C_8_H_9_ClNO_3_ 202.0265, found 202.0267.

#### *N*-(2-Benzoylphenyl)-2-chloroacetamide (**5**)

Chloroacetyl chloride (0.24 mL, 3.0 mmol) was added to a solution of 2-aminobenzophenone (394.5 mg, 2.0 mmol), triethylamine (0.42 mL, 3.0 mmol), and 1,4-dioxane (10 mL) at 0 °C. The reaction mixture was stirred at 25 °C for 30 min, water (15 mL) added, ans the mixture filtered and extracted with ethyl acetate (3 × 5 mL). The combined organic layers were dried with sodium sulfate, filtered, and concentrated. The crude product was purified by column chromatography (SiO_2_, 1:1 EtOAc/hexanes, *R*_*f*_ = 0.9) to give **5** (454.6 mg, 1.90 mmol, 95%) as a light yellow solid: mp 105.0–108.0 °C; ^1^H NMR (300 MHz, CDCl_3_) δ 11.60 (s, 1H), 8.59 (d, *J* = 8.8 Hz, 1H), 7.69 (d, *J* = 6.9 Hz, 2H), 7.55 (d, *J* = 6.9 Hz, 3H), 7.46 (d, *J* = 6.9 Hz, 2H), 7.12 (t, *J* = 8.8 Hz, 1H), 4.16 (s, 2H); ^13^C­{^1^H} NMR (75 MHz, CDCl_3_) δ 198.9, 165.2, 139.0, 138.1, 133.9, 133.3, 132.5, 129.8, 128.1, 123.9, 122.9, 121.3, 43.0. The spectroscopic data were consistent with the reported values.^56^

#### 2-Chloroacetamido-5-chlorobenzophenone (**6**)

Nitric acid (70%, 180.0 mg, 2.0 mmol) was added to a mixture of **5** (136.9 mg, 0.50 mmol) and HCl_(g)_ in glacial acetic acid (6.3 wt %, 1.9 mL, 3.6 mmol). The reaction mixture was stirred at 80 °C under an oxygen atmosphere (balloon) for 16 h, water (3 mL) added, and the mixture extracted with ethyl acetate (3 × 5 mL). The combined organic layers were dried with sodium sulfate, filtered, and concentrated. The crude product was purified by column chromatography (SiO_2_, 1:5 EtOAc/hexanes, *R*_*f*_ = 0.4) to give **6** (100.2 mg, 0.33 mmol, 65%) as a light yellow solid: mp 119.0–120.0 °C; ^1^H NMR (300 MHz, CDCl_3_) δ 11.42 (s, 1H), 8.55 (d, *J* = 9.6 Hz, 1H), 7.70 (d, *J* = 7.2 Hz, 2H), 7.6 (t, *J* = 7.4 Hz, 1H), 7.51–7.45 (m, 4H), 4.16 (s, 2H); ^13^C­{^1^H} NMR (75 MHz, CDCl_3_) δ 197.5, 165.2, 137.5, 137.3, 133.6, 132.9, 132.4, 129.8, 128.4, 128.1, 125.3, 122.8, 42.9. The spectroscopic data were consistent with the reported values.^32^

#### 1,4-Dimethoxy-2-nitrobenzene (**7**)

Nitric acid (70%, 45.0 mg, 0.5 mmol) was added to a mixture of **1g** (138.2 mg, 0.50 mmol), sulfuric acid (98.1 mg, 1.0 mmol), and glacial acetic acid (1.5 mL). The reaction mixture was stirred at 25 °C under an oxygen atmosphere (balloon) for 16 h, water (3 mL) added, and the mixture extracted with ethyl acetate (3 × 5 mL). The combined organic layers were dried with sodium sulfate, filtered, and concentrated. The crude product was purified by column chromatography (SiO_2_, 1:9 EtOAc/hexanes, *R*_*f*_ = 0.3) to give **7** (87.9 mg, 0.48 mmol, 96%) as a light yellow solid and recover **1g** (62.2 mg, 0.45 mmol): mp 68.0–70.0 °C; ^1^H NMR (500 MHz, CDCl_3_) δ 7.32 (d, *J* = 2.7 Hz, 1H), 7.06 (dd, *J* = 9.1, 2.7 Hz, 1H), 7.00 (d, *J* = 9.1 Hz, 1H), 3.86 (s, 3H), 3.76 (s, 3H); ^13^C­{^1^H} NMR (126 MHz, CDCl_3_) δ 152.7, 147.2, 139.4, 120.6, 115.0, 109.9, 56.9, 55.9. The spectroscopic data were consistent with the reported values.^57^

## Supplementary Material





## Data Availability

The data underlying this study are available in the published article and its .

## Notes

Reaction scope studies, including unsuccessful substrates, figures of the starch-iodide test, H_2_O_2_ measurements from the FOX assay and its calibration curve, isotope effect experiments and calculations, and copies of ^1^H and ^13^C­{^1^H} NMR spectra for all synthesized compounds (PDF)
FAIR data, including the primary NMR FID files, for compounds **2a–2z**, **2h′**, **2aA**, **2aE**, **2aN**, **2pr**, **2im**, and **3–7** (ZIP)
