# Supplementary material for: Nitrosonium Ion-Catalyzed Oxidative Chlorination of Arenes
Source: J Org Chem. 2026 Jan 5;91(2):892–903. doi: 10.1021/acs.joc.5c01914 (PMC12814551; doi:10.1021/acs.joc.5c01914)
Supplement: Supplementary file 1 [file jo5c01914_si_001.pdf]

Supporting Information

Nitrosonium Ion Catalyzed Oxidative Chlorination of Arenes.

Ju-Ching Hsu, Yan-Shiun Chen, Chen-Hung Hsiao and Duen-Ren Hou *

Department of Chemistry, National Central University, No. 300 Jhong-Da Rd., Jhong-li, Taoyuan, Taiwan, 320317.

Table of Contents

Table S1. Substrates that did not yield chlorinated products……………………........S6

Figure S1. Starch-Iodide Test …………………………………………………………S7

Measuring the concentration of H_2O_2 after reaction………………………………S8

Figure S2. Corresponding calibration curve of FOX assay for H_2O_2 measurment.S9

Experiments and Calculations for Isotope Effects......................................S10

^{1}H NMR of compounds *p*-**2a** and *o*-**2a**……...S11

^{13}C {^{1}H} NMR of compounds *p*-**2a** and *o*-**2a**..S12

^{1}H NMR of compound **2b**..S13

^{13}C {^{1}H} NMR of compound **2b.**...S14

^{1}H NMR of compound **2c**..S15

^{13}C {^{1}H} NMR of compound **2c**……..S16

^{1}H NMR of compound **2d**..S17

^{13}C {^{1}H} NMR of compound **2d**..……...……S18

^{1}H NMR of compound **2e**…..S19

^{13}C {^{1}H} NMR of compound **2e**…...…………S20

^{1}H NMR of compound **2f**…..S21

^{13}C {^{1}H} NMR of compound **2f**…...……….S22

^{1}H NMR of compound **2g**…..S23

^{13}C {^{1}H} NMR of compound **2g**…...………S24

^{1}H NMR of compound **2h**…..S25

^{13}C {^{1}H} NMR of compound **2h**…..S26

^{1}H NMR of compound **2h'**…..S27

^{13}C {^{1}H} NMR of compound **2h'**……………………………………S28

^{1}H NMR of compounds **2i** and **2i'**……………………………………S29

^{13}C {^{1}H} NMR of compounds **2i** and **2i'**……………………………………S30

^{1}H NMR of compound **2j**……………………………………S31

^{13}C {^{1}H} NMR of compound **2j**……………………………………S32

^{1}H NMR of compound **2k**……………………………………S33

^{13}C {^{1}H} NMR of compound **2k**……………………………………S34

^{1}H NMR of compound **2l**……………………………………S35

^{13}C {^{1}H} NMR of compound **2l**……………………………………S36

^{1}H NMR of compound **2m**……………………………………S37

^{13}C {^{1}H} NMR of compound **2m**……………………………………S38

^{1}H NMR of compounds **2m** and **2m'**……………………………………S39

^{13}C {^{1}H} NMR of compounds **2m** and **2m'**……………………………………S40

^{1}H NMR of compound **2n**……………………………………S41

^{13}C {^{1}H} NMR of compound **2n**……………………………………S42

^{1}H NMR of compound **2o**……………………………………S43

^{13}C {^{1}H} NMR of compound **2o**……………………………………S44

^{1}H NMR of compound **2p**……………………………………S45

^{13}C {^{1}H} NMR of compound **2p**……………………………………S46

^{1}H NMR of compound **2q**……………………………………S47

^{13}C {^{1}H} NMR of compound **2q**……………………………………S48

^{1}H NMR of compound **2r**……………………………………S49

^{13}C {^{1}H} NMR of compound **2r**……………………………………S50

^{1}H NMR of compound **2s**......S51

^{13}C {^{1}H} NMR of compound **2s**......S52

^{1}H NMR of compounds **2s** and **2s'**......S53

^{13}C {^{1}H} NMR of compounds **2s** and **2s'**......S54

^{1}H NMR of compound **2t**......S55

^{13}C {^{1}H} NMR of compound **2t**......S56

^{1}H NMR of compounds **2u** and **2u'**......S57

^{13}C {^{1}H} NMR of compounds **2u** and **2u'**......S58

^{1}H NMR of compound **2v**......S59

^{13}C {^{1}H} NMR of compound **2v**......S60

^{1}H NMR of compound **2w**......S61

^{13}C {^{1}H} NMR of compound **2w**......S62

^{1}H NMR of compound **2x**......S63

^{13}C {^{1}H} NMR of compound **2x**......S64

^{1}H NMR of compound **2y**......S65

^{13}C {^{1}H} NMR of compound **2y**......S66

^{1}H NMR of compound **2z**......S67

^{13}C {^{1}H} NMR of compound **2z**......S68

^{1}H NMR of compound **2aA**......S69

^{13}C {^{1}H} NMR of compound **2aA**......S70

^{1}H NMR of compound **2aE**......S71

^{13}C {^{1}H} NMR of compound **2aE**......S72

^{1}H NMR of compound **2aN**......S73

^{13}C {^{1}H} NMR of compound **2aN**……………………........…….............................S74

^{1}H NMR of compound **2pr**...….........…...............................….......................S75

^{13}C {^{1}H} NMR of compound **2pr**…………………………........…….........................S76

^{1}H NMR of compound **2im**...….........…...............................….......................S77

^{13}C {^{1}H} NMR of compound **2im**…………………………........…….........................S78

^{1}H NMR of compound **4**....….........…...................................…............................S79

^{13}C {^{1}H} NMR of compound **4**…………………………........…….............................S80

^{1}H NMR of compound **3**....….........…...................................…............................S81

^{13}C {^{1}H} NMR of compound **3**…………………………........…….............................S82

^{1}H NMR of compound **5**....….........…...................................…............................S83

^{13}C {^{1}H} NMR of compound **5**…………………………........…….............................S84

^{1}H NMR of compound **6**....….........…...................................…............................S85

^{13}C {^{1}H} NMR of compound **6**…………………………........…….............................S86

^{1}H NMR of compound **7**....….........…...................................…............................S87

^{13}C {^{1}H} NMR of compound **7**…………………………........…….............................S88

Table S1. Substrates that did not yield chlorinated products.

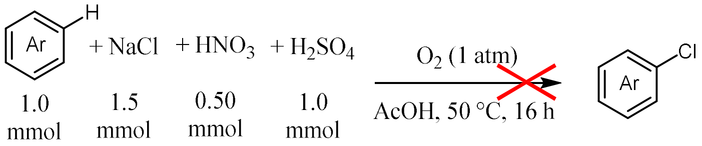

entry	substrate	result
1	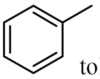 toluene	No reaction; starting material recovered (>90%).
2	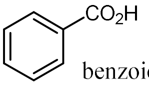 benzoic acid	No reaction; starting material recovered (>90%).
3	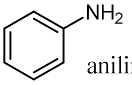 aniline	No reaction; protonated starting material recovered (>90%).
4	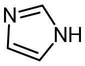 imidazole	No reaction; protonated starting material recovered (>90%).
5	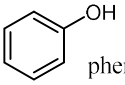 phenol	No reaction; starting material recovered (>90%).
6	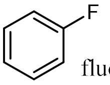 fluorobenzene	No reaction; starting material recovered (>90%).
7	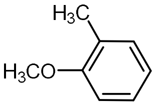 2-methylanisole	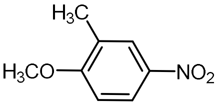 1-methoxy-2-methyl-4-nitrobenzene (0.5 mmol) and starting material recovered.
8	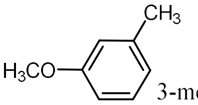 3-methylanisole	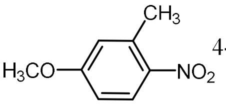 4-methoxy-2-methyl-1-nitrobenzene (0.5 mmol) and starting material recovered.
9	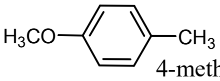 4-methylanisole	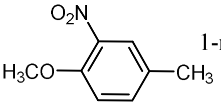 1-methoxy-4-methyl-2-nitrobenzene (0.5 mmol) and starting material recovered.
10	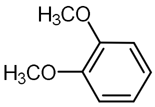 1,2-dimethoxybenzene	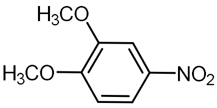 1,2-dimethoxy-4-nitrobenzene (0.5 mmol) and starting material recovered.
11	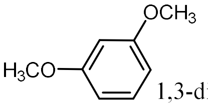 1,3-dimethoxybenzene	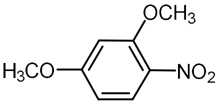 2,4-dimethoxy-1-nitrobenzene (0.5 mmol) and starting material recovered.
12	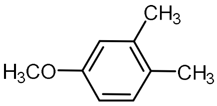 4-methoxy-1,2-dimethylbenzene	Decomposed.

(a) Chlorination reaction

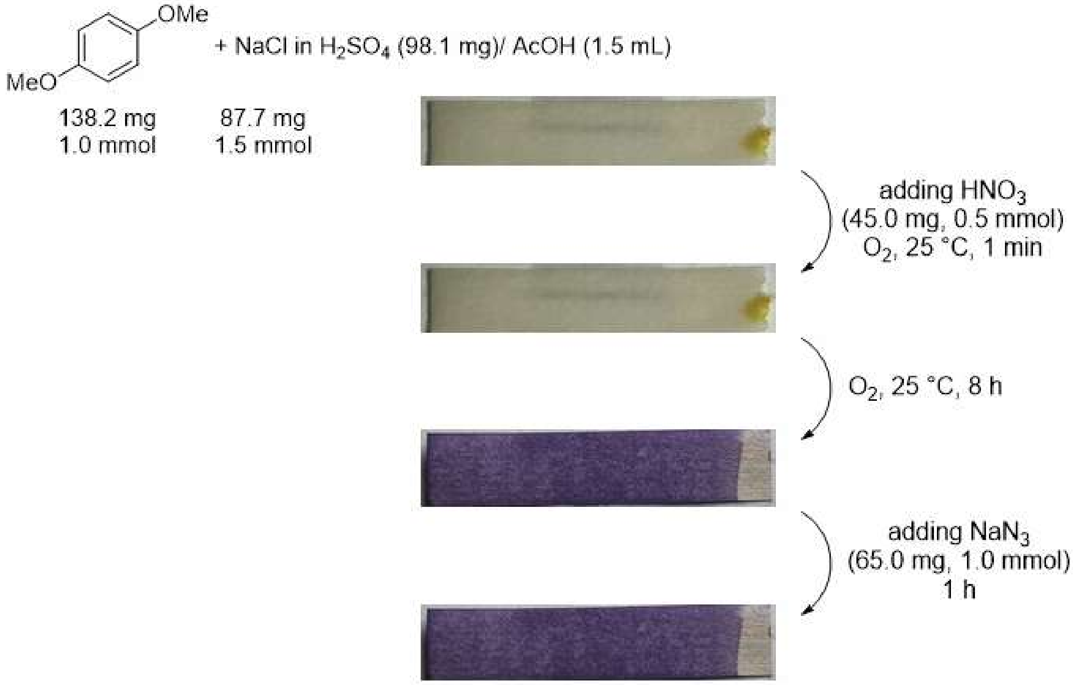

(b) Without NaCl

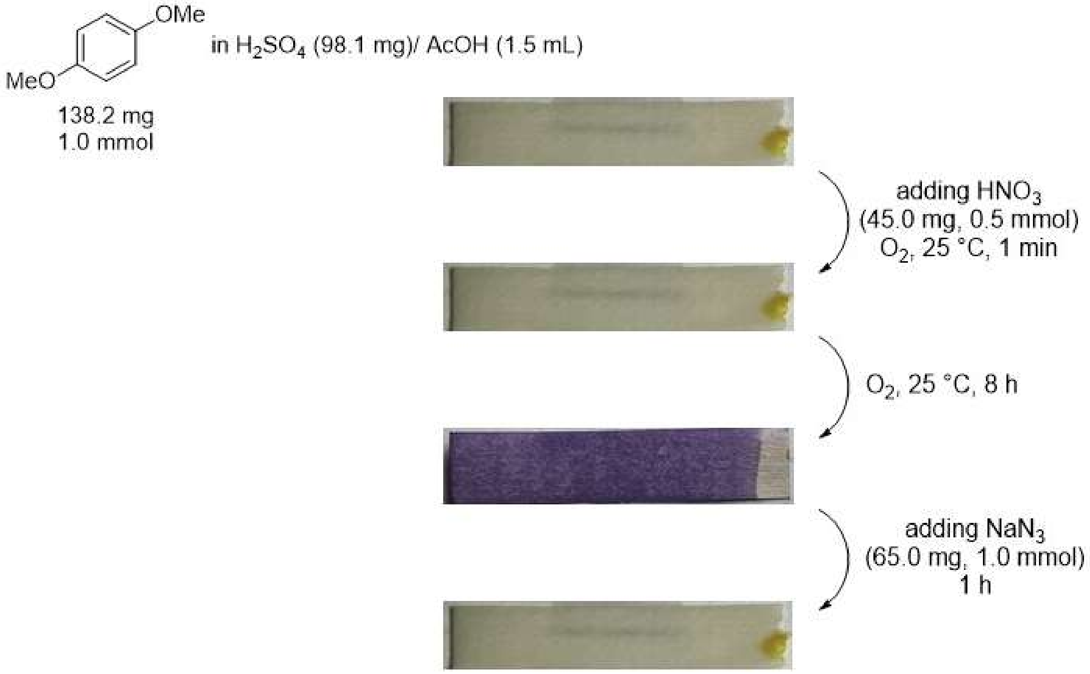

Figure S1. Starch-Iodide Test

Measuring the concentration of H_2O_2 after reaction.

The concentration of hydrogen peroxide (H_2O_2) in the aqueous layer after extraction was determined using the FOX assay. The chlorination reaction of 1,4-dimethoxybenzene (**1g**, 138.2 mg, 1.0 mmol) was carried out as described in the Experimental Section. The resulting aqueous layer (3 mL) was diluted to 10.0 mL in a volumetric flask. An aliquot (0.50 mL) of this solution was further diluted to 100.0 mL. For the assay, 500.0 μL of the final diluted solution was mixed with 500.0 μL of Tris buffer (0.1 M, pH 8.0). Then, 100.0 μL of the resulting mixture was combined with 900.0 μL of FOX reagent (containing 0.1 mM Xylenol Orange, 2.5 mM $(NH_4)_2Fe(SO_4)_2\cdot 6H_2O$, 0.1 M D-sorbitol, and 25 μM H_2SO_4). The mixture was shaken at 25 °C for 30 minutes, and the absorbance at 560 nm was recorded. All measurements were performed in triplicate. The concentration of H_2O_2 after the reaction was 30.4±1 uM × 400 = 12.1±0.4 mM.

H_2O_2 concentration (μM)	0	20	40	60	80	30.4
absorption (a.u.)	0.1038	0.2380	0.3351	0.4767	0.5575	0.2863

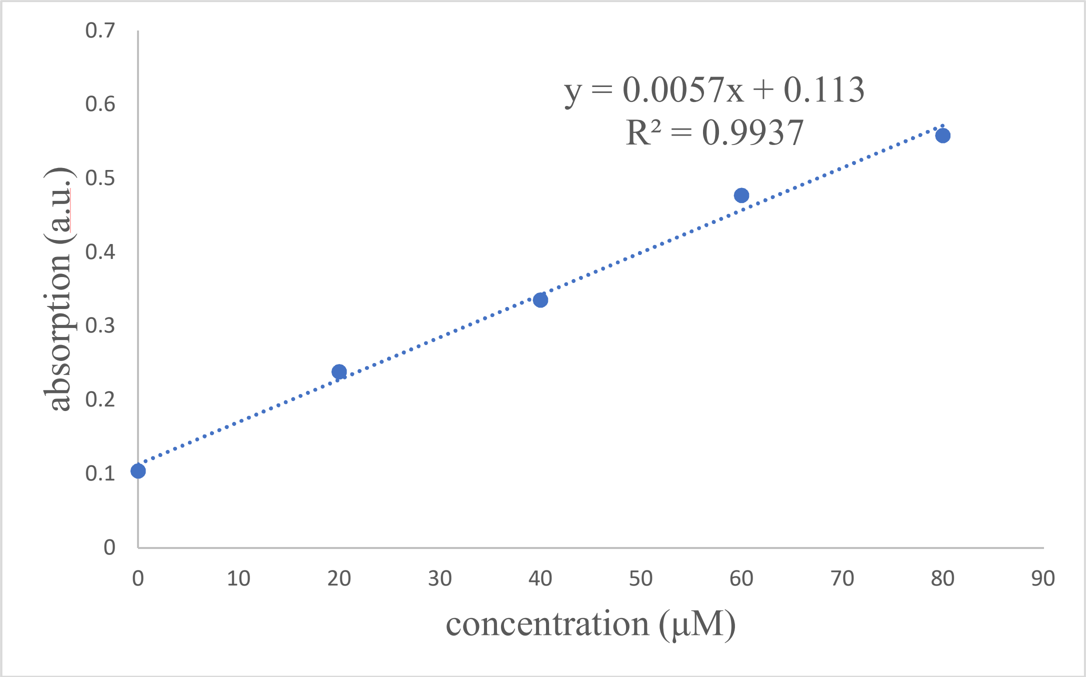

Figure S2. Corresponding calibration curve of FOX assay for H_2O_2 measurment**.**

Experiments and Calculations for Isotope Effects

Nitric acid (70%, 45.0 mg, 0.5 mmol) was added to a mixture of d_5-**1b** (175.2 mg, 1.0 mmol), sodium chloride (29.2 mg, 0.5 mmol), sulfuric acid (98.1 mg, 1.0 mmol) and acetic acid (1.5 mL) in a 5 mL−round bottom flask. The reaction mixture was stirred at 50 ℃ (oil bath) under an atmosphere of oxygen (balloon) for 16 h, add with water (3.0 mL) and extracted with ethyl acetate (5 mL× 3). The combined organic layers were dried with sodium sulfate, filtered and concentrated to give a yellow liquid (215.2 mg), which was analyzed by ^1H and $^{13}C\{^1H\}$ NMR. HRMS of d_4-**2b** (ESI) m/z: calcd for $C_{12}H_6ClD_4O$ $(M+H)^+$ 209.0666, found 209.0659; HRMS of d_5-**2b** (ESI) m/z: calcd for $C_{12}H_5ClD_5O$ $(M+H)^+$ 210.0729, found 210.0725.

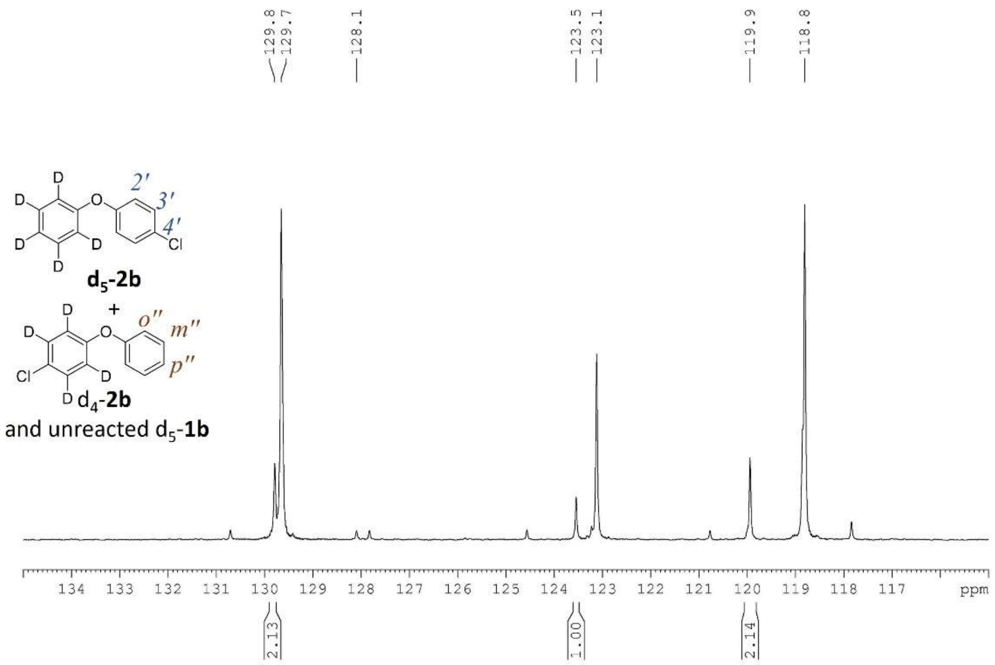

$^{13}C\{^1H\}$ NMR of compound d_5-**1b**, d_5-**2b**, d_4-**2b** (126 MHz, $CDCl_3$)

k_H/k_D = 2.14/(1.0 × 2) = 1.07

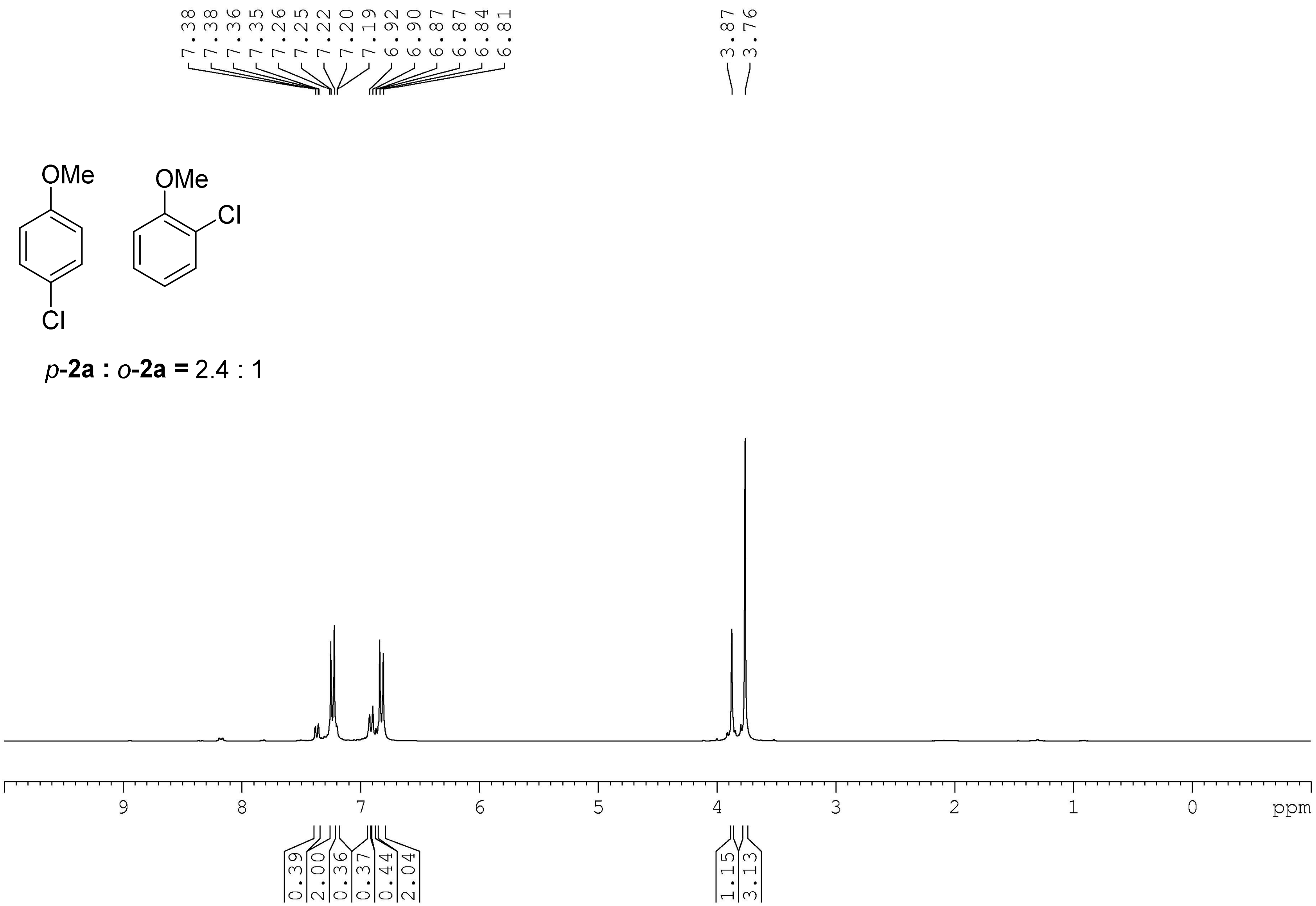

^{1}H NMR of compounds *p*-**2a** and *o*-**2a** (300 MHz, $CDCl_3$)

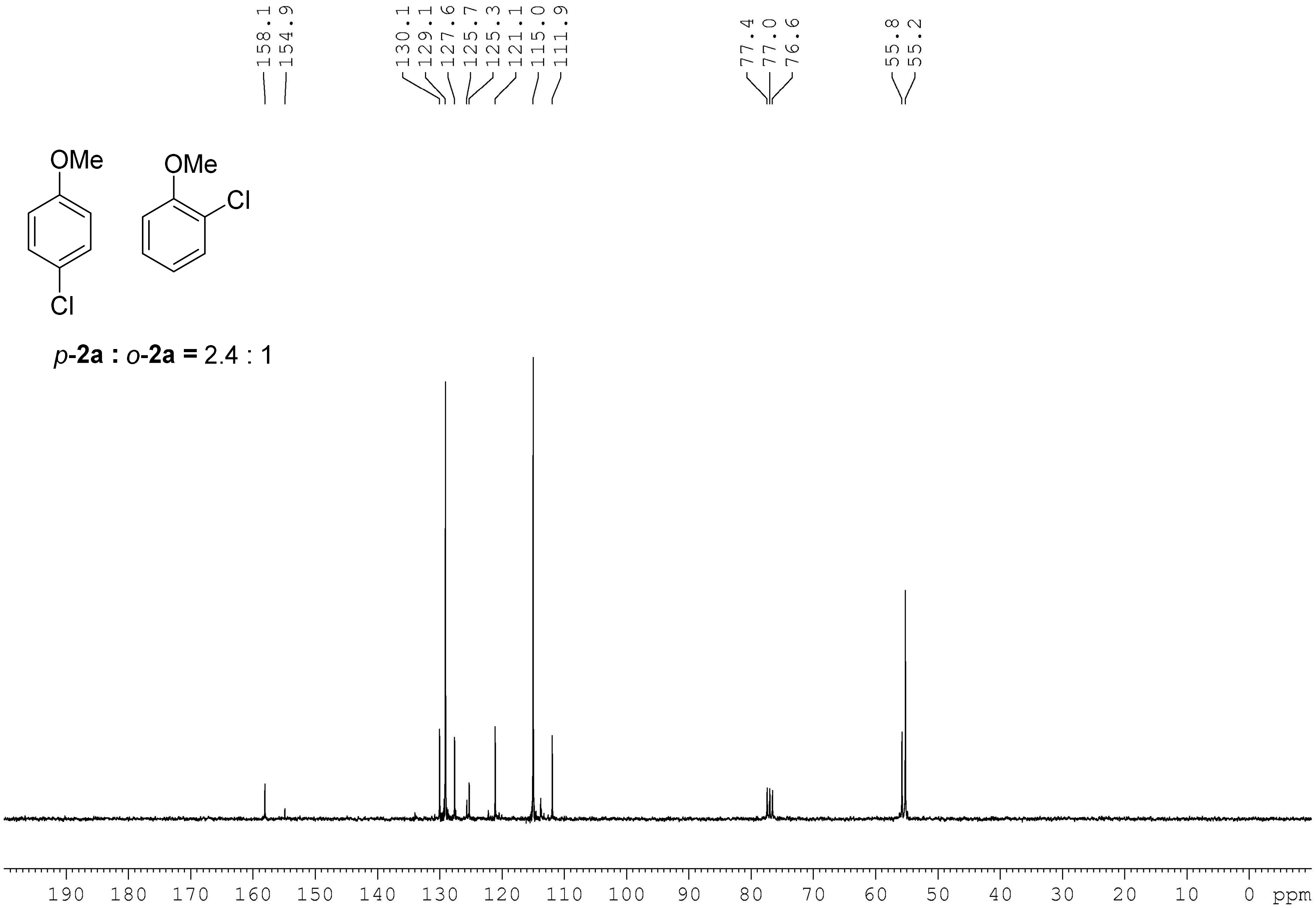

$^{13}C\{^{1}H\}$ NMR of *p*-**2a** and *o*-**2a** (75 MHz, $CDCl_3$)

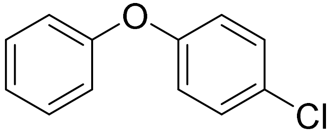

2b

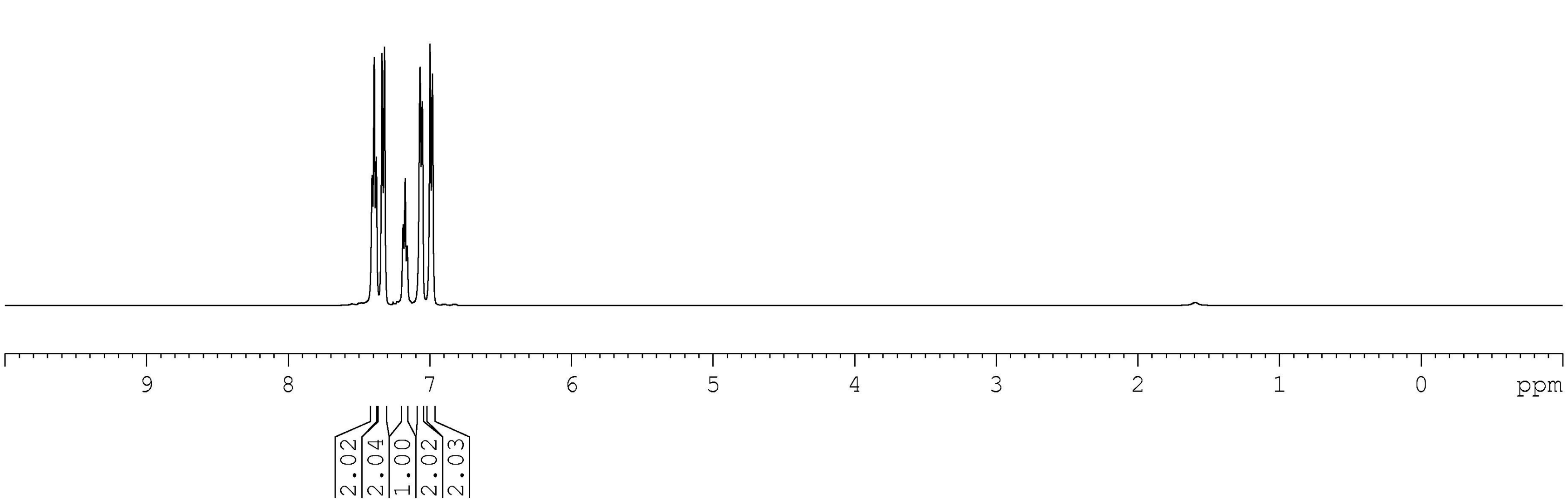

^{1}H NMR of compound **2b** (500 MHz, $CDCl_3$)

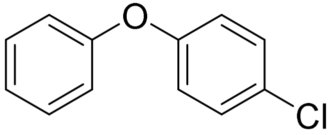

2b

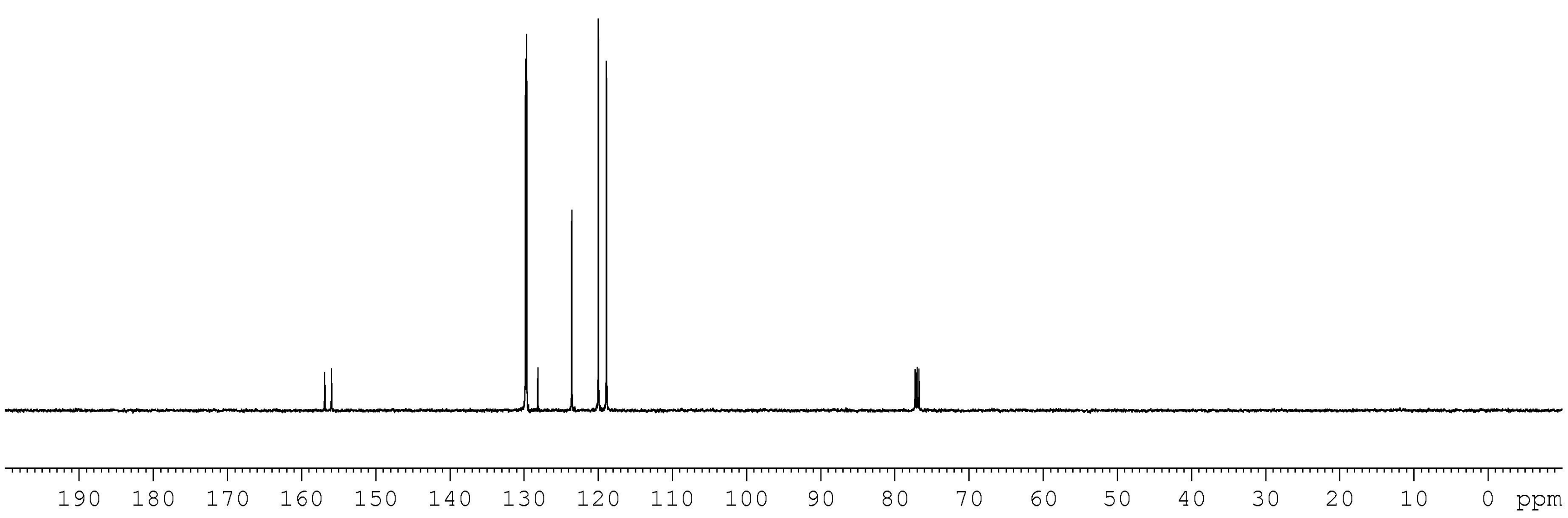

$^{13}C\{^{1}H\}$ NMR of compound **2b** (126 MHz, $CDCl_3$)

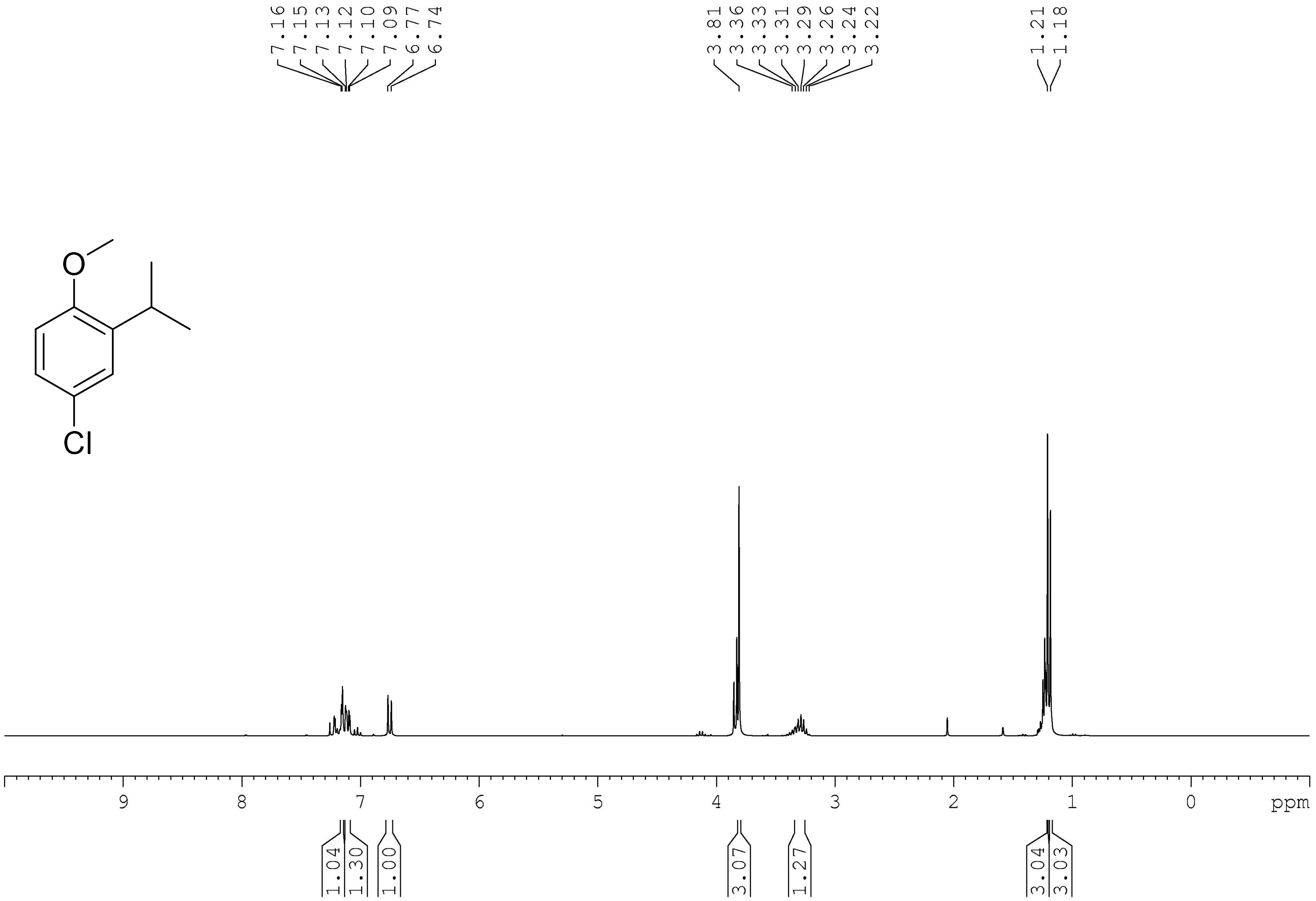

^{1}H NMR of compound **2c** (300 MHz, $CDCl_3$)

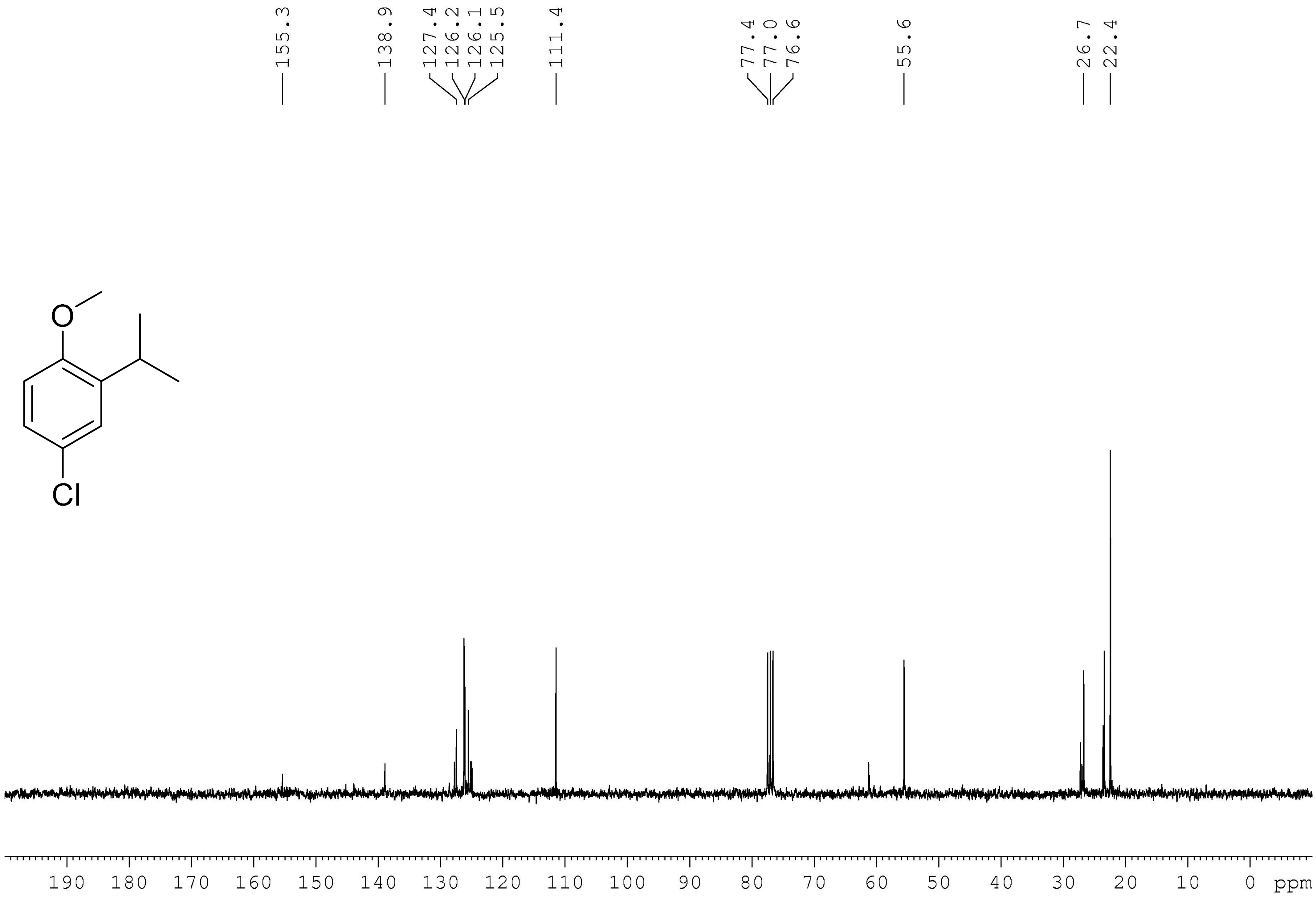

$^{13}C\{^1H\}$ NMR of compound **2c** (75 MHz, $CDCl_3$)

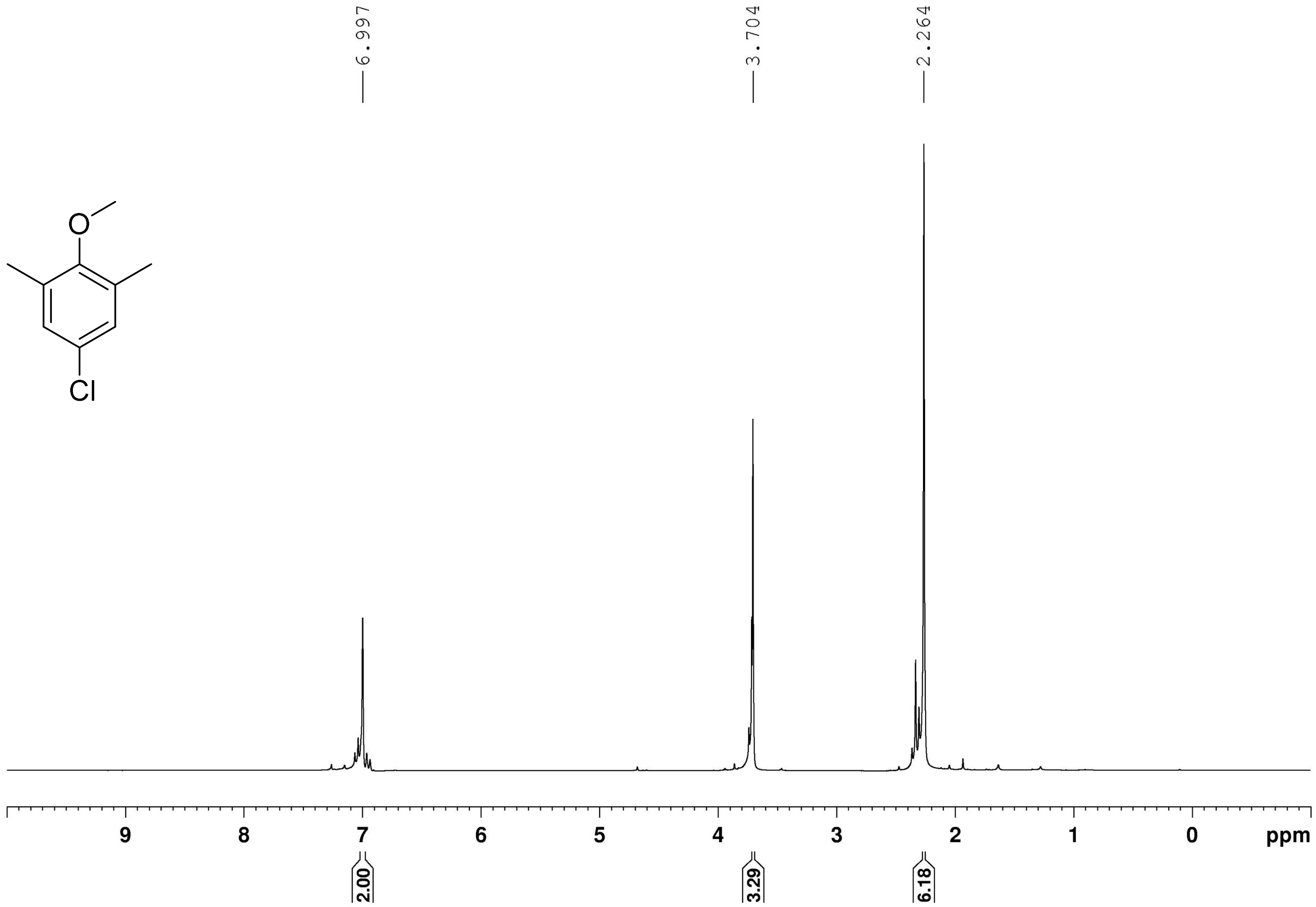

^{1}H NMR of compound **2d** (300 MHz, $CDCl_3$)

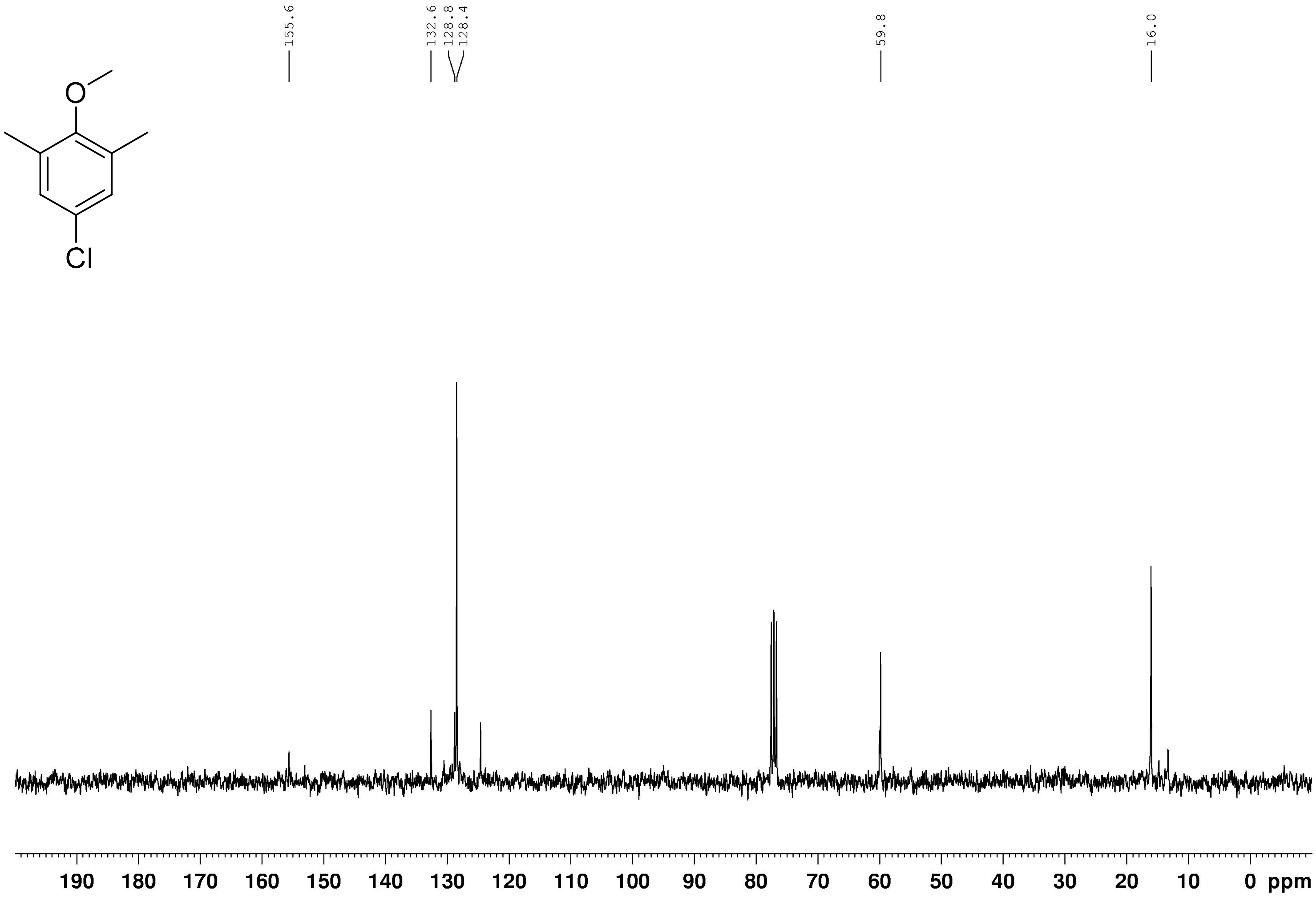

$^{13}C\{^{1}H\}$ NMR of compound **2d** (75 MHz, $CDCl_3$)

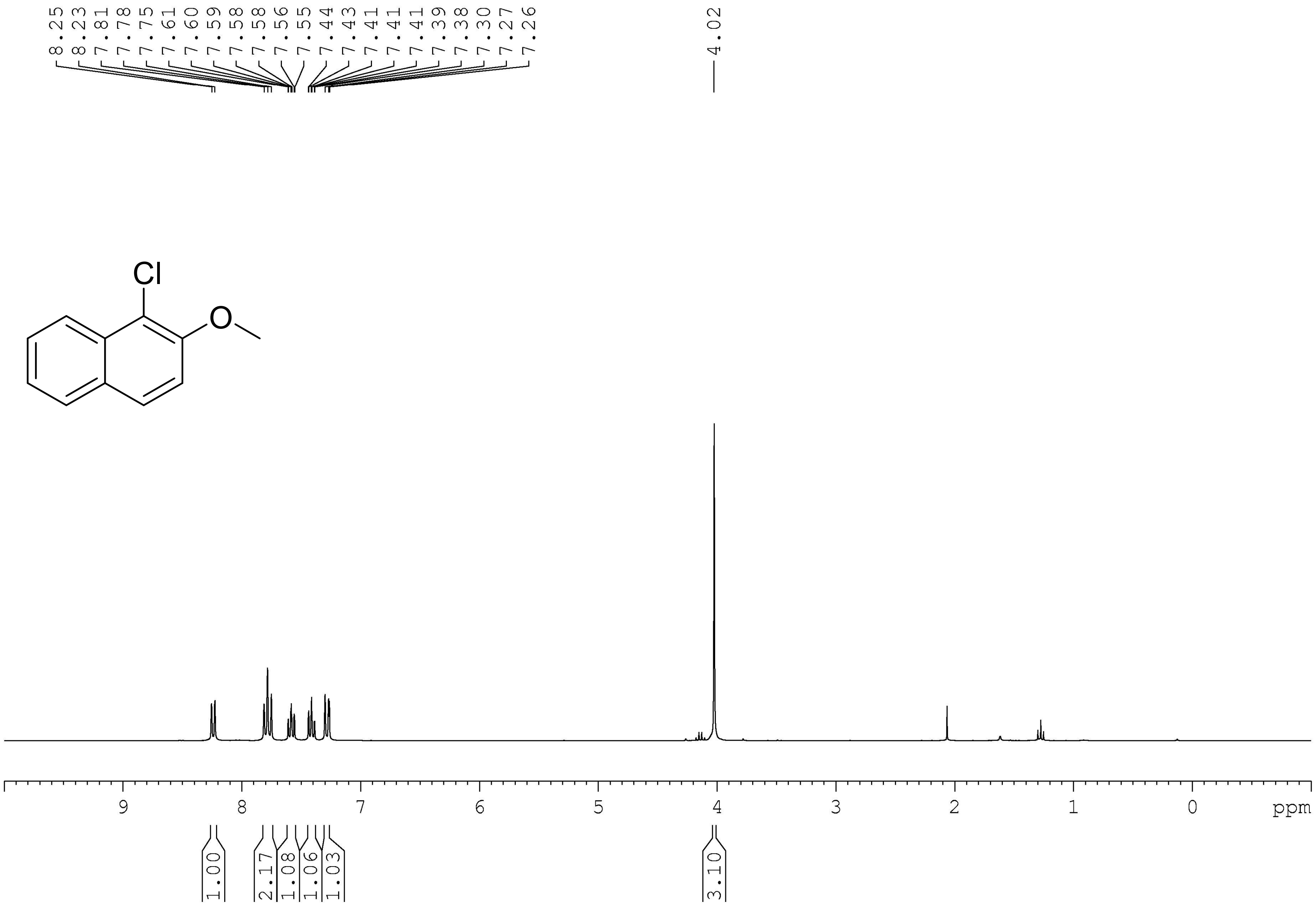

^{1}H NMR of compound **2e** (300 MHz, $CDCl_3$)

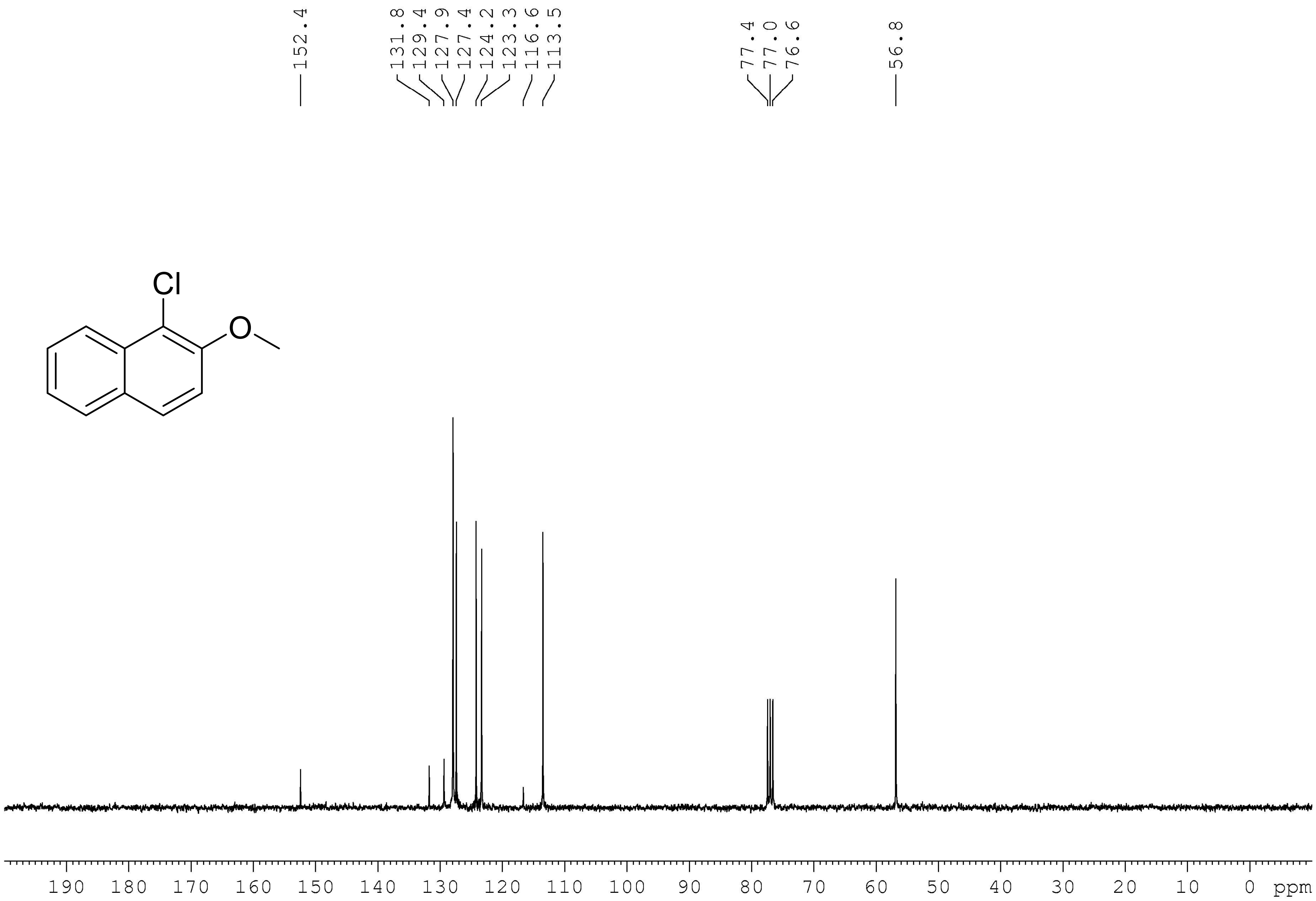

$^{13}C\{^1H\}$ NMR of compound **2e** (75 MHz, $CDCl_3$)

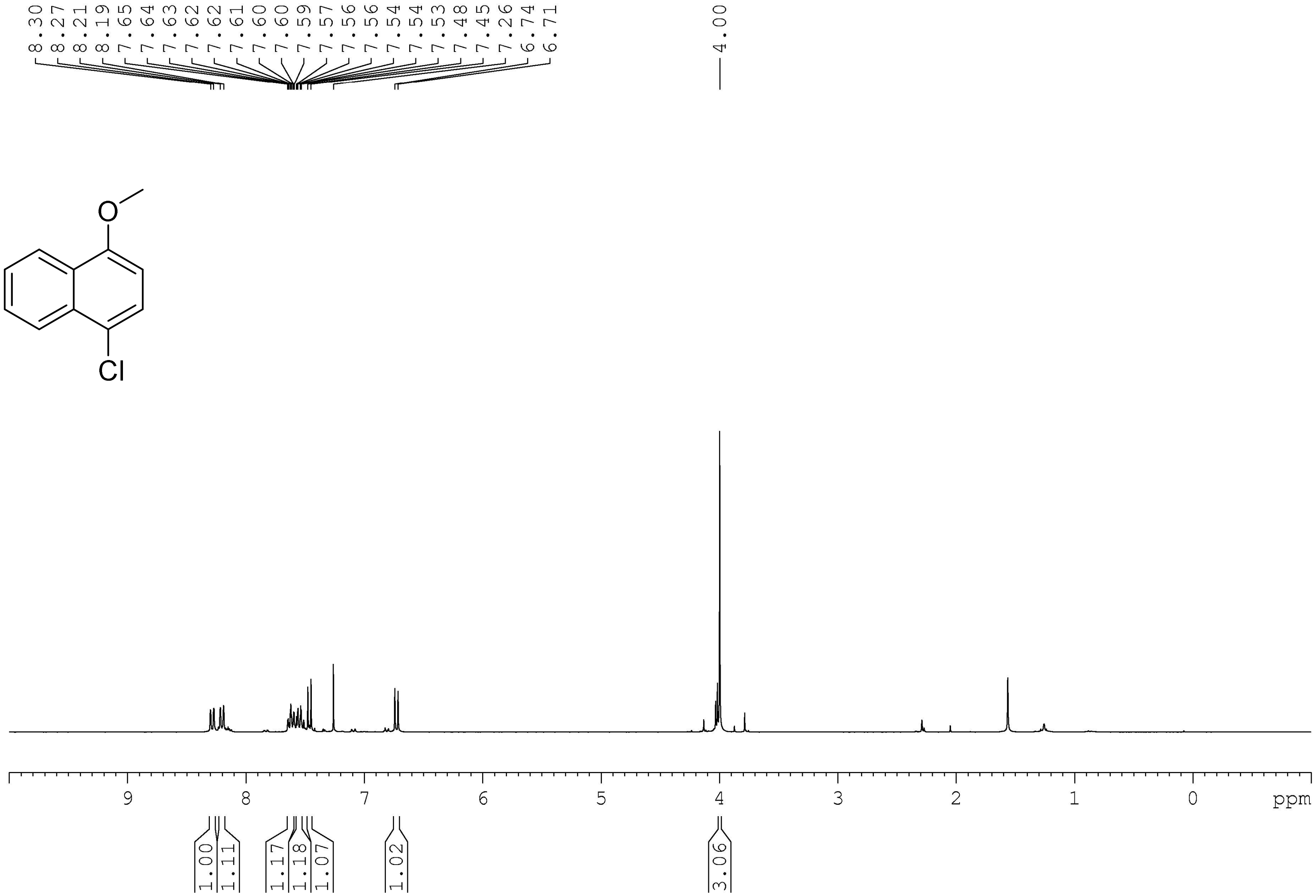

^{1}H NMR of compound **2f** (300 MHz, $CDCl_3$)

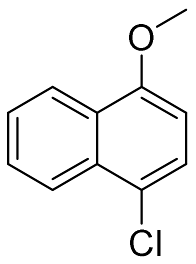

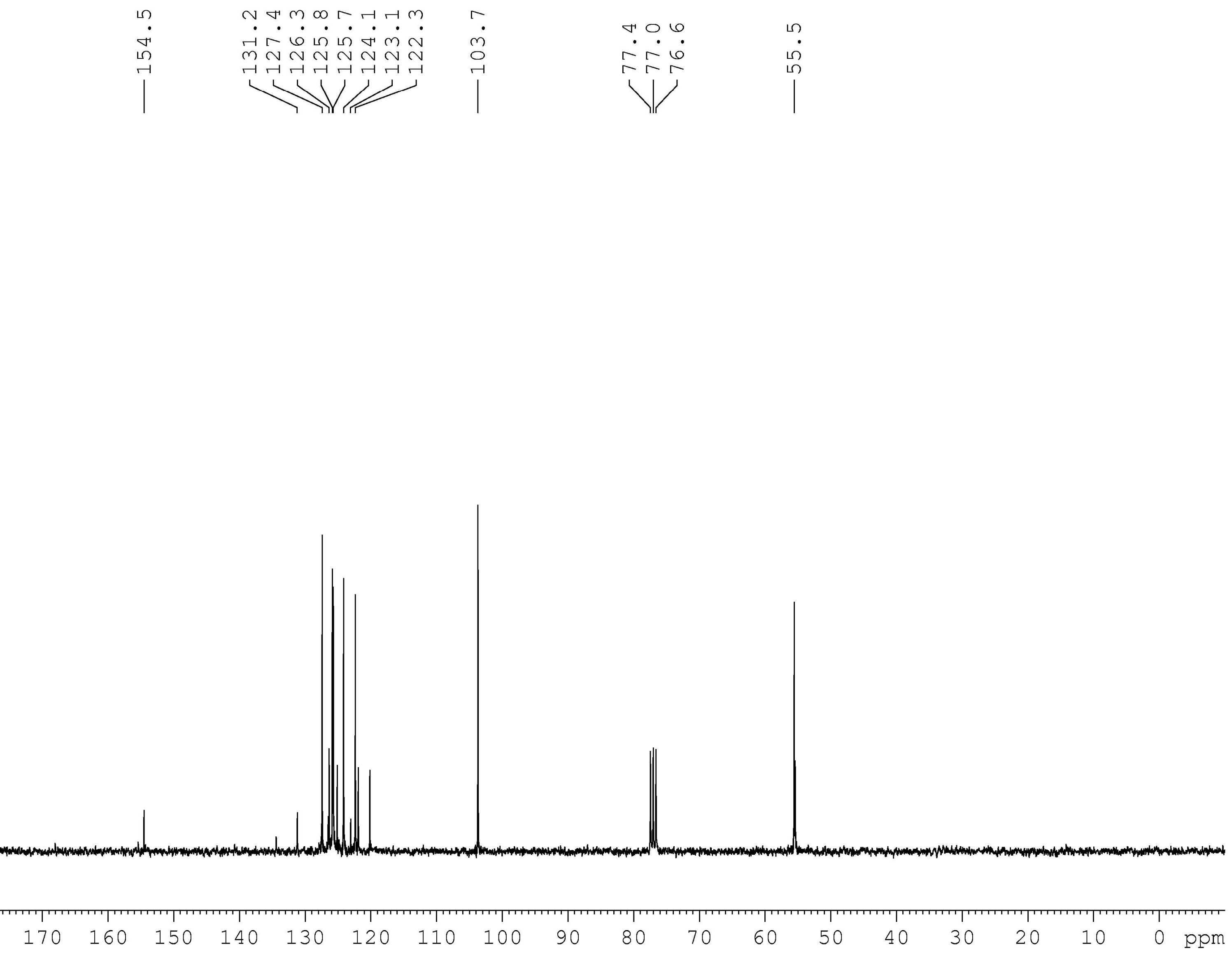

$^{13}C\{^{1}H\}$ NMR of compound **2f** (75 MHz, $CDCl_3$)

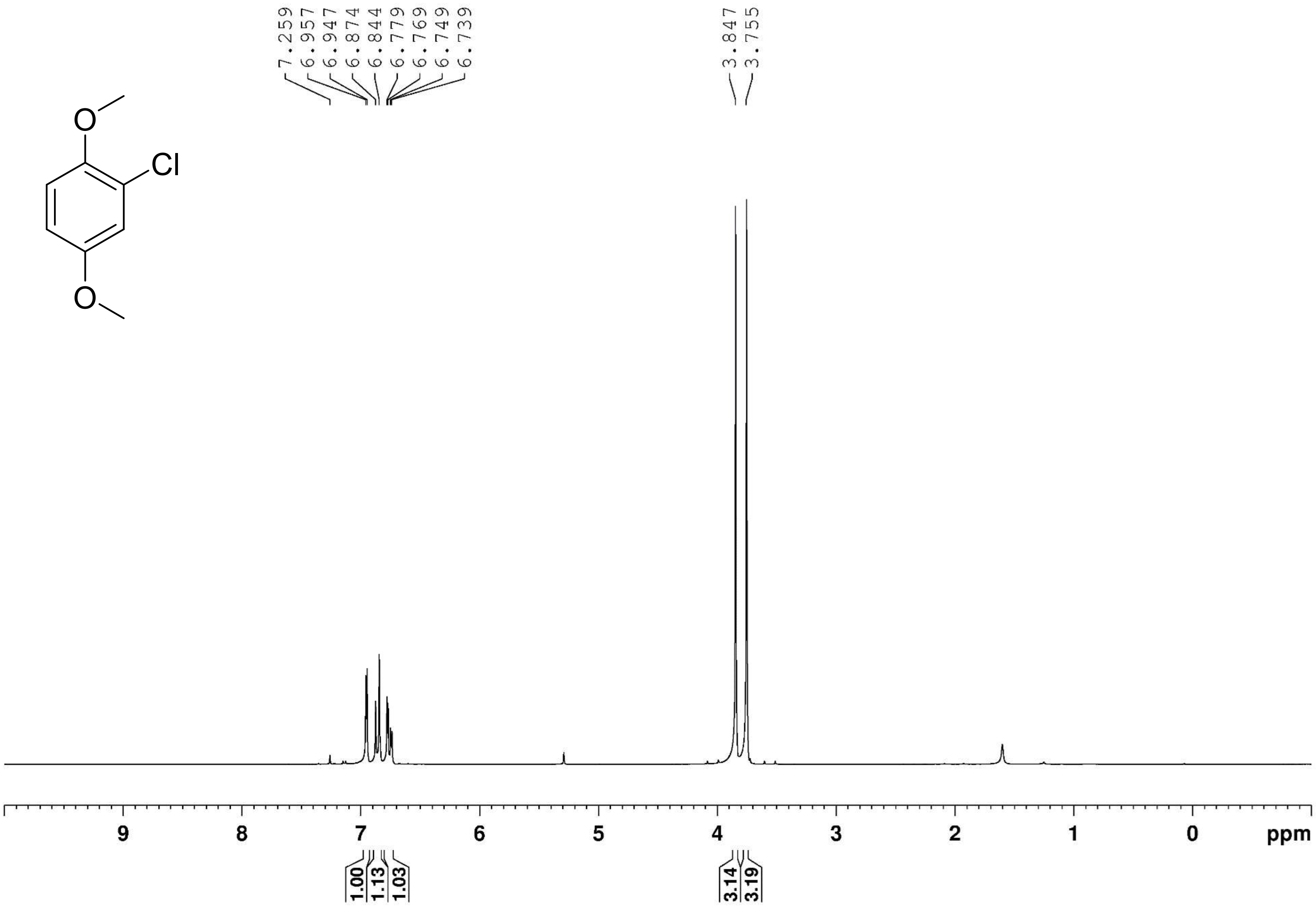

^{1}H NMR of compound **2g** (300 MHz, $CDCl_3$)

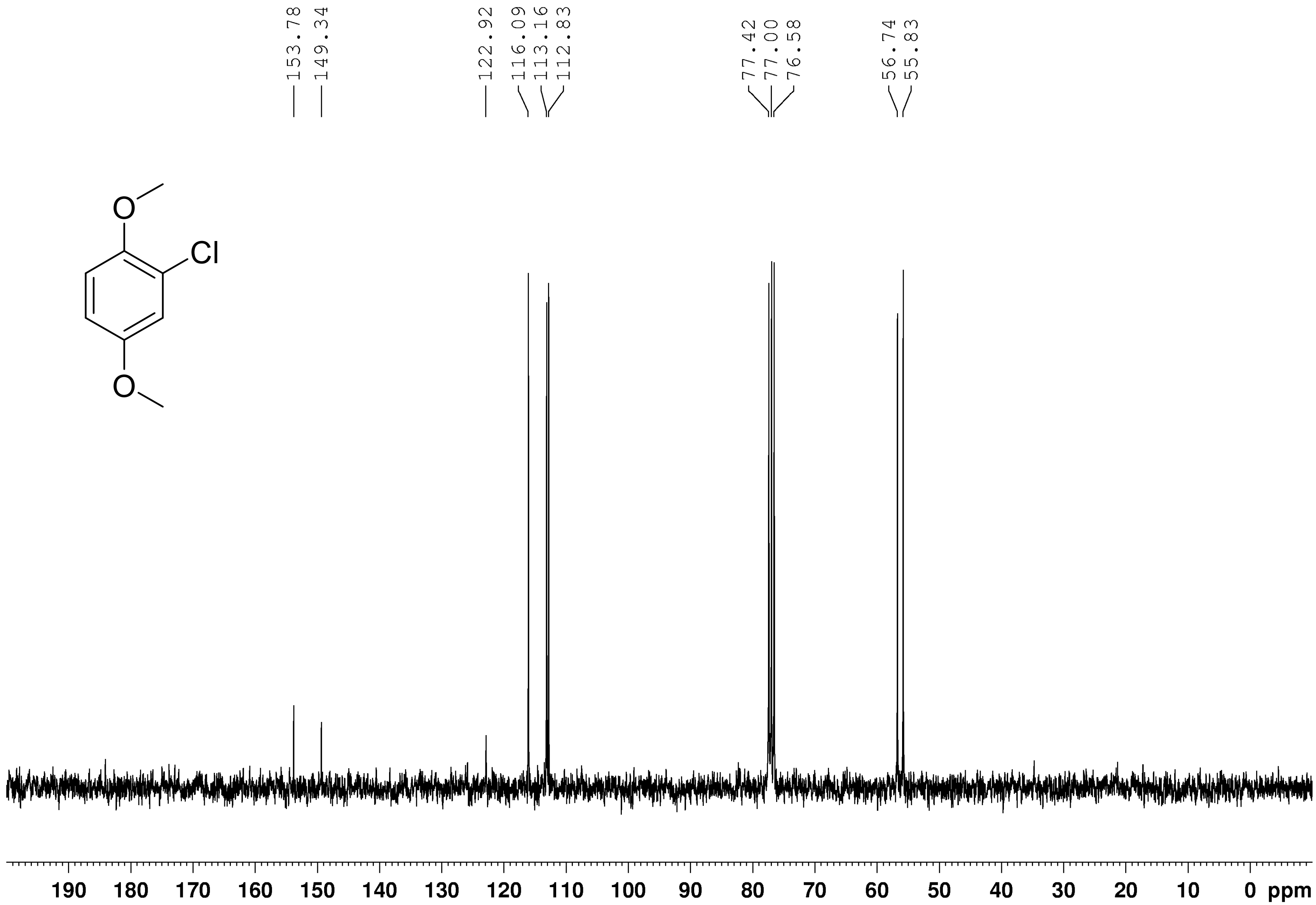

$^{13}C\{^{1}H\}$ NMR of compound **2g** (75 MHz, $CDCl_3$)

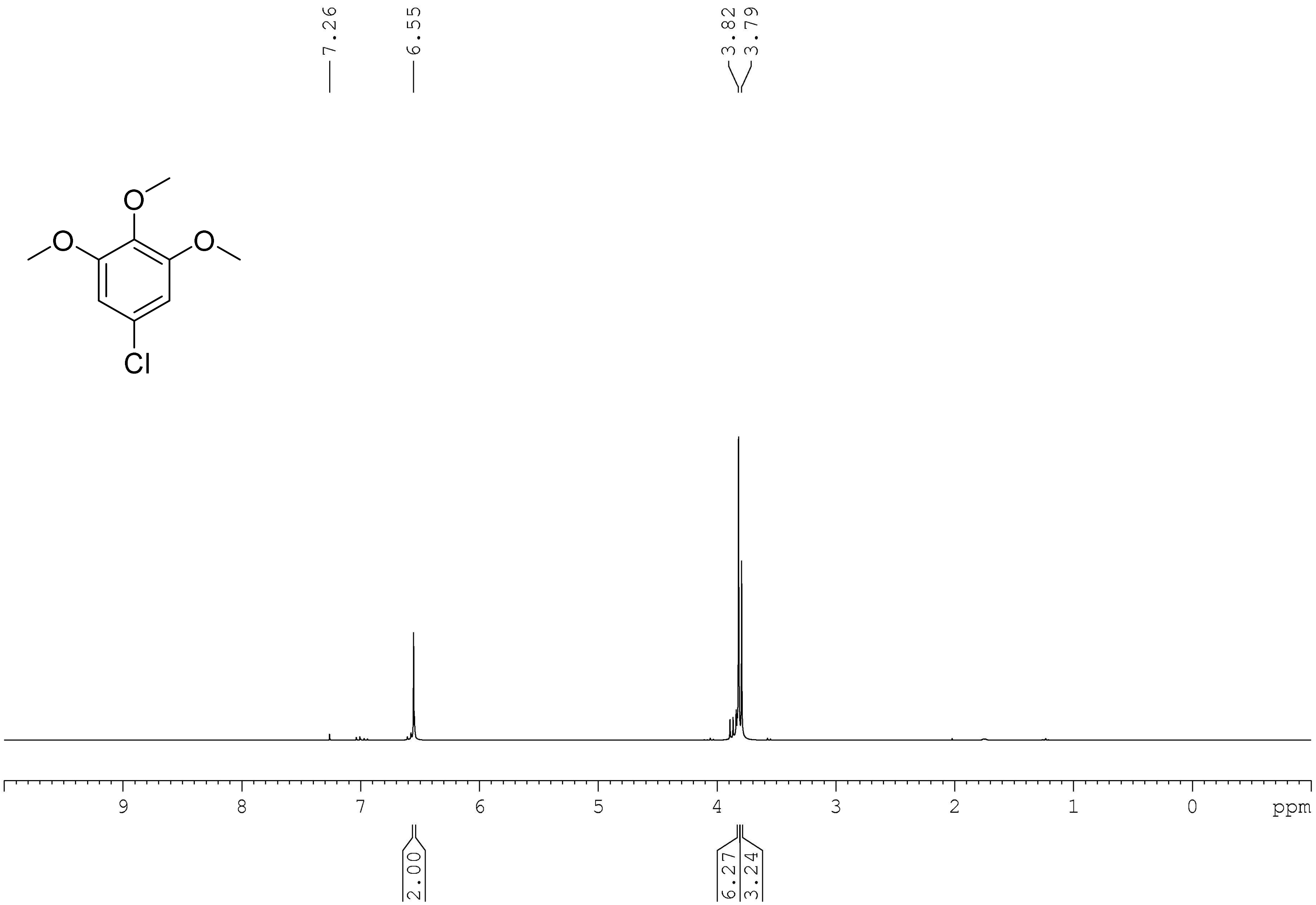

^{1}H NMR of compound **2h** (300 MHz, $CDCl_3$)

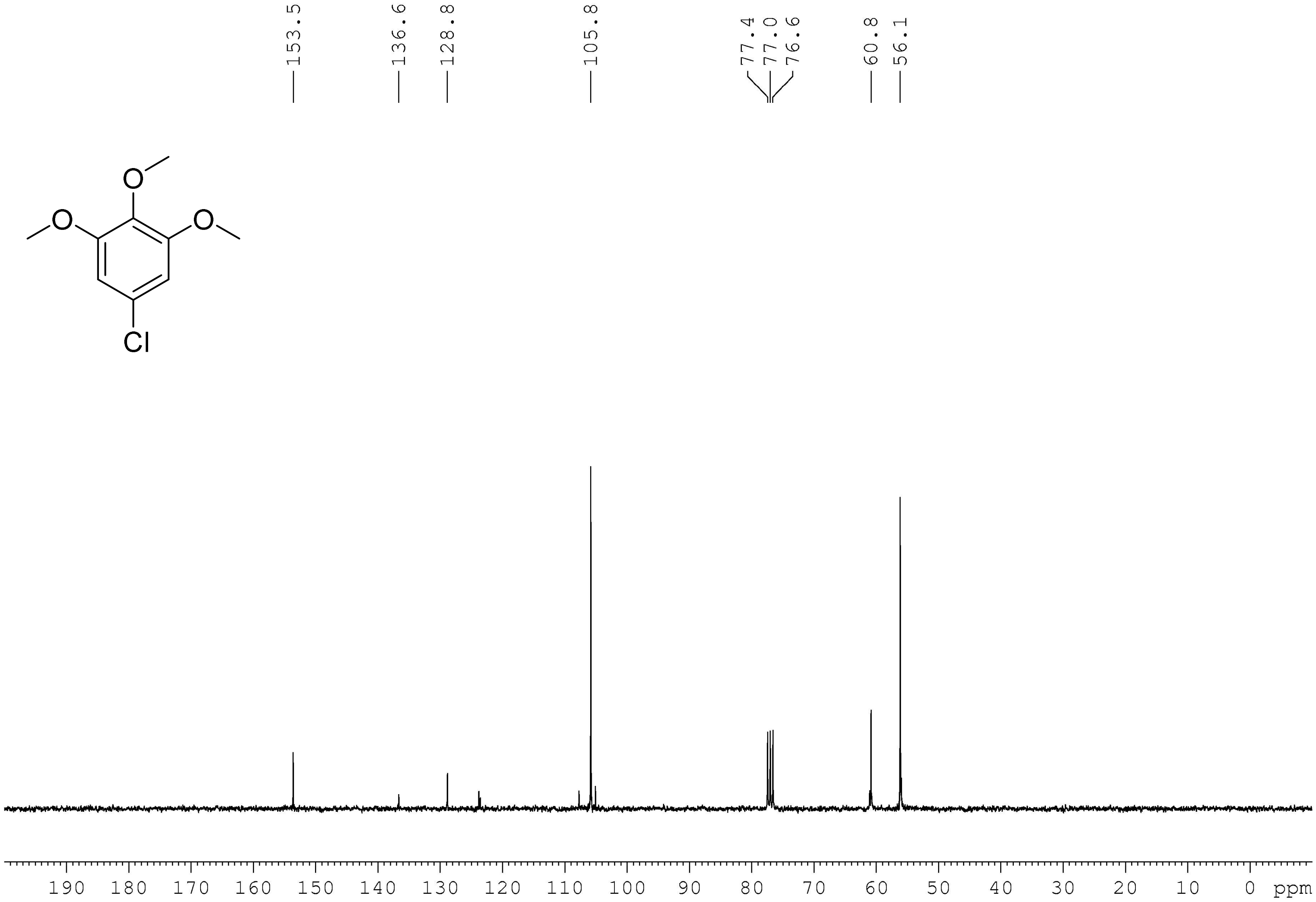

$^{13}C\{^{1}H\}$ NMR of compound **2h** (75 MHz, $CDCl_3$)

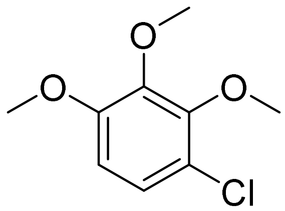

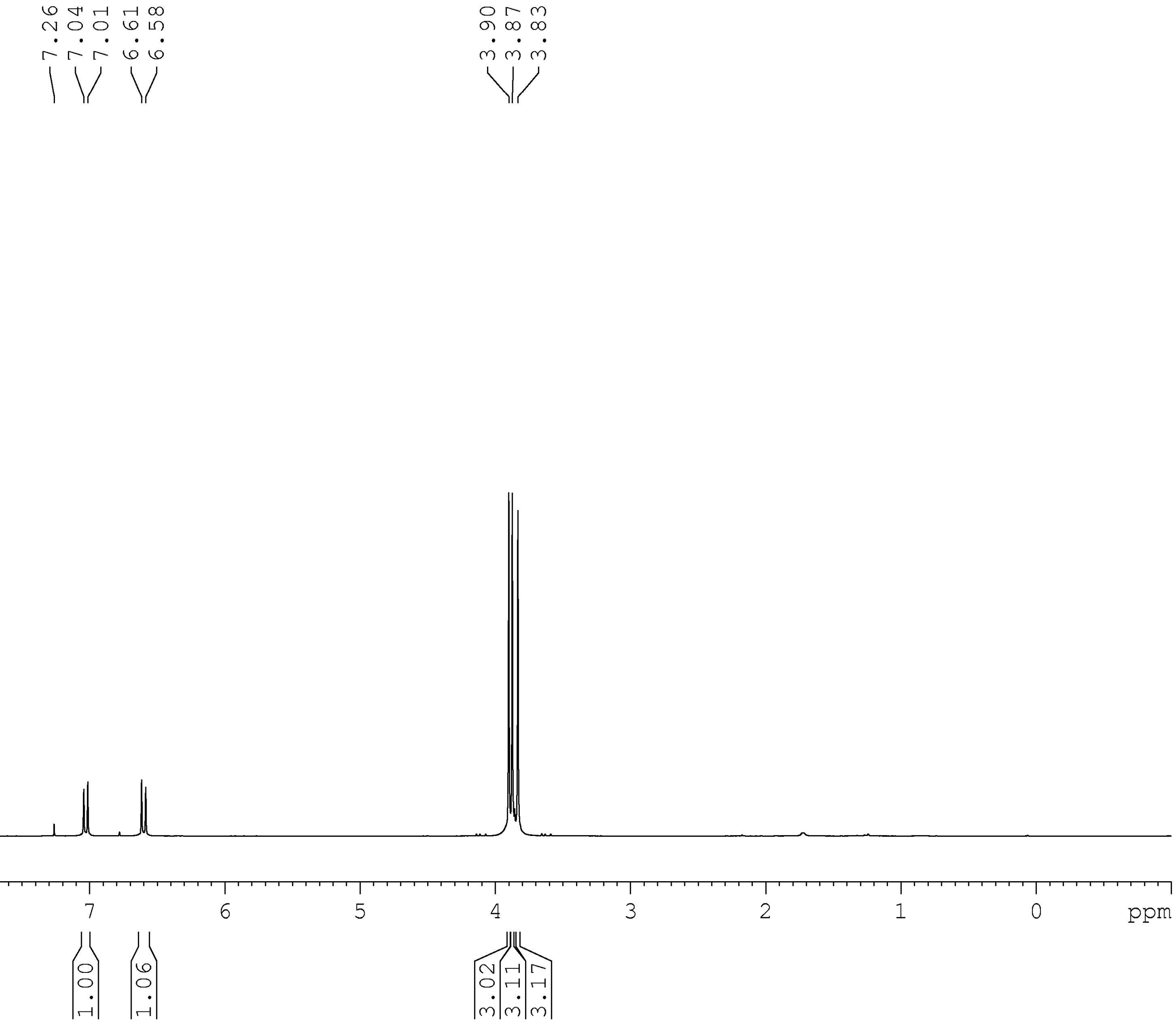

^{1}H NMR of compound **2h'** (300 MHz, $CDCl_3$)

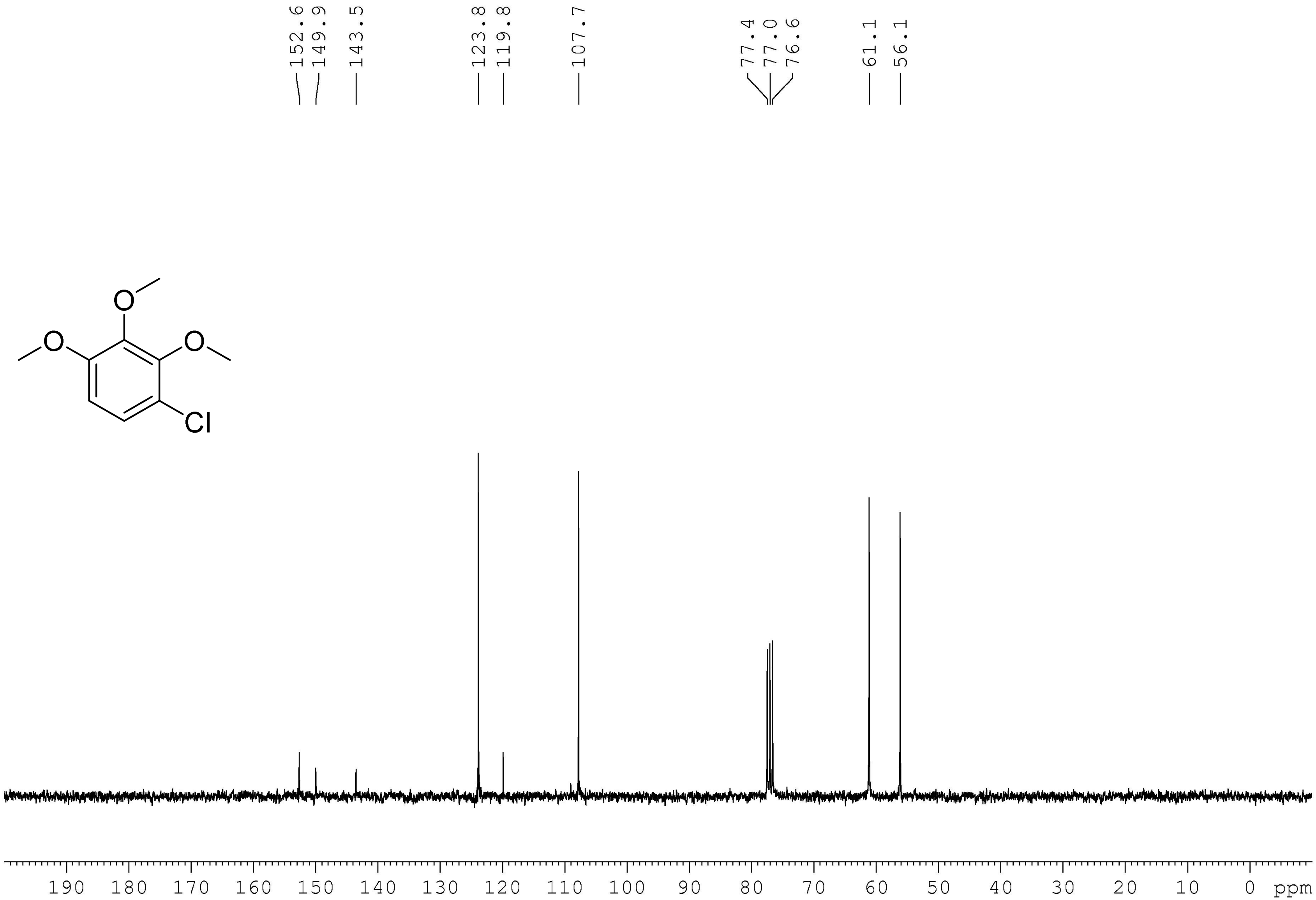

$^{13}C\{^{1}H\}$ NMR of compound **2h'** (75 MHz, $CDCl_3$)

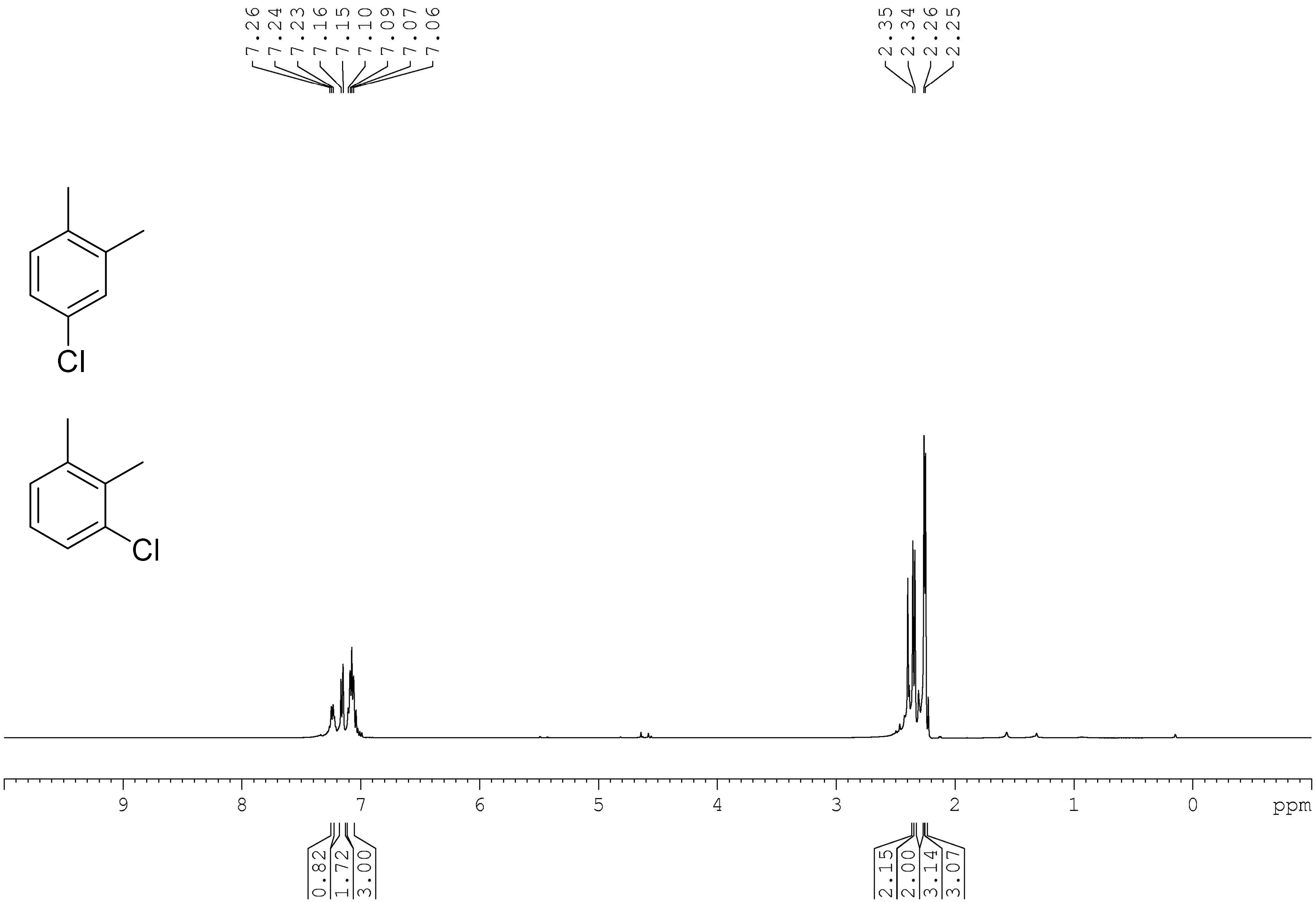

^{1}H NMR of compounds **2i** and **2i'** (500 MHz, $CDCl_3$)

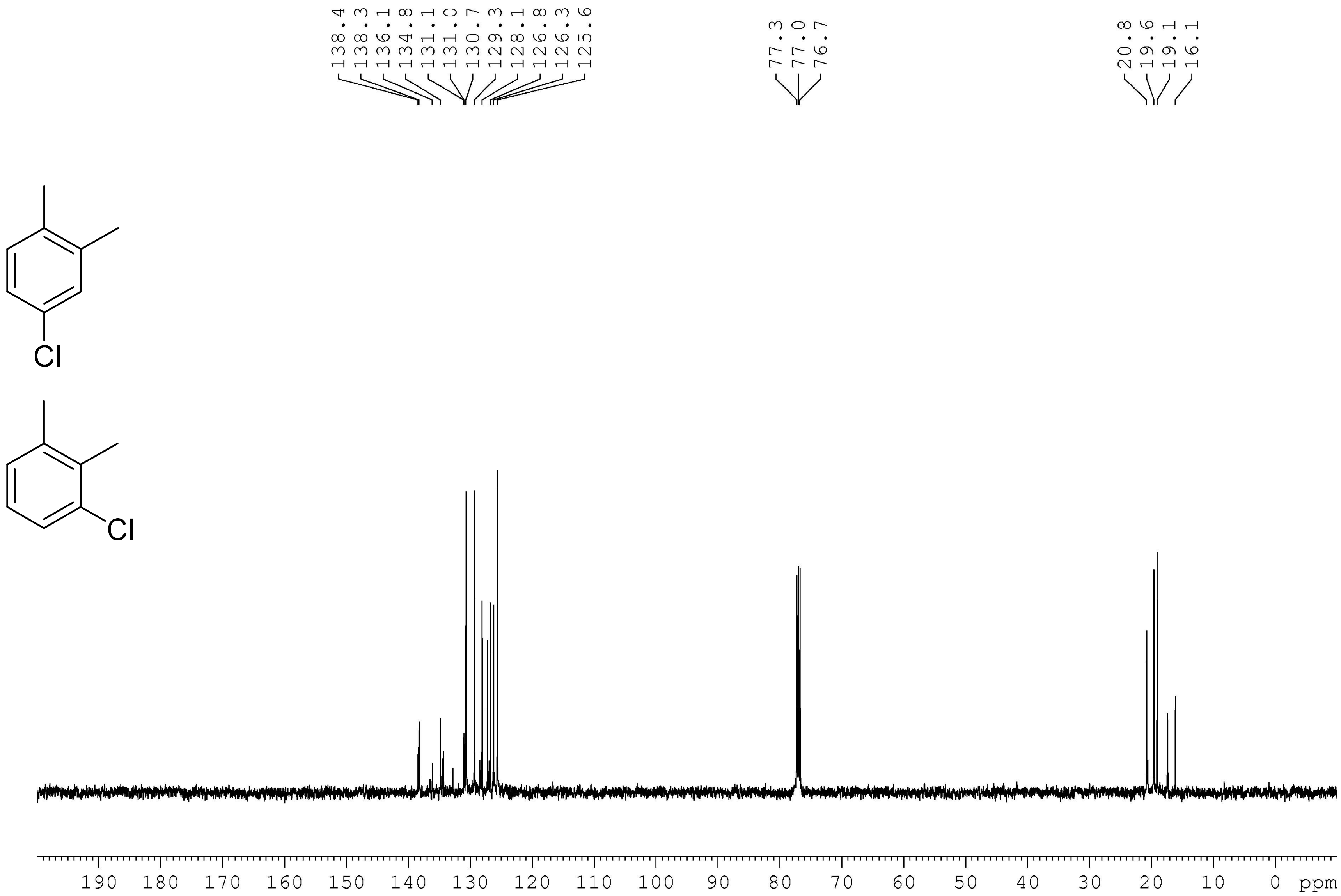

$^{13}C\{^{1}H\}$ NMR of compounds **2i** and **2i'** (126 MHz, $CDCl_3$)

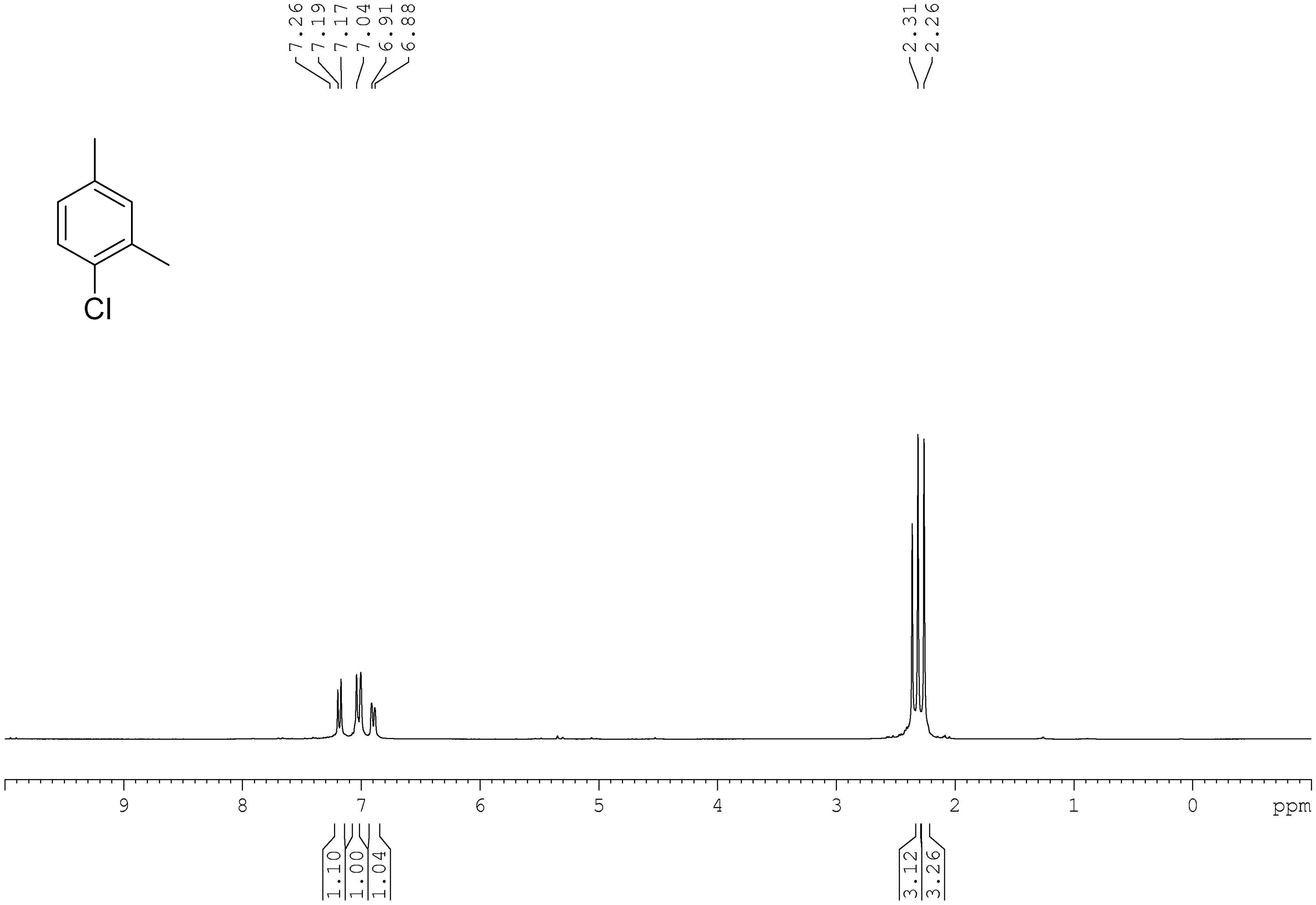

^{1}H NMR of compound **2j** (300 MHz, $CDCl_3$)

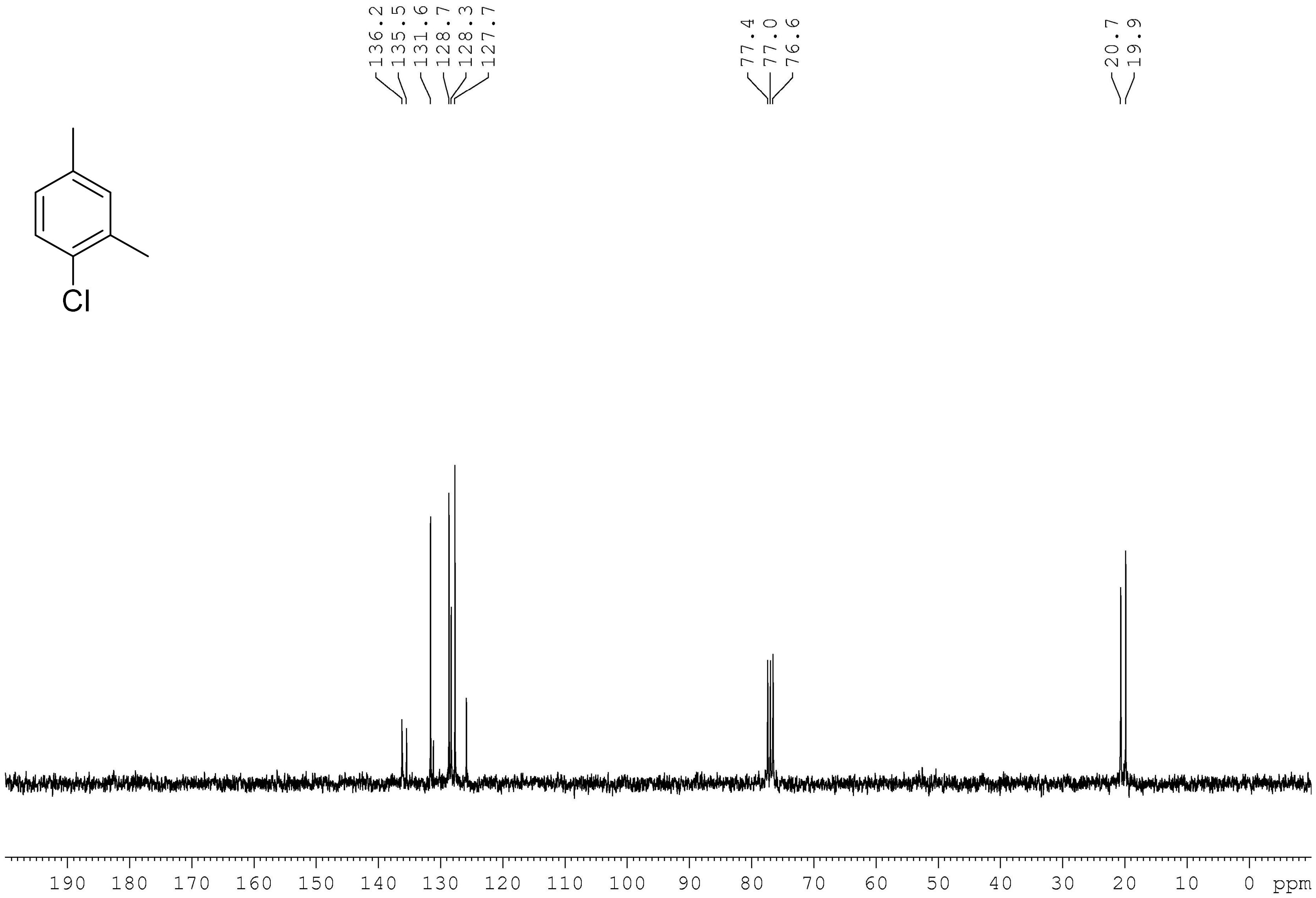

$^{13}C\{^{1}H\}$ NMR of compound **2j** (75 MHz, $CDCl_3$)

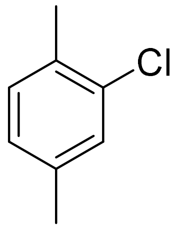

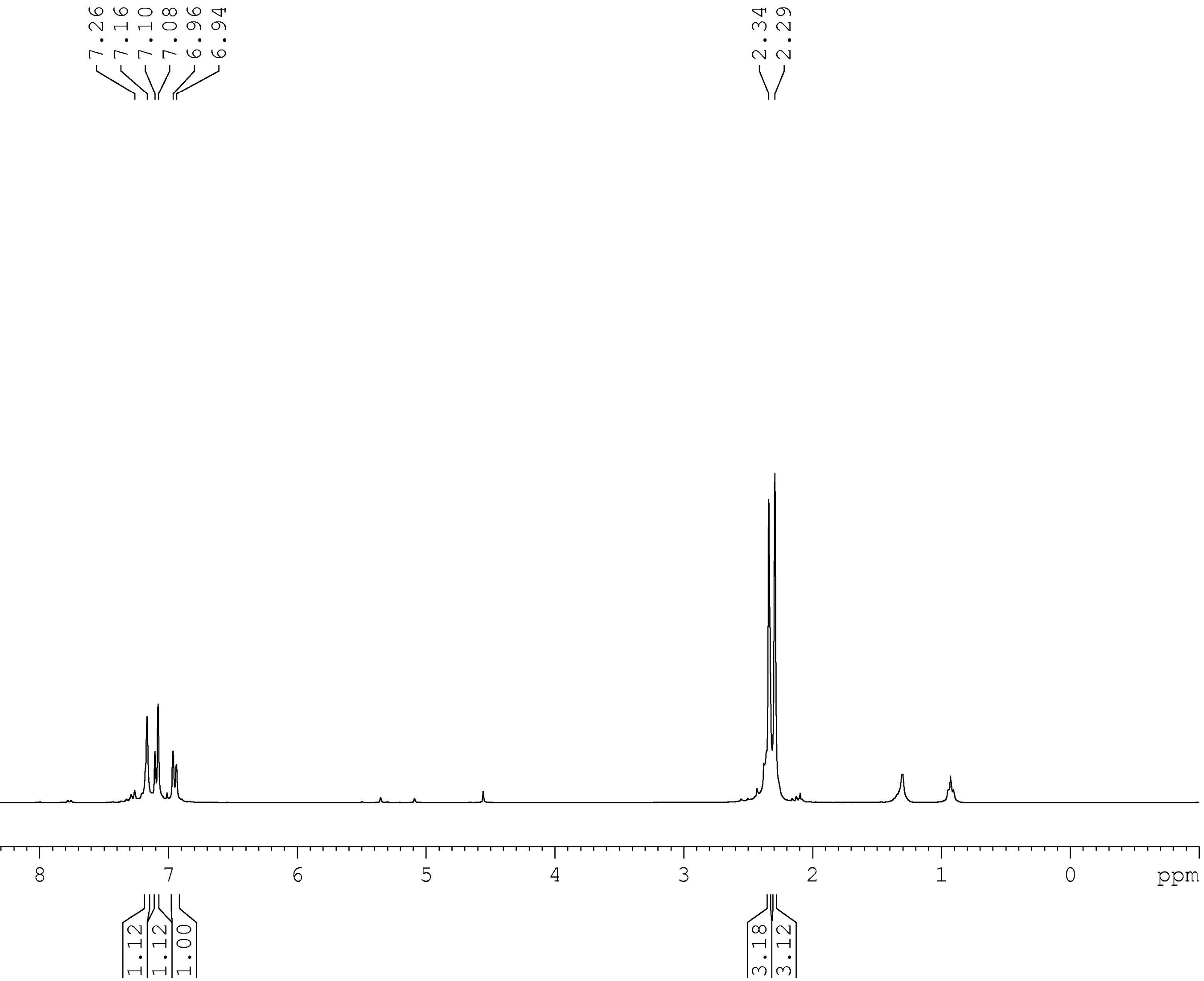

^{1}H NMR of compound **2k** (300 MHz, $CDCl_3$)

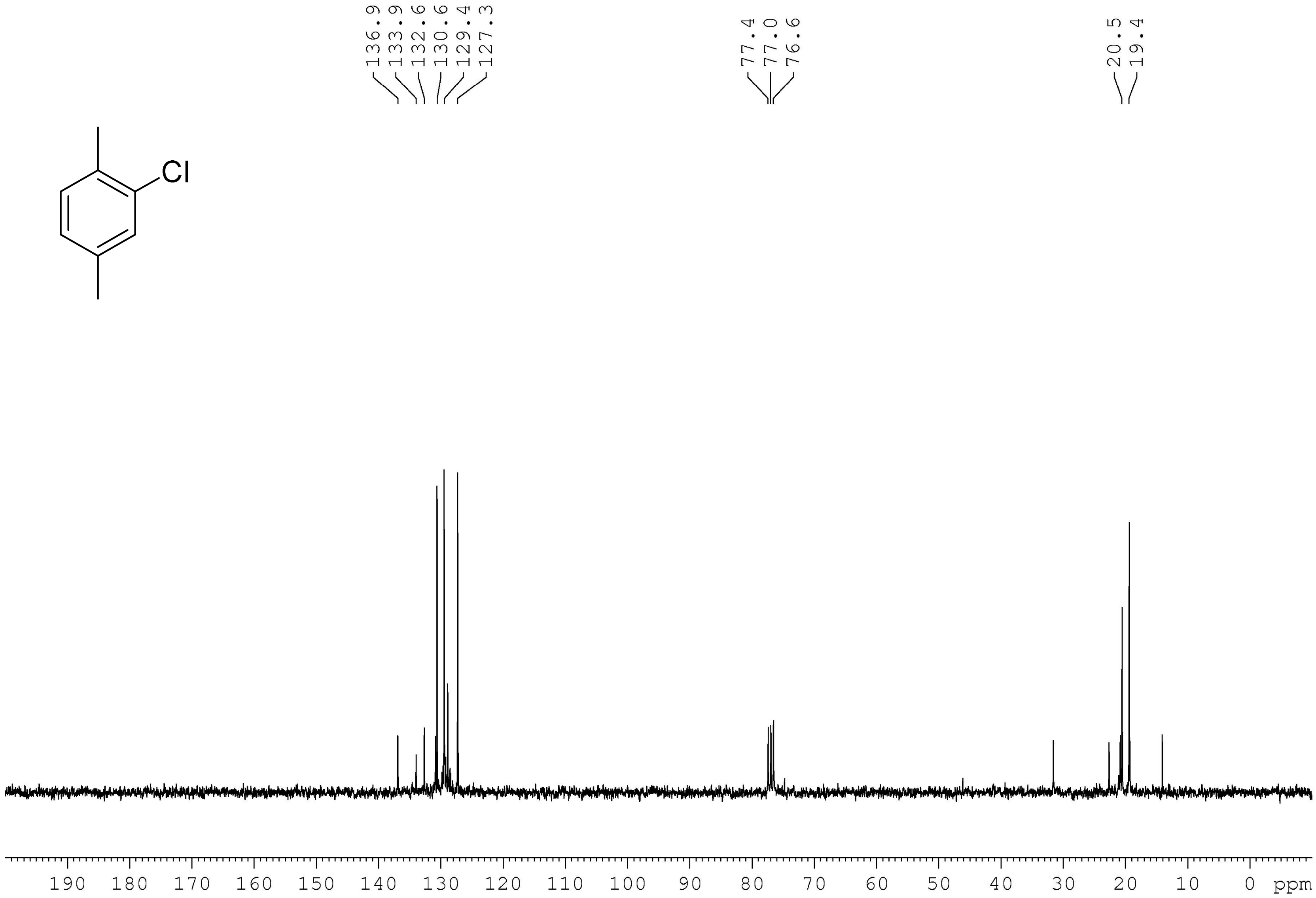

$^{13}C\{^1H\}$ NMR of compound **2k** (75 MHz, $CDCl_3$)

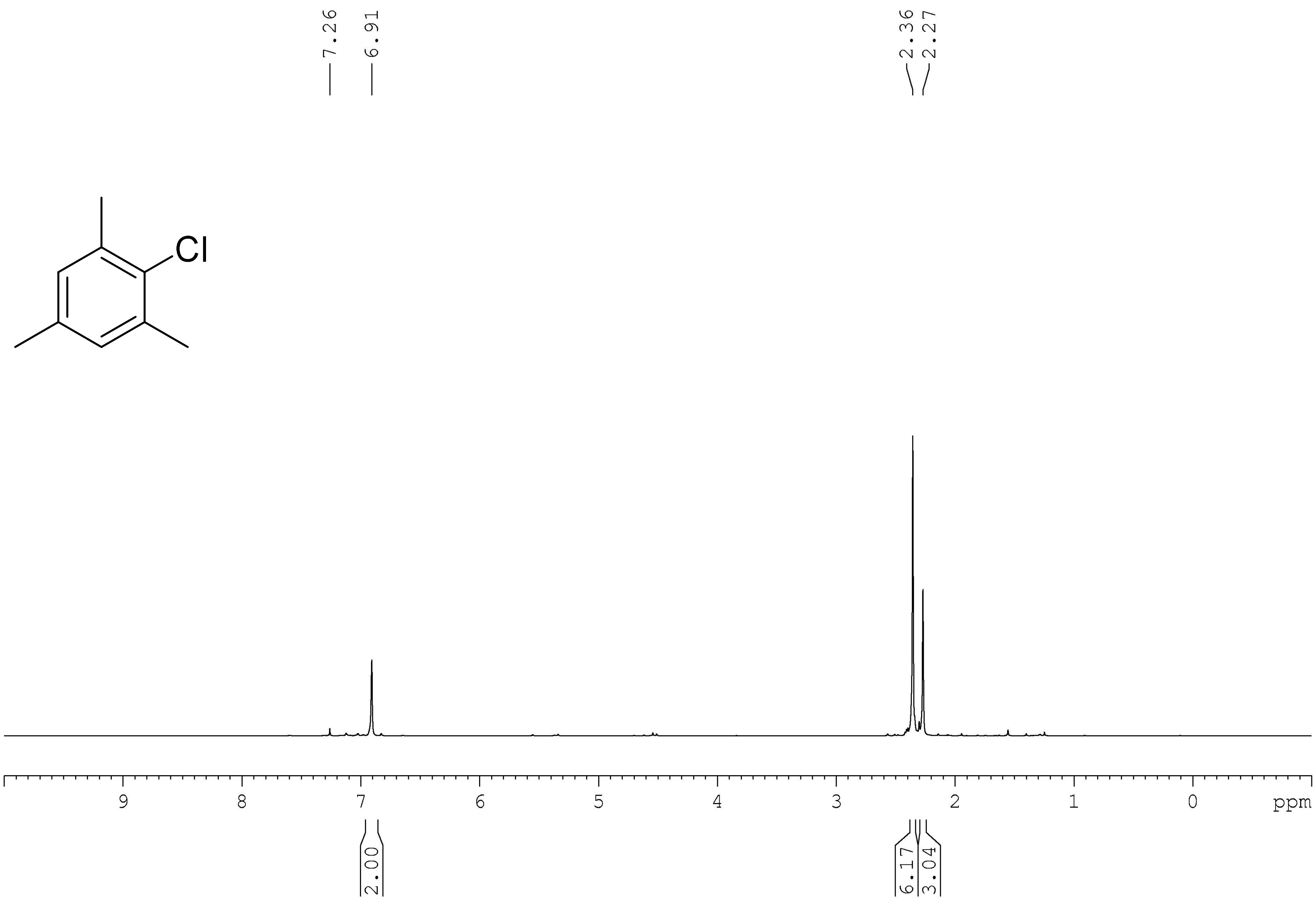

^{1}H NMR of compound **2l** (300 MHz, $CDCl_3$)

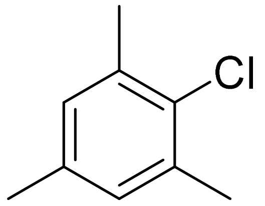

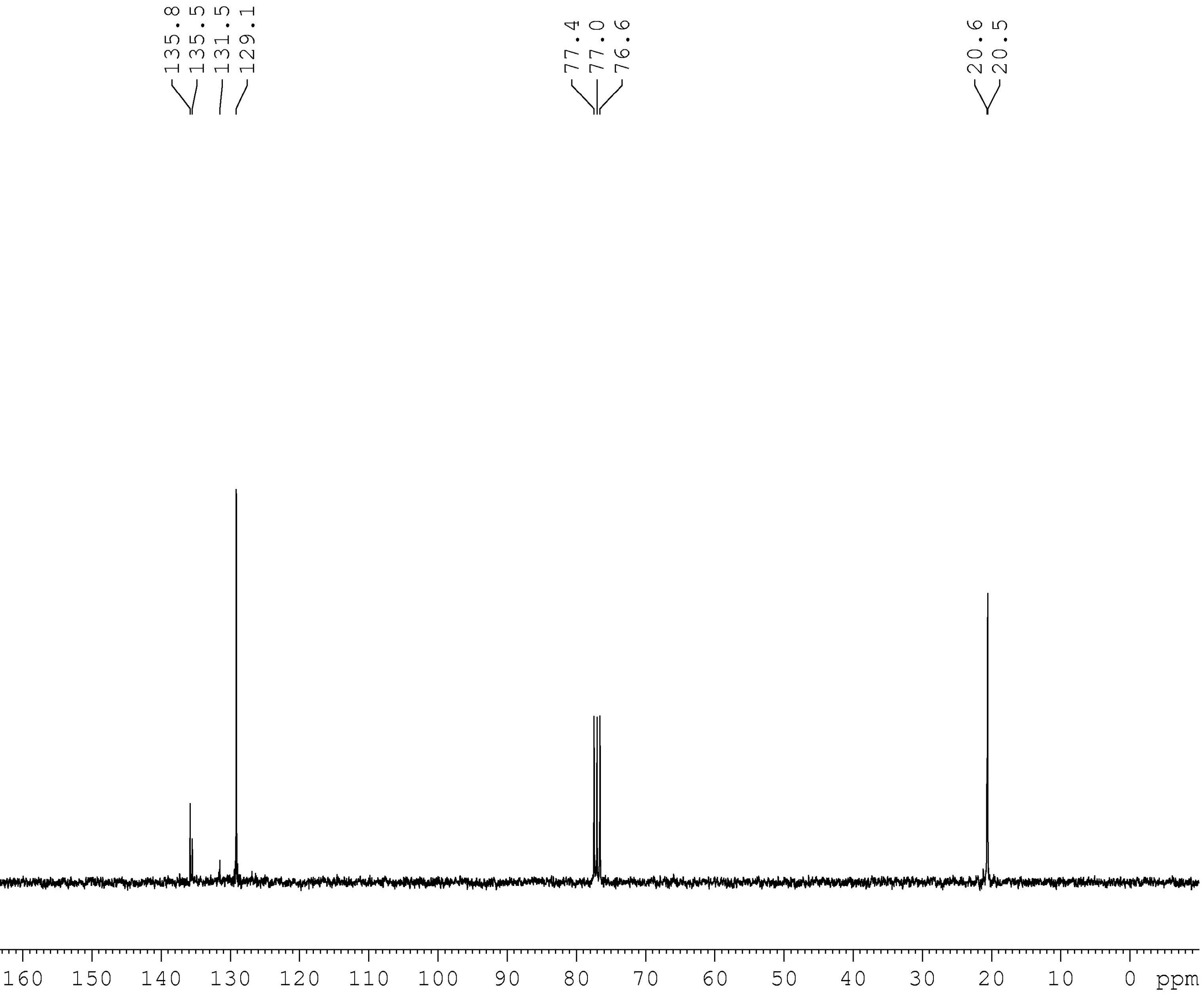

$^{13}C\{^{1}H\}$ NMR of compound **2l** (75 MHz, $CDCl_3$)

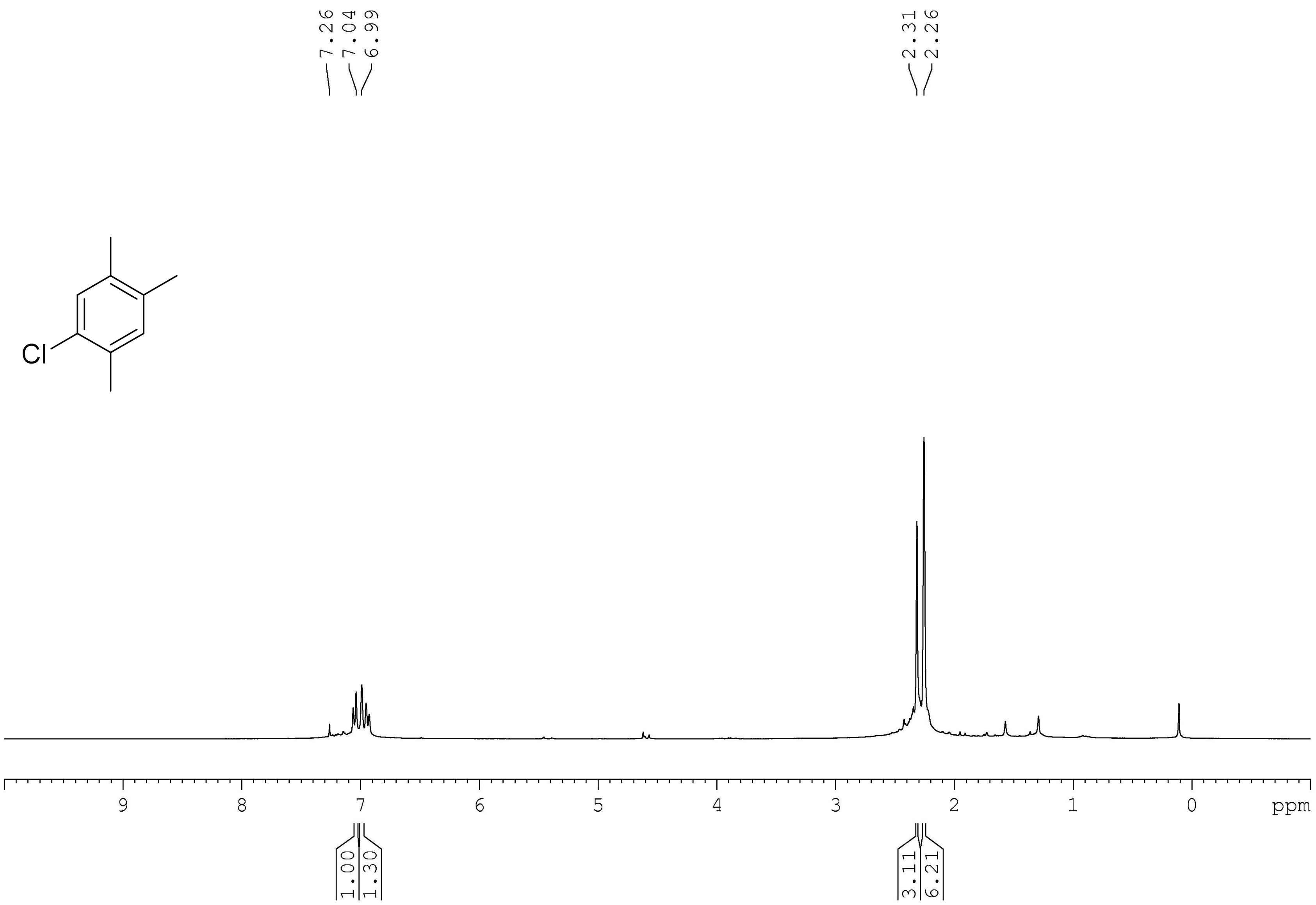

^{1}H NMR of compound **2m** (300 MHz, $CDCl_3$)

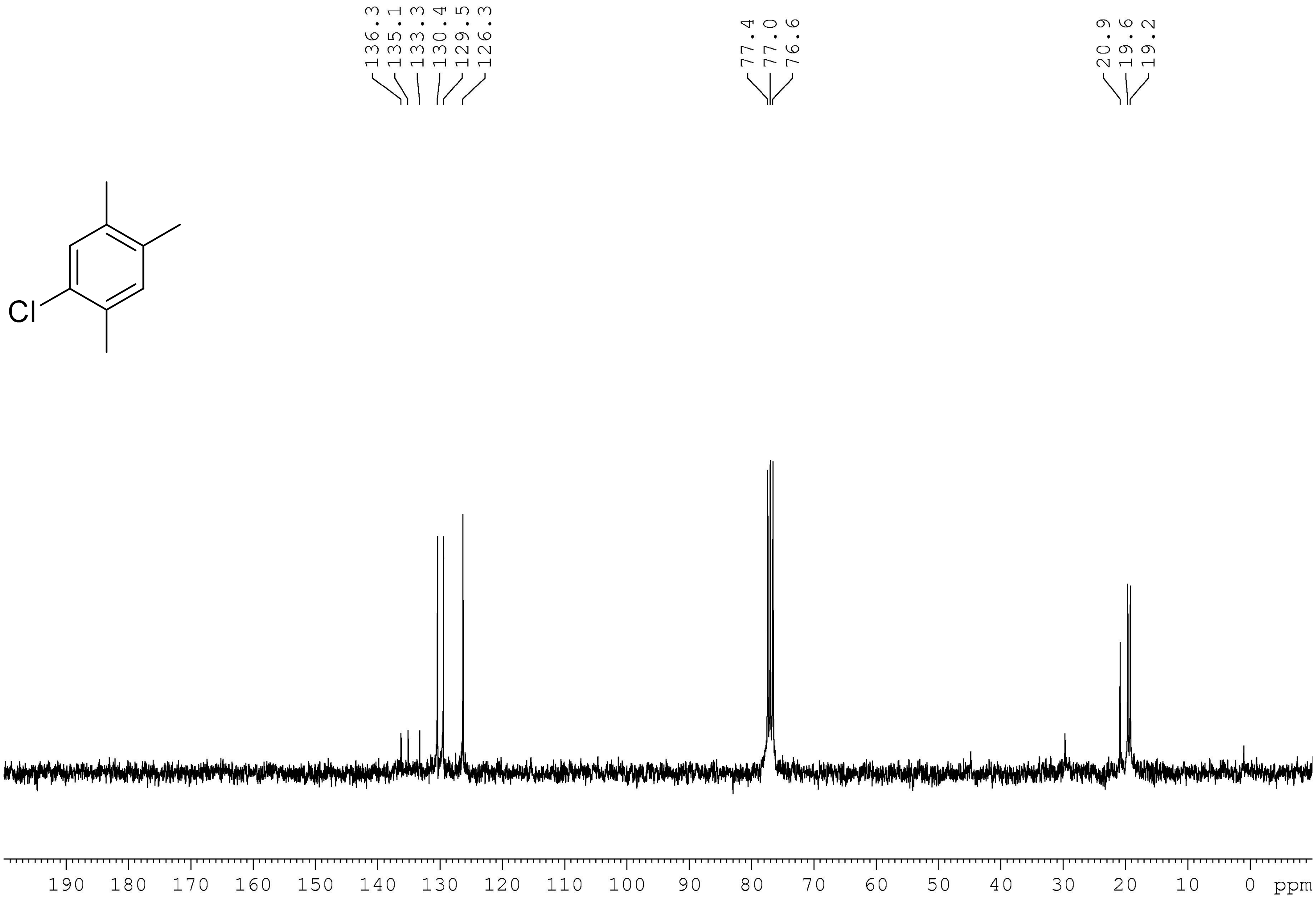

$^{13}C\{^{1}H\}$ NMR of compound **2m** (75 MHz, $CDCl_3$)

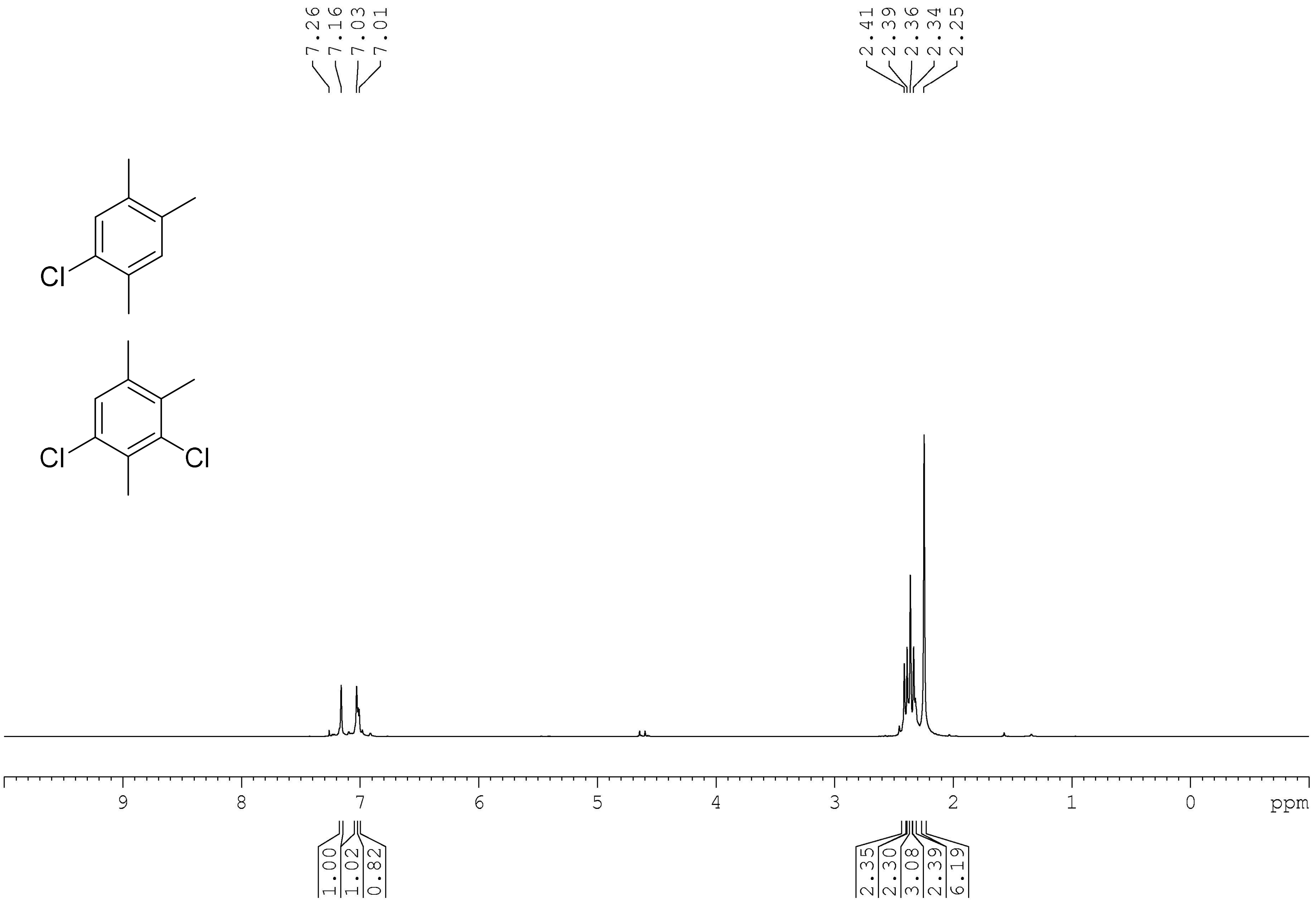

^{1}H NMR of compounds **2m** and **2m'** (300 MHz, $CDCl_3$)

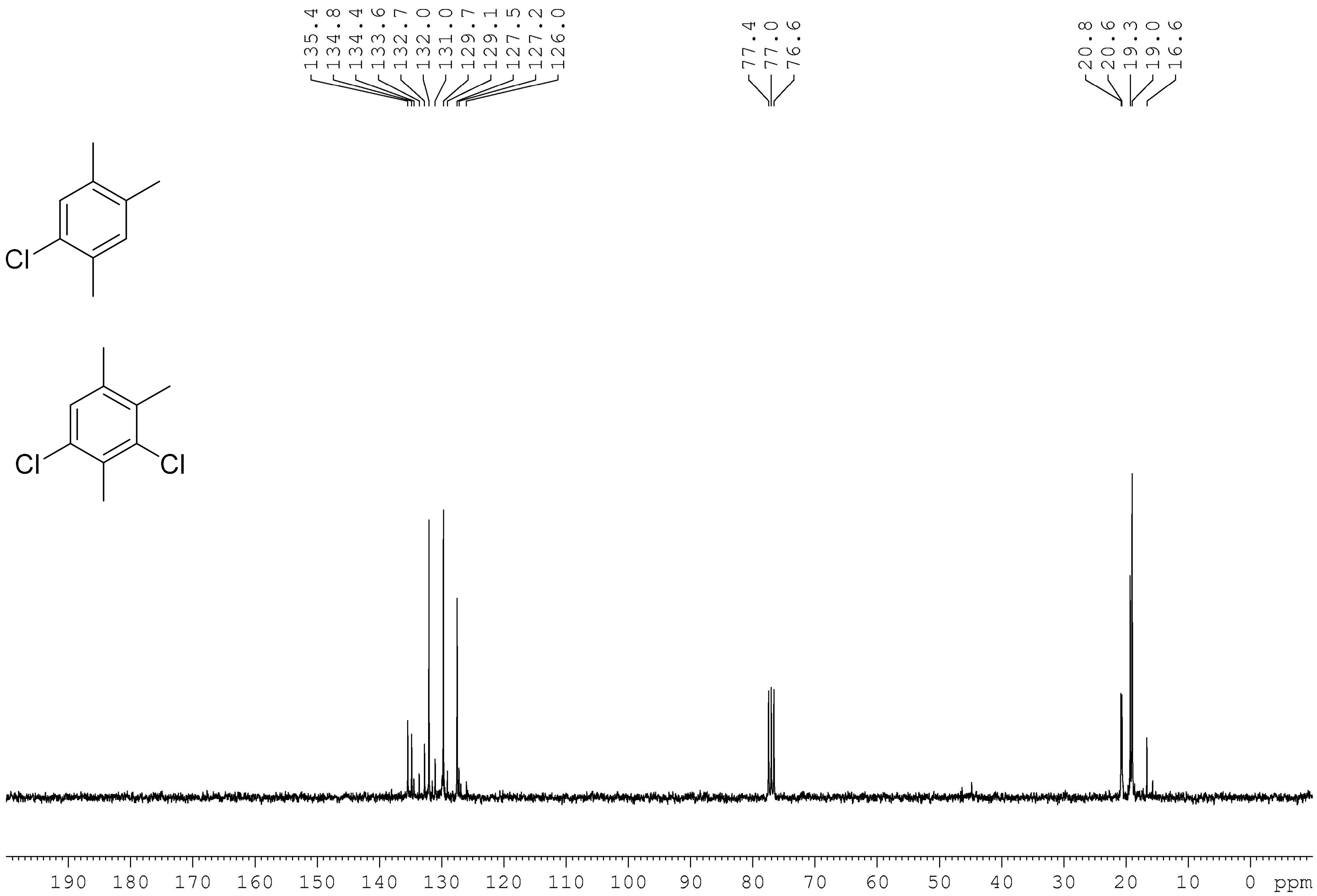

$^{13}C\{^{1}H\}$ NMR of compound **2m** and **2m'** (75 MHz, $CDCl_3$)

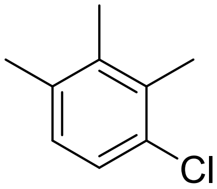

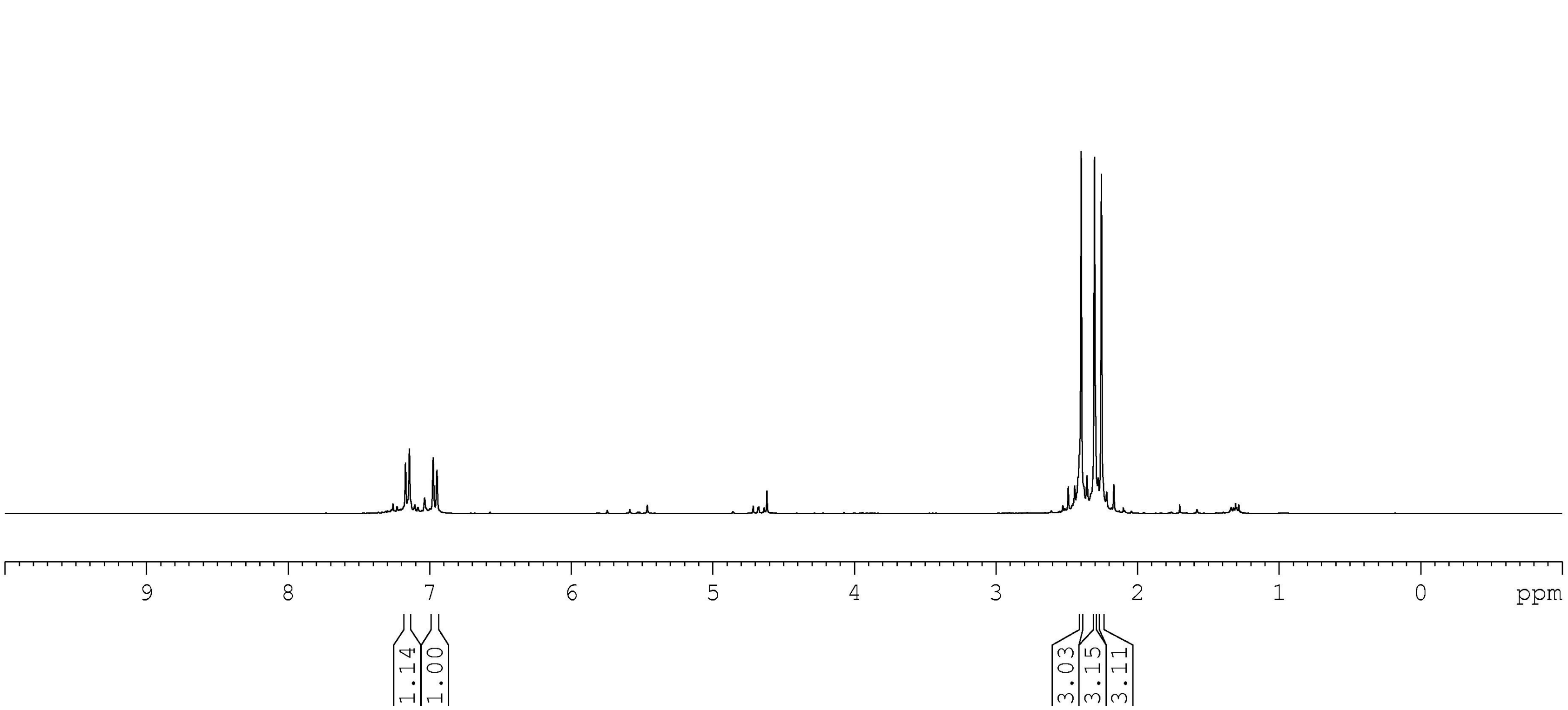

^{1}H NMR of compound **2n** (300 MHz, $CDCl_3$)

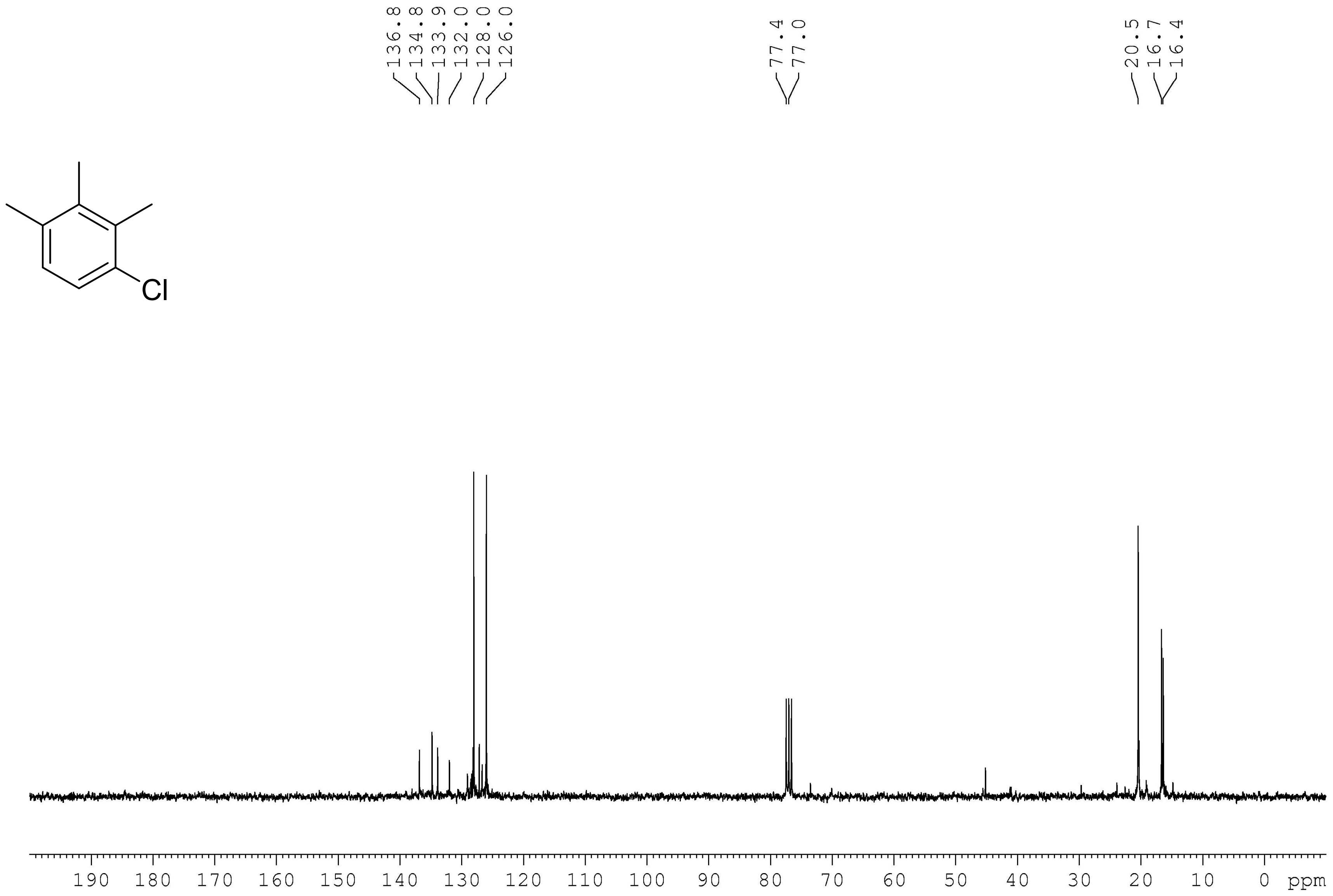

$^{13}C\{^{1}H\}$ NMR of compound **2n** (75 MHz, $CDCl_3$)

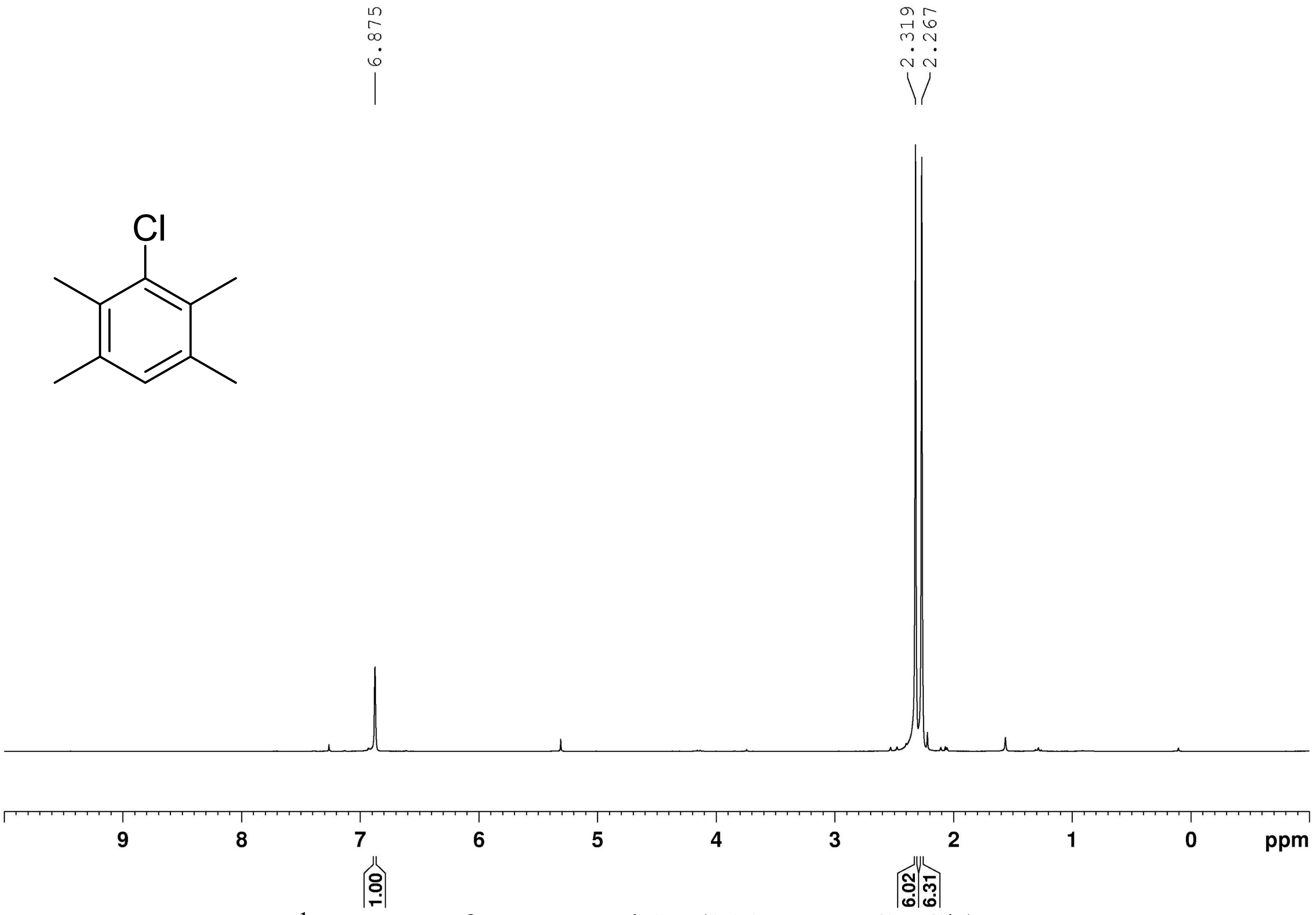

^{1}H NMR of compound **2o** (300 MHz, $CDCl_3$)

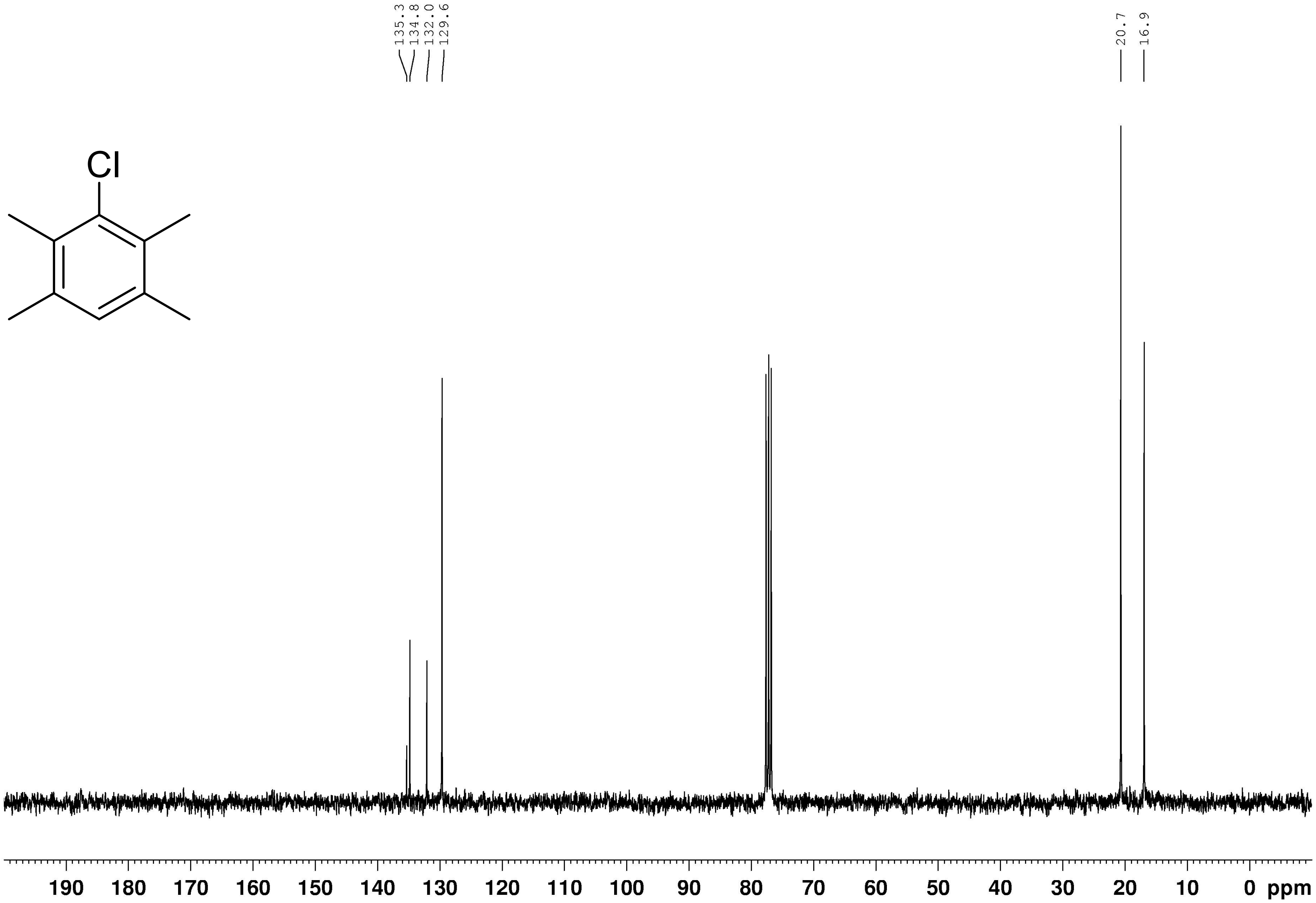

$^{13}C\{^{1}H\}$ NMR of compound **2o** (75 MHz, $CDCl_3$)

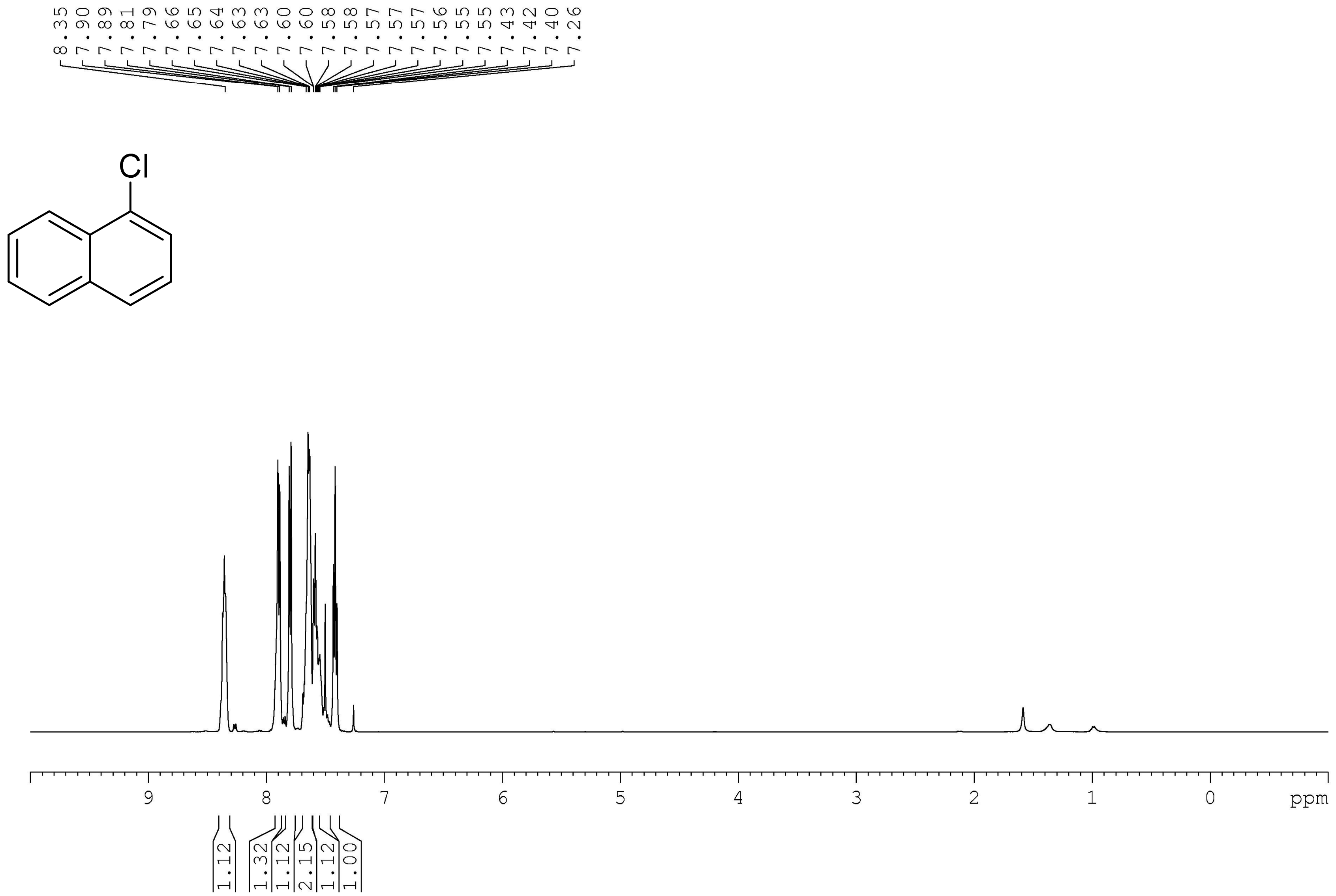

^{1}H NMR of compound **2p** (500 MHz, $CDCl_3$)

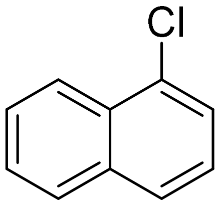

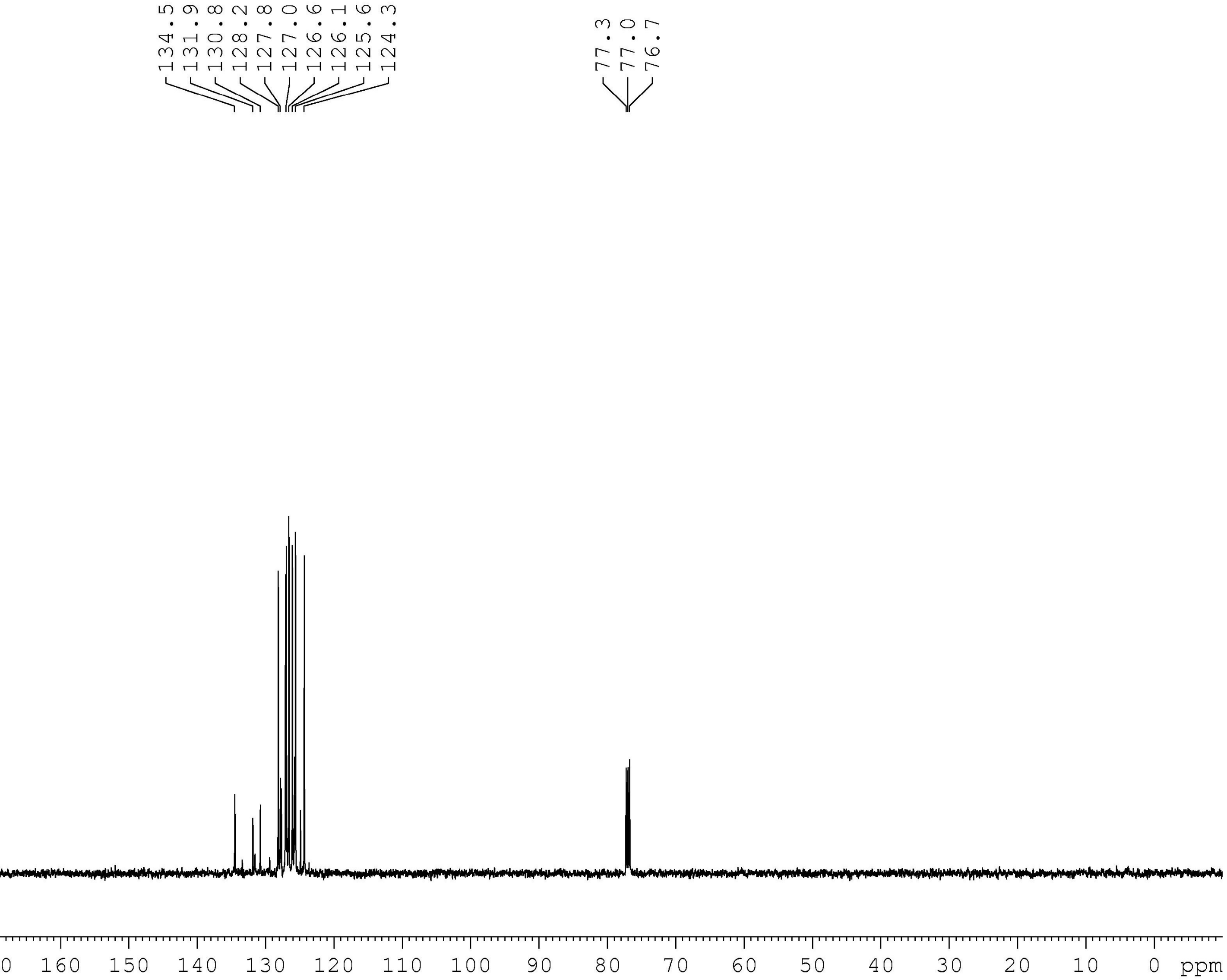

$^{13}C\{^1H\}$ NMR of compound **2p** (126 MHz, $CDCl_3$)

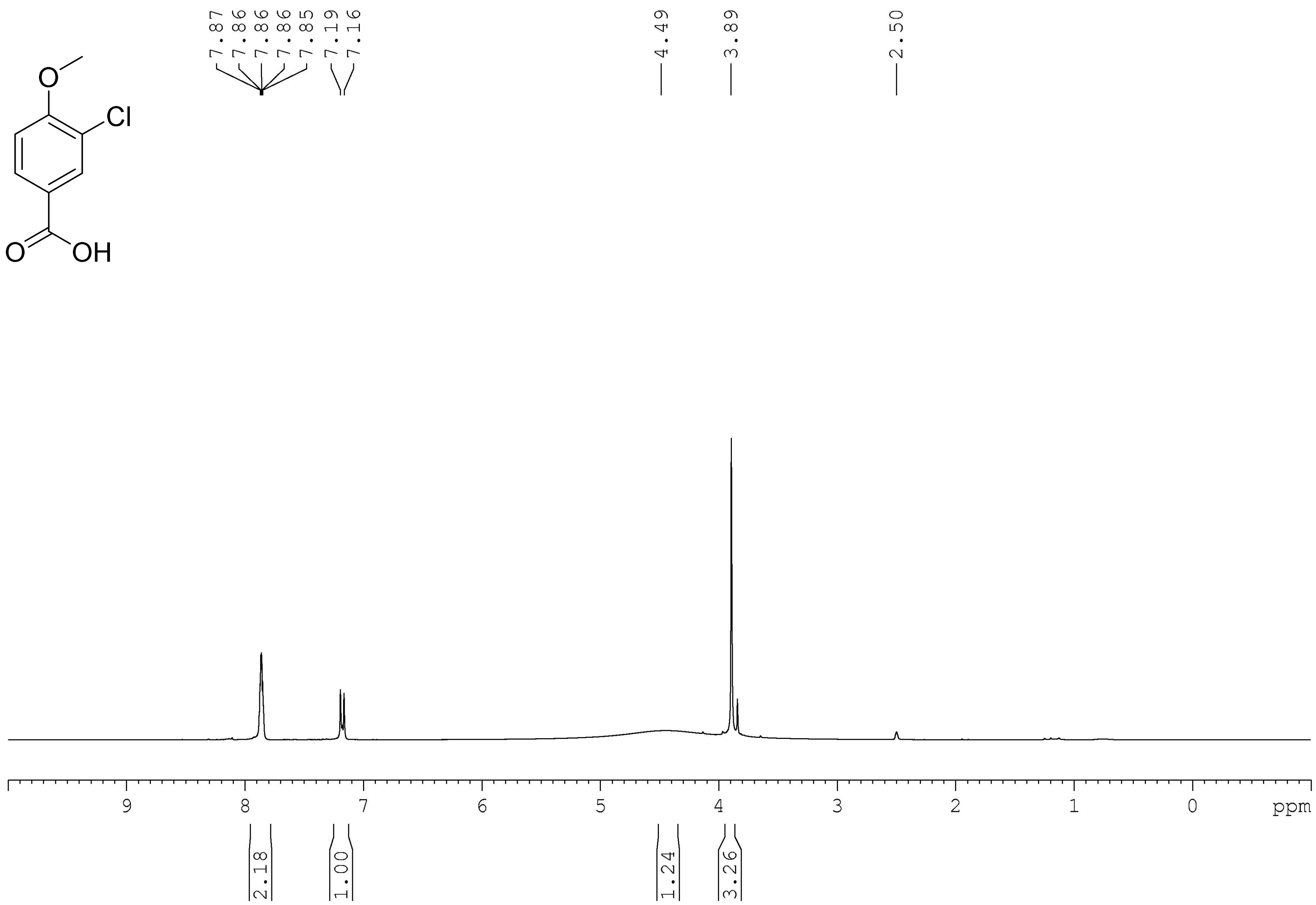

^{1}H NMR of compound **2q** (300 MHz, d_6-DMSO)

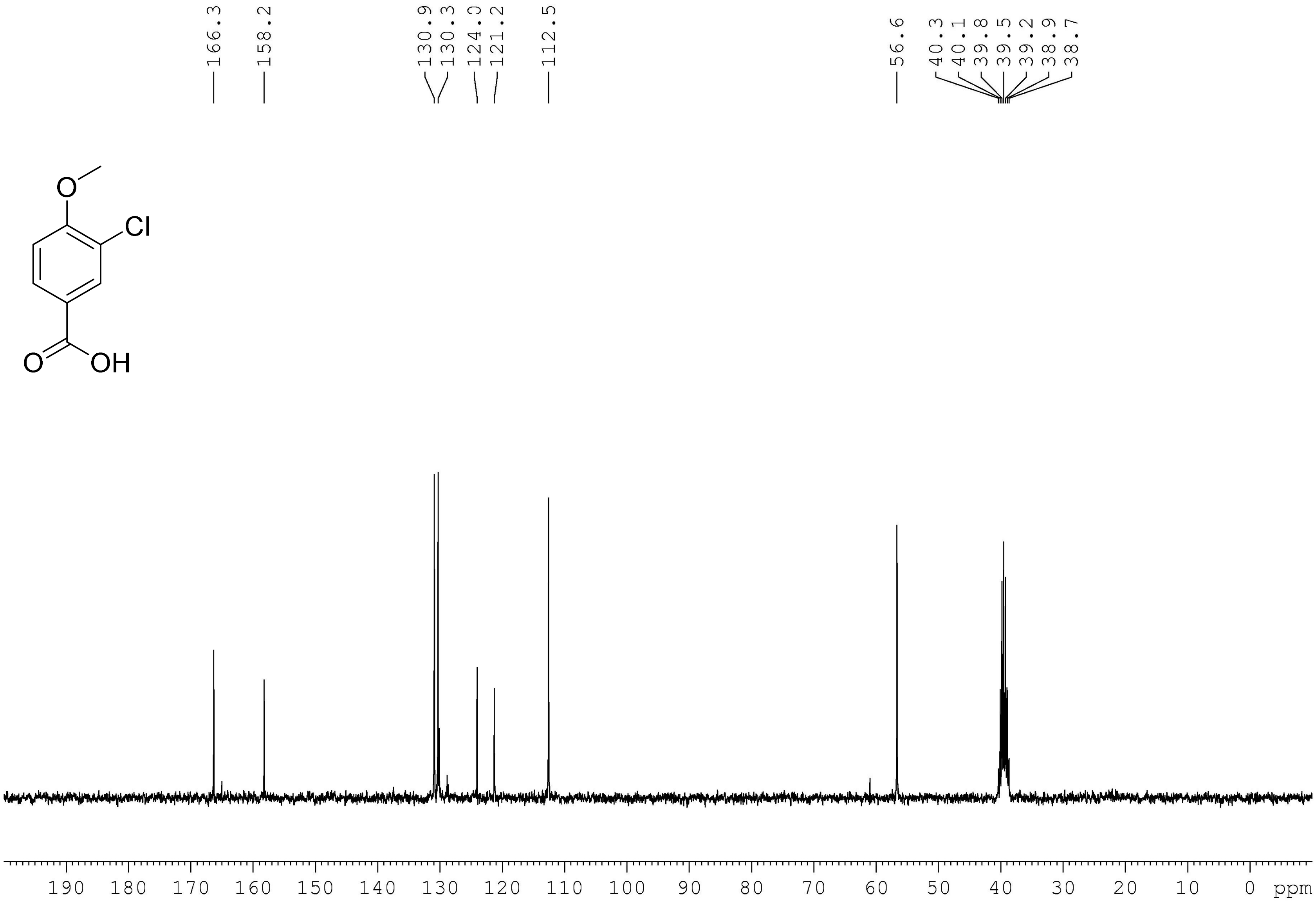

$^{13}C\{^{1}H\}$ NMR of compound **2q** (75 MHz, d_6-DMSO)

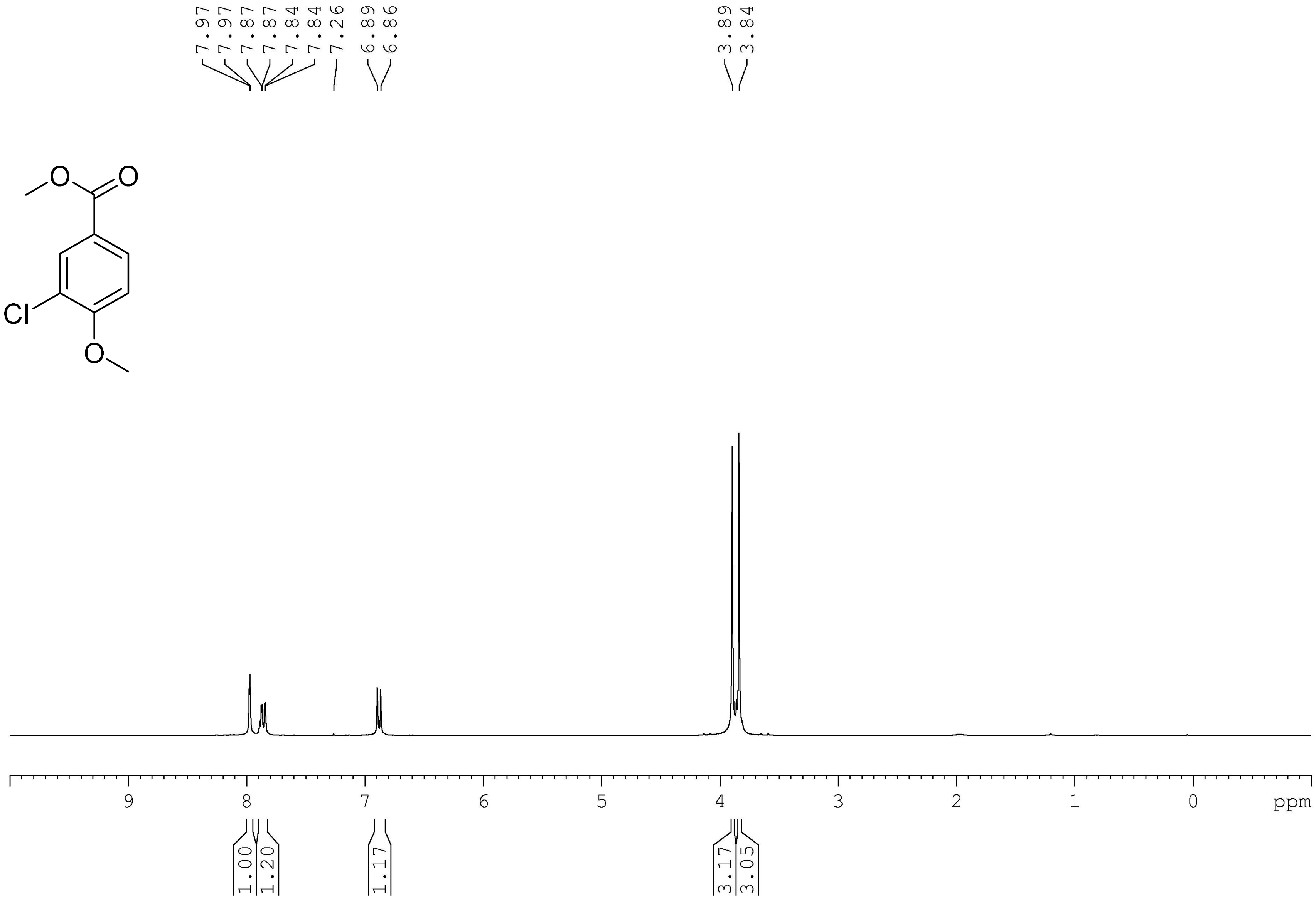

^{1}H NMR of compound **2r** (300 MHz, $CDCl_3$)

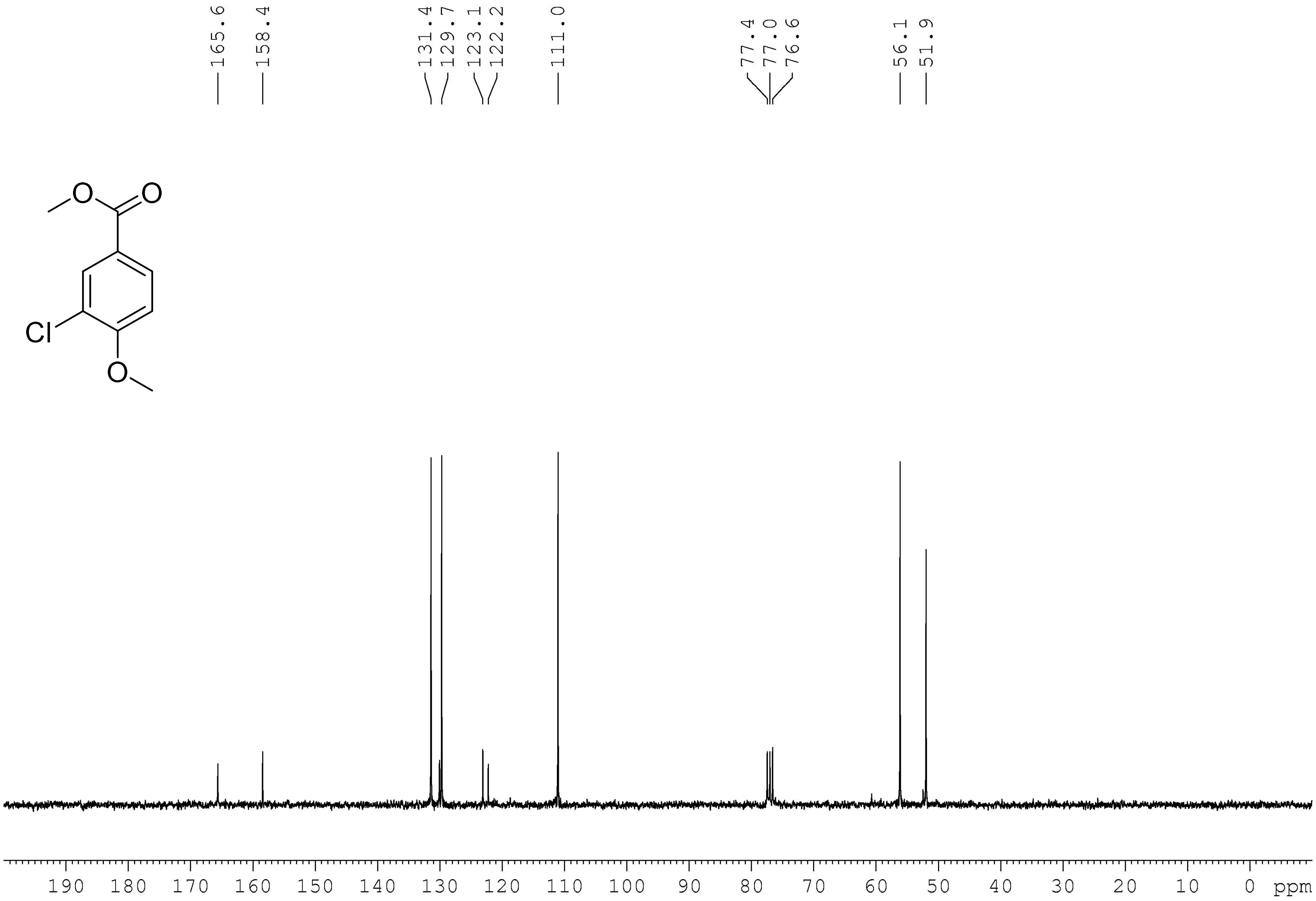

$^{13}C\{^{1}H\}$ NMR of compound **2r** (75 MHz, $CDCl_3$)

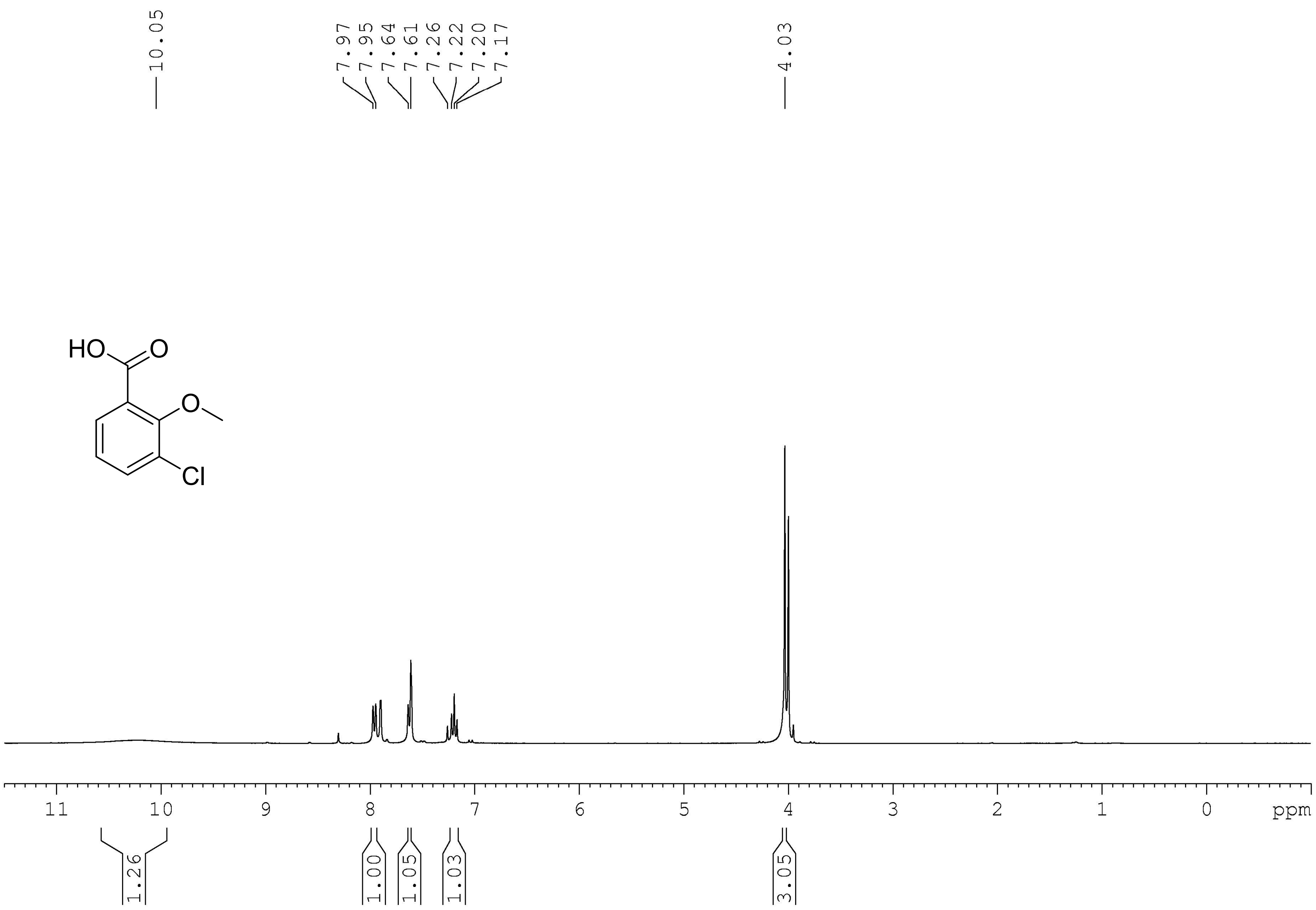

^{1}H NMR of compound **2s** (300 MHz, $CDCl_3$)

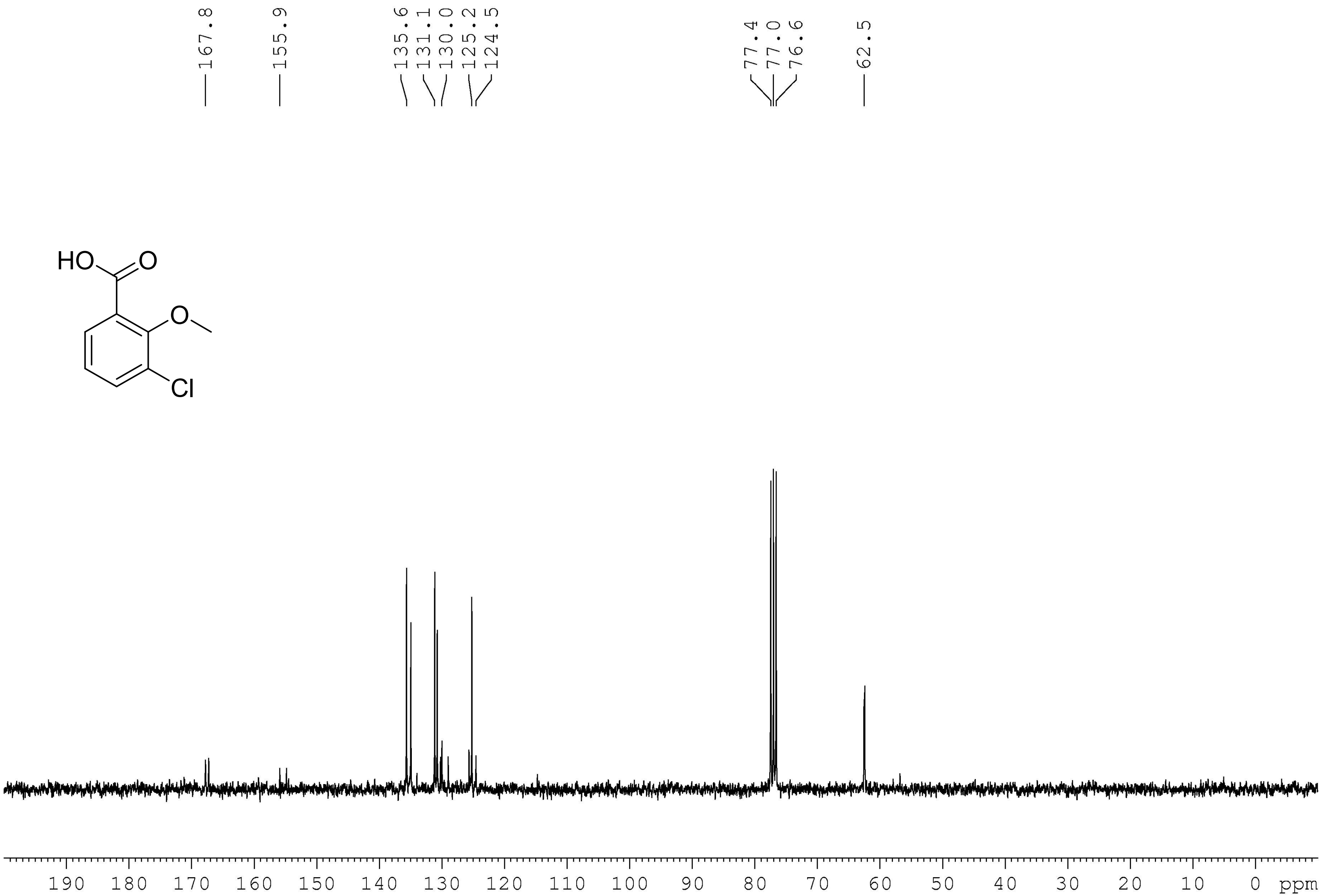

$^{13}C\{^{1}H\}$ NMR of compound **2s** (75 MHz, $CDCl_3$)

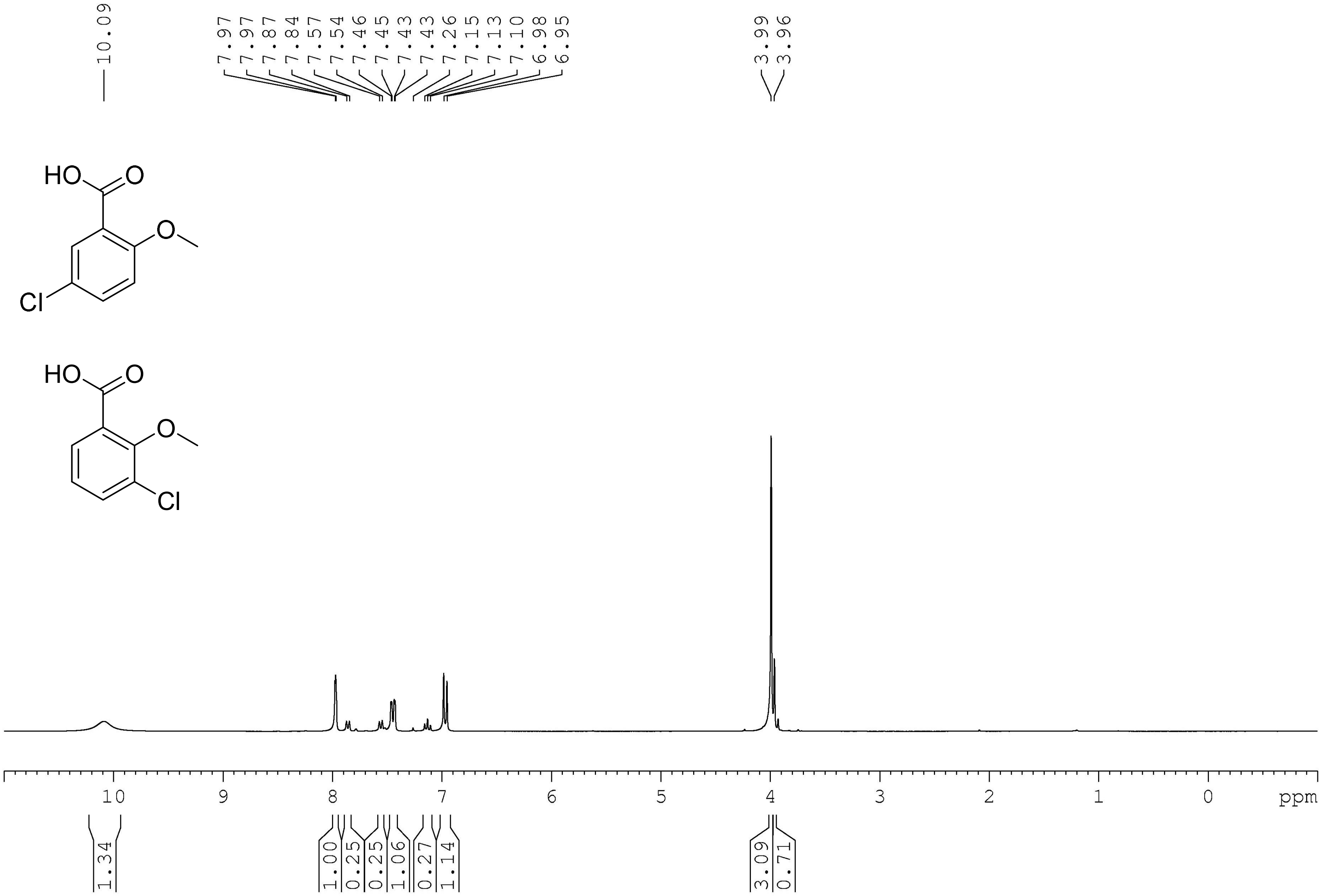

^{1}H NMR of compounds **2s** and **2s'** (300 MHz, $CDCl_3$)

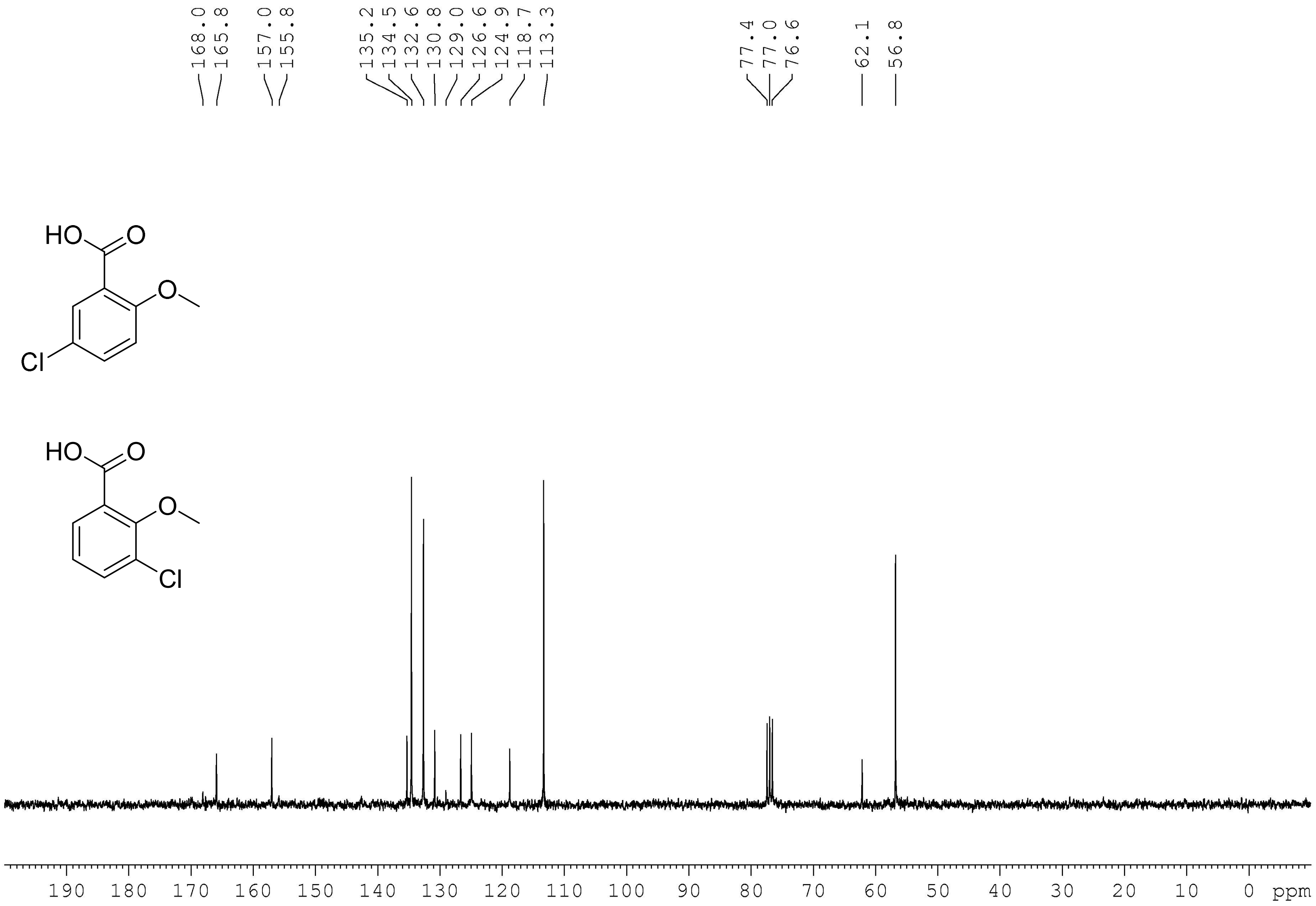

$^{13}C\{^{1}H\}$ NMR of compound **2s** and **2s'** (75 MHz, $CDCl_3$)

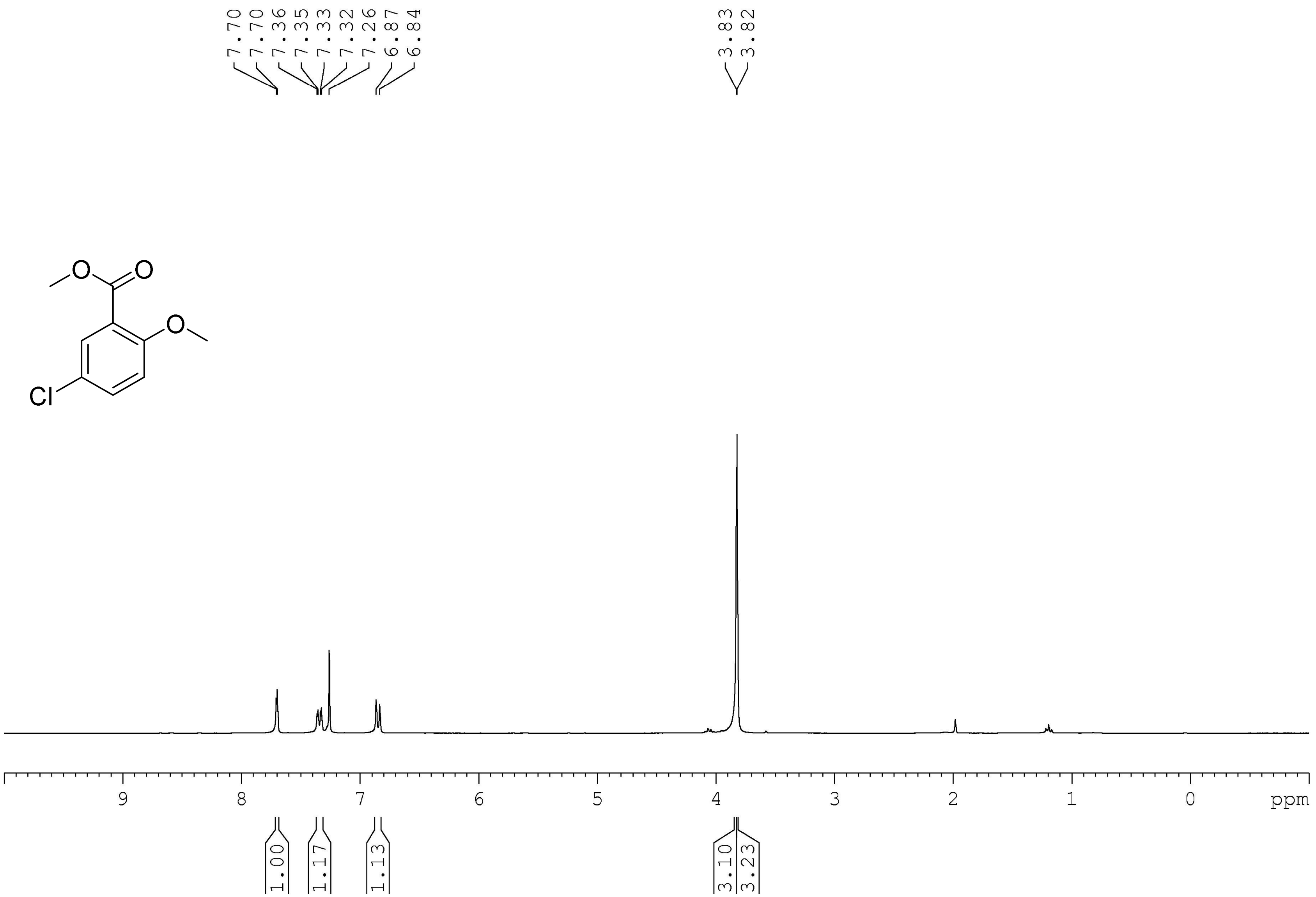

^{1}H NMR of compound **2t** (300 MHz, $CDCl_3$)

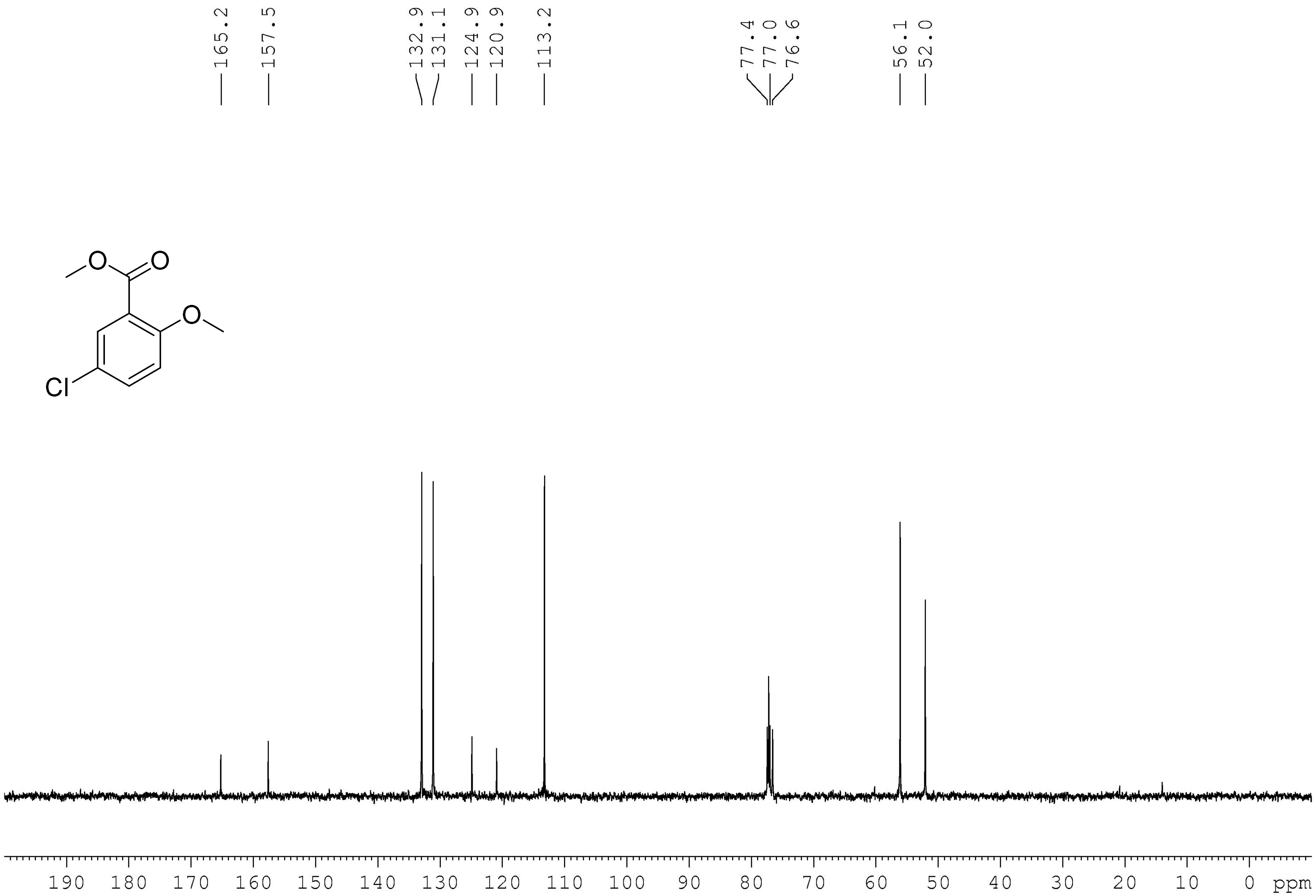

$^{13}C\{^{1}H\}$ NMR of compound **2t** (75 MHz, $CDCl_3$)

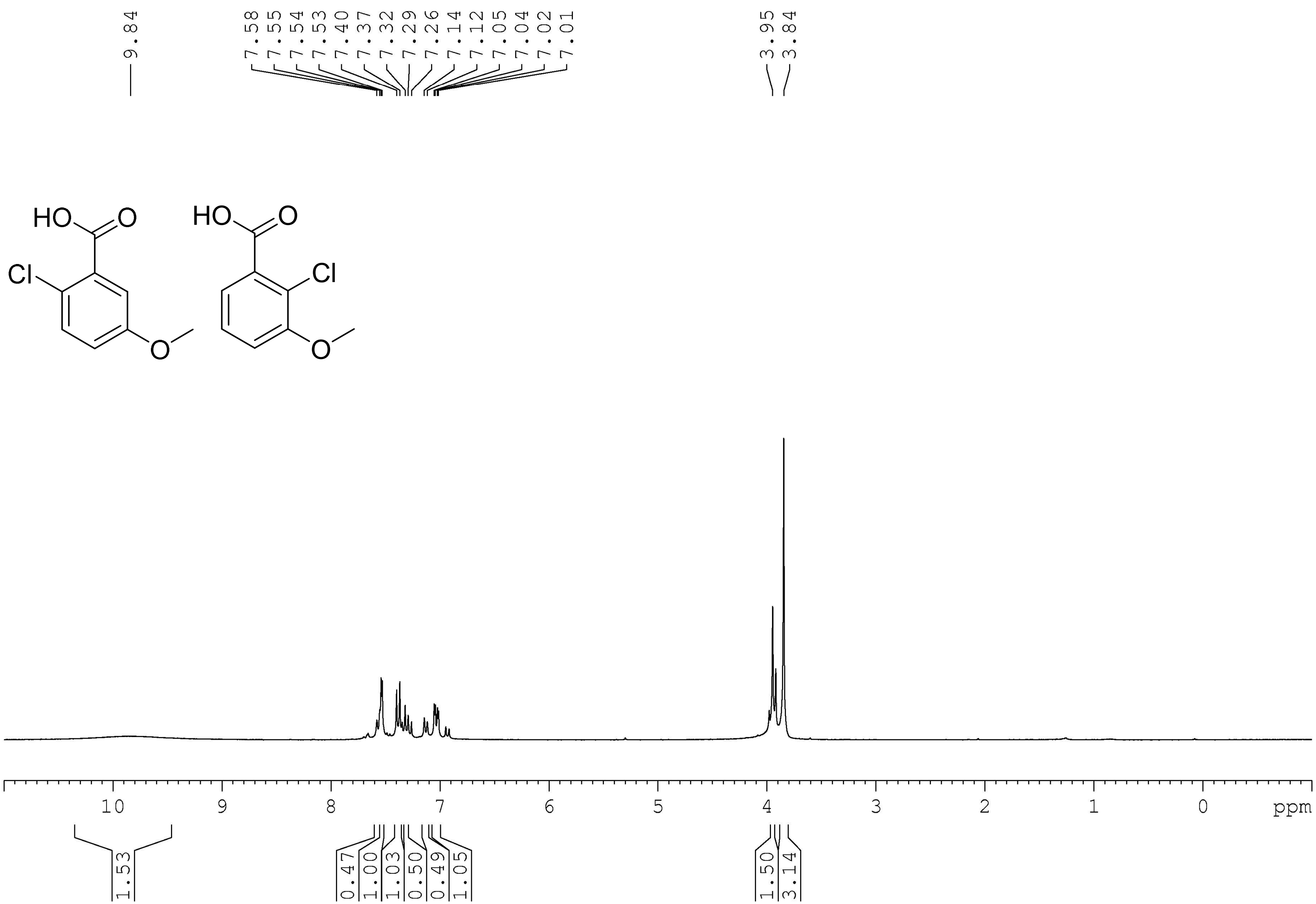

^{1}H NMR of compounds **2u** and **2u'** (300 MHz, $CDCl_3$)

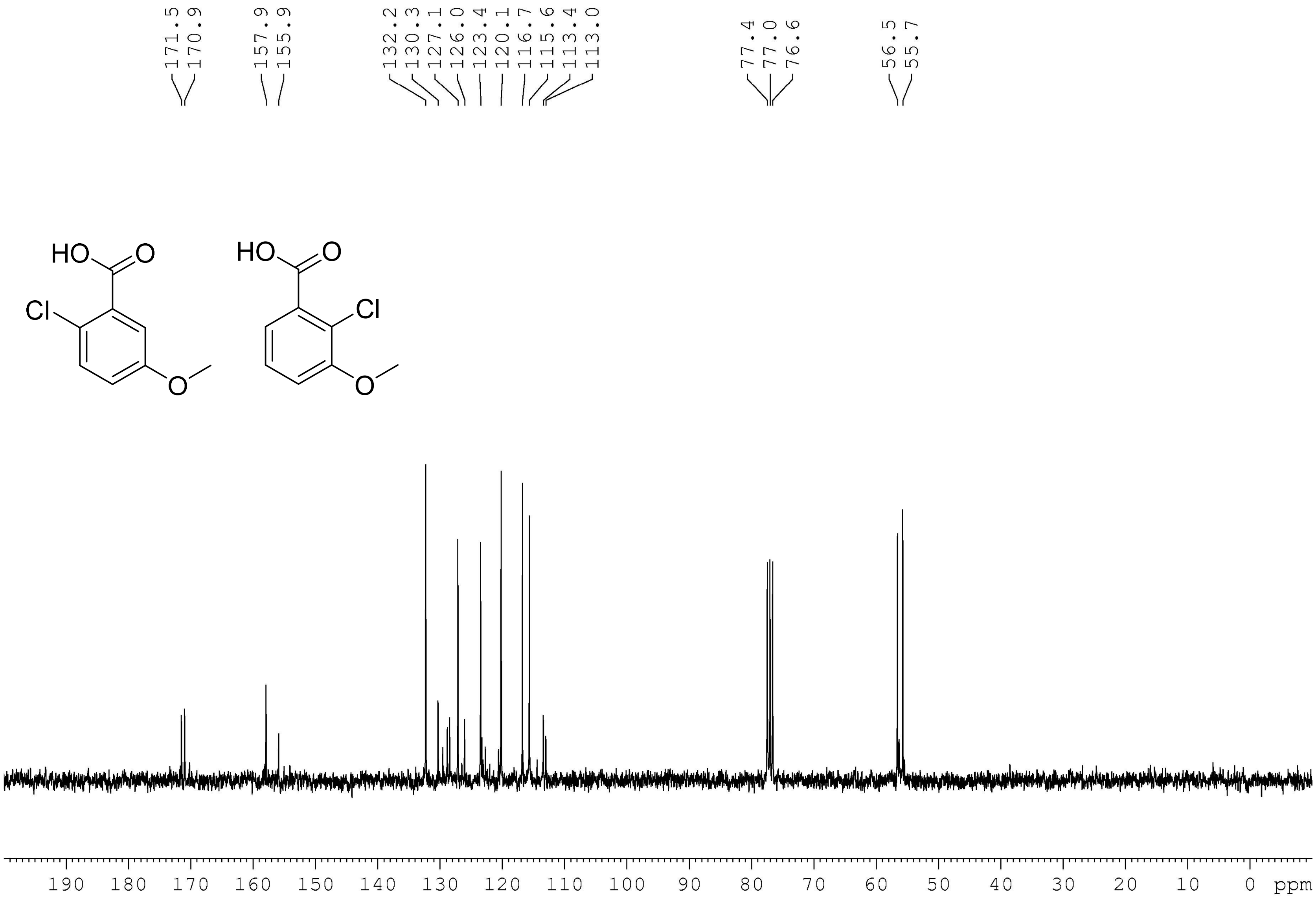

$^{13}C\{^1H\}$ NMR of compounds **2u** and **2u'** (75 MHz, $CDCl_3$)

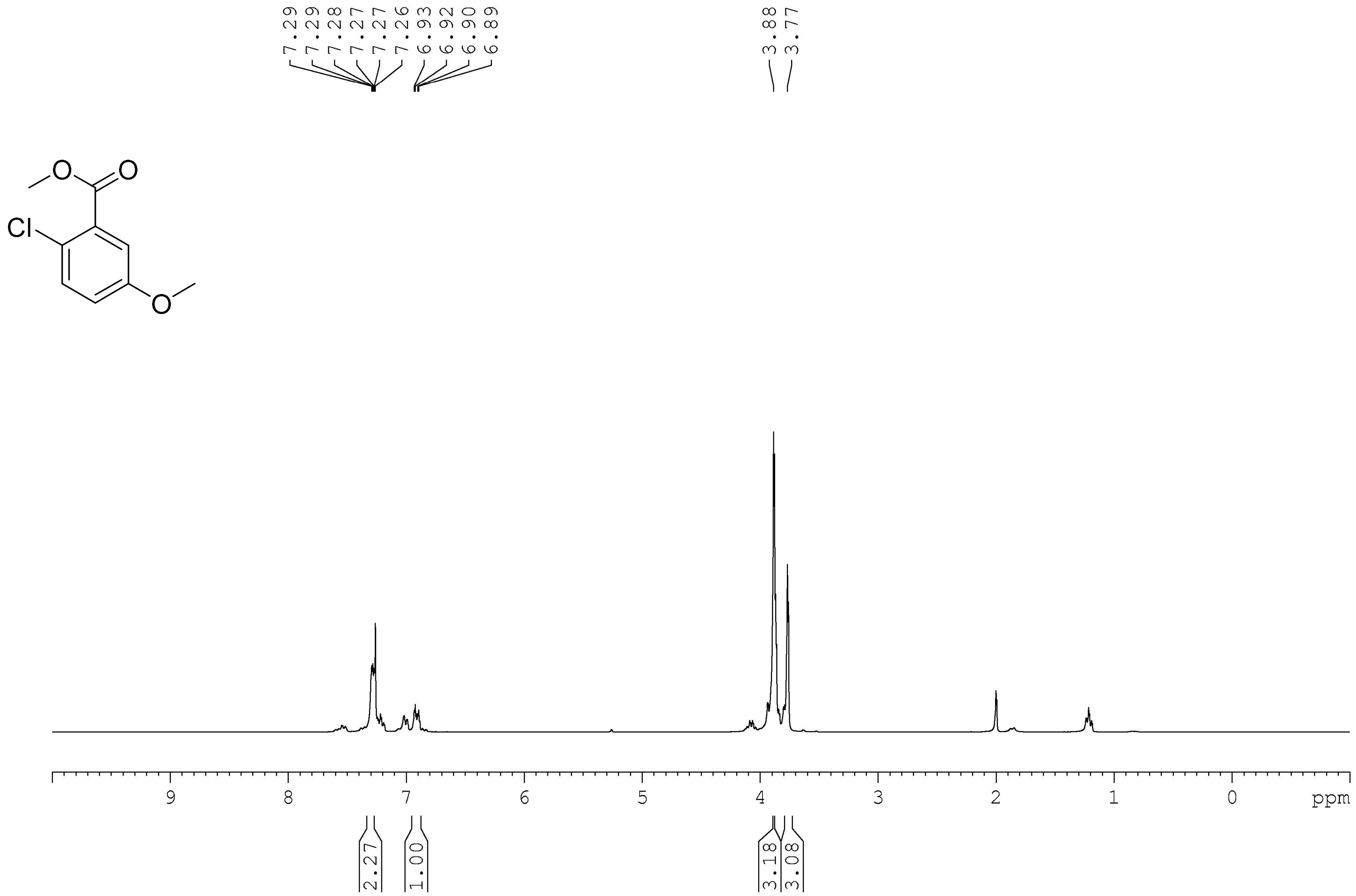

^{1}H NMR of compound **2v** (300 MHz, $CDCl_3$)

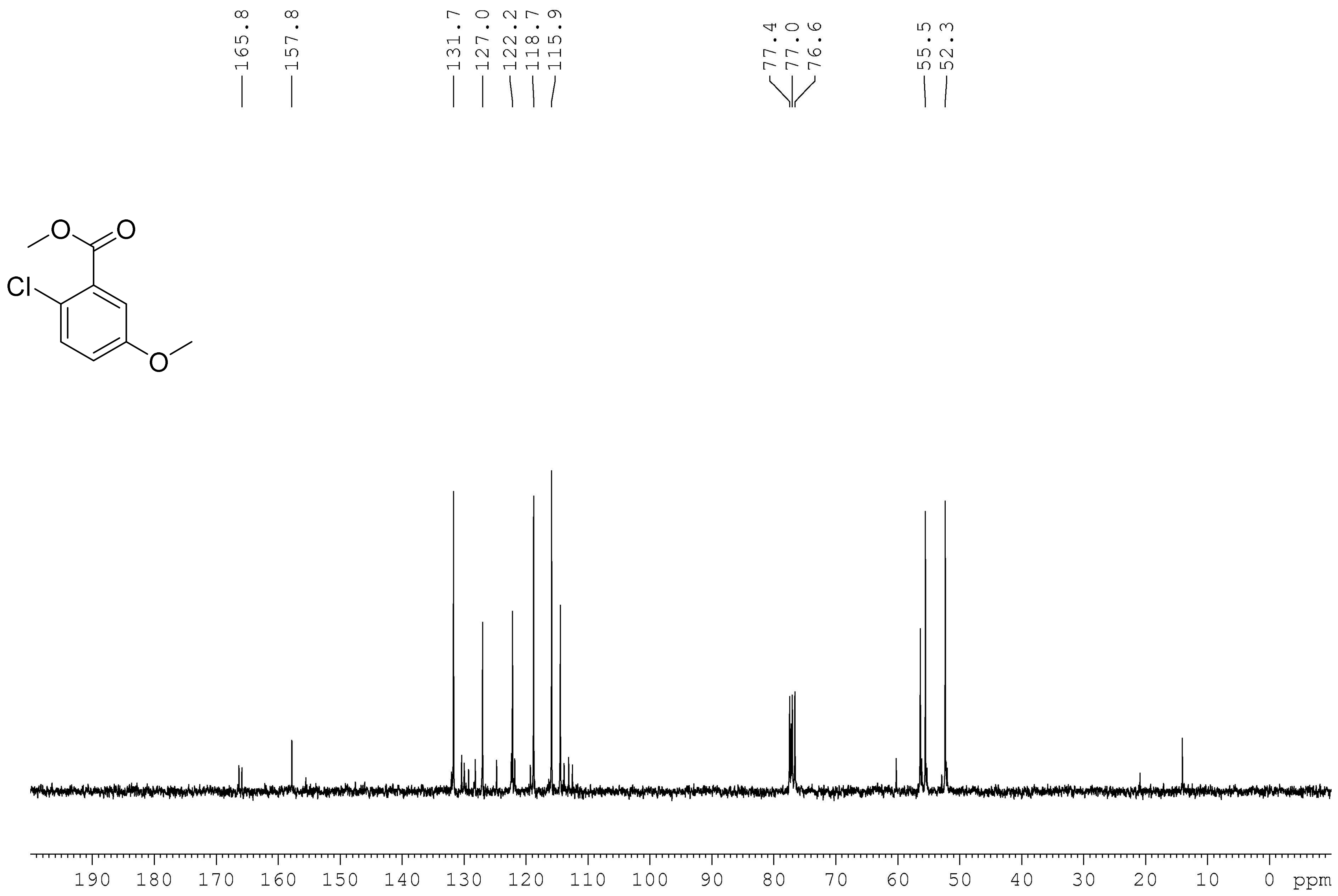

$^{13}C\{^{1}H\}$ NMR of compound **2v** (75 MHz, $CDCl_3$)

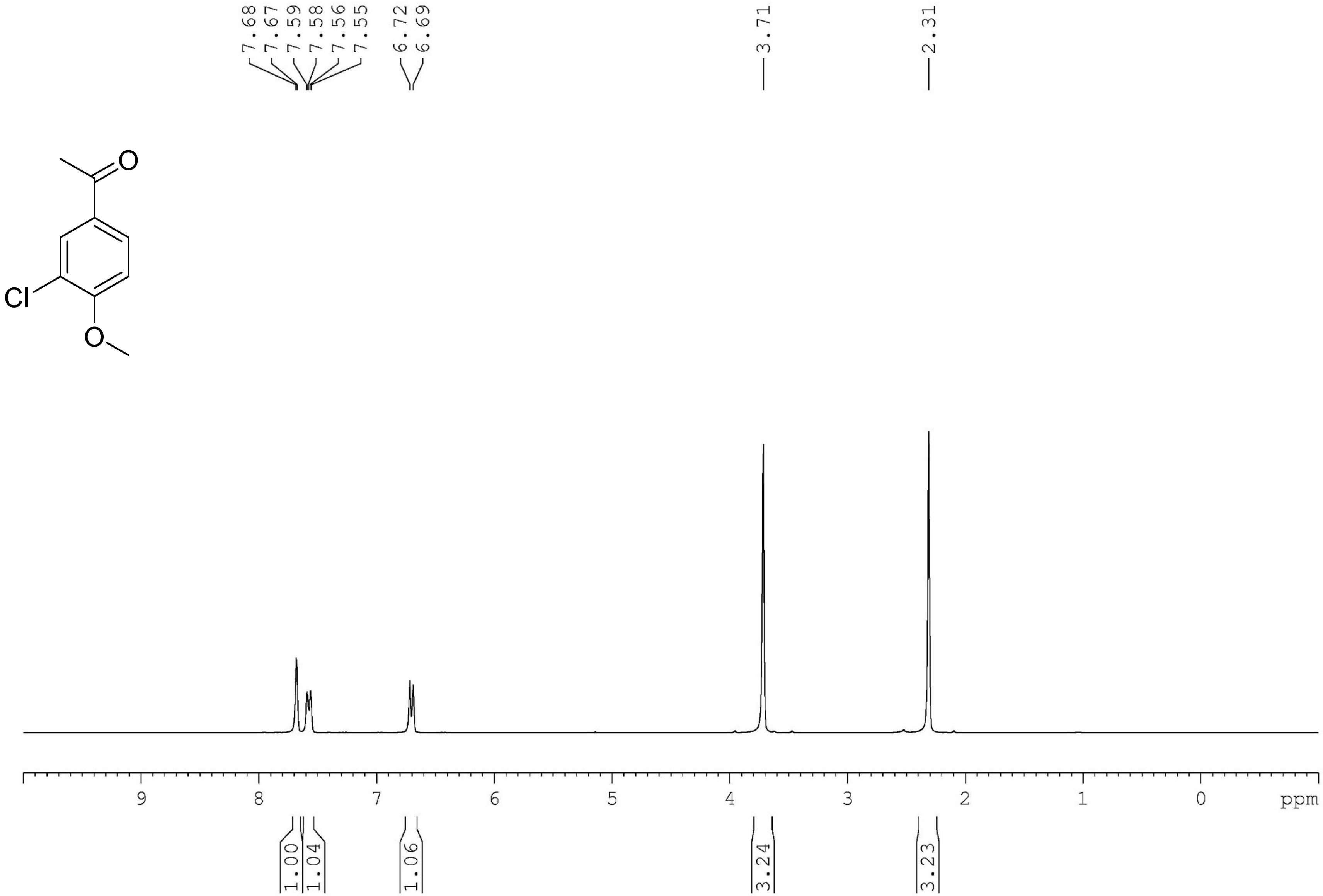

^{1}H NMR of compound **2w** (300 MHz, $CDCl_3$)

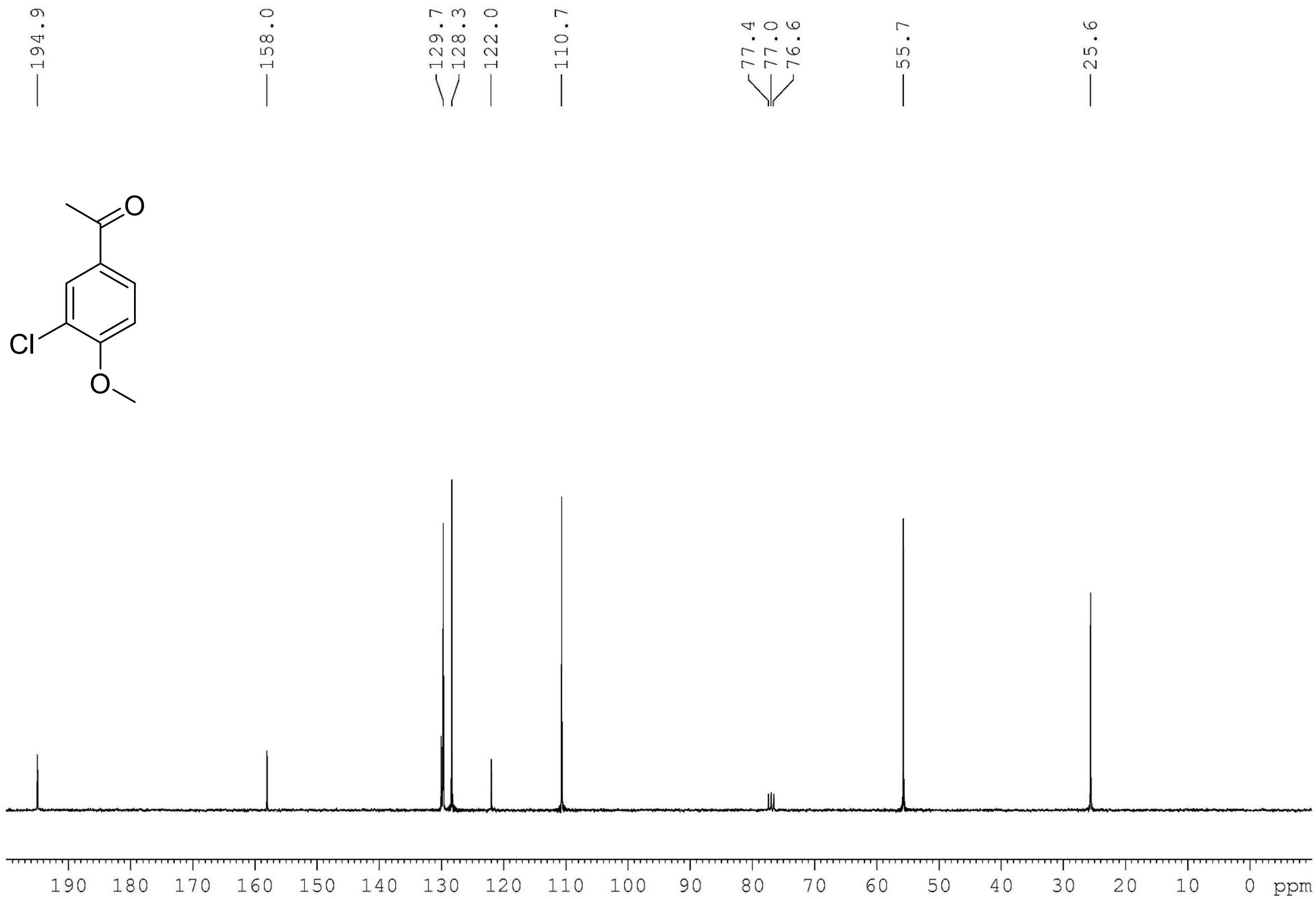

$^{13}C\{^{1}H\}$ NMR of compound **2w** (75 MHz, $CDCl_3$)

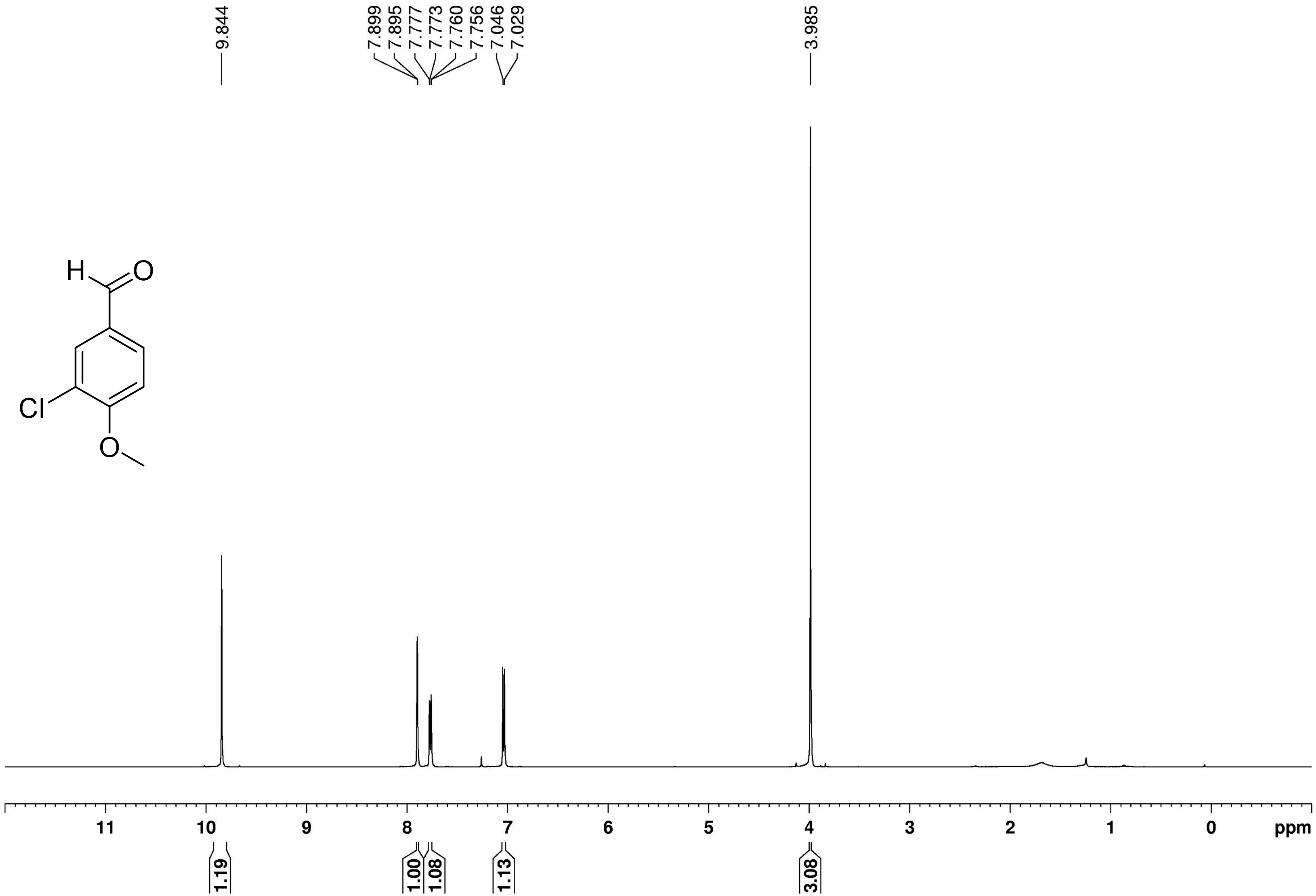

^{1}H NMR of compound **2x** (500 MHz, $CDCl_3$)

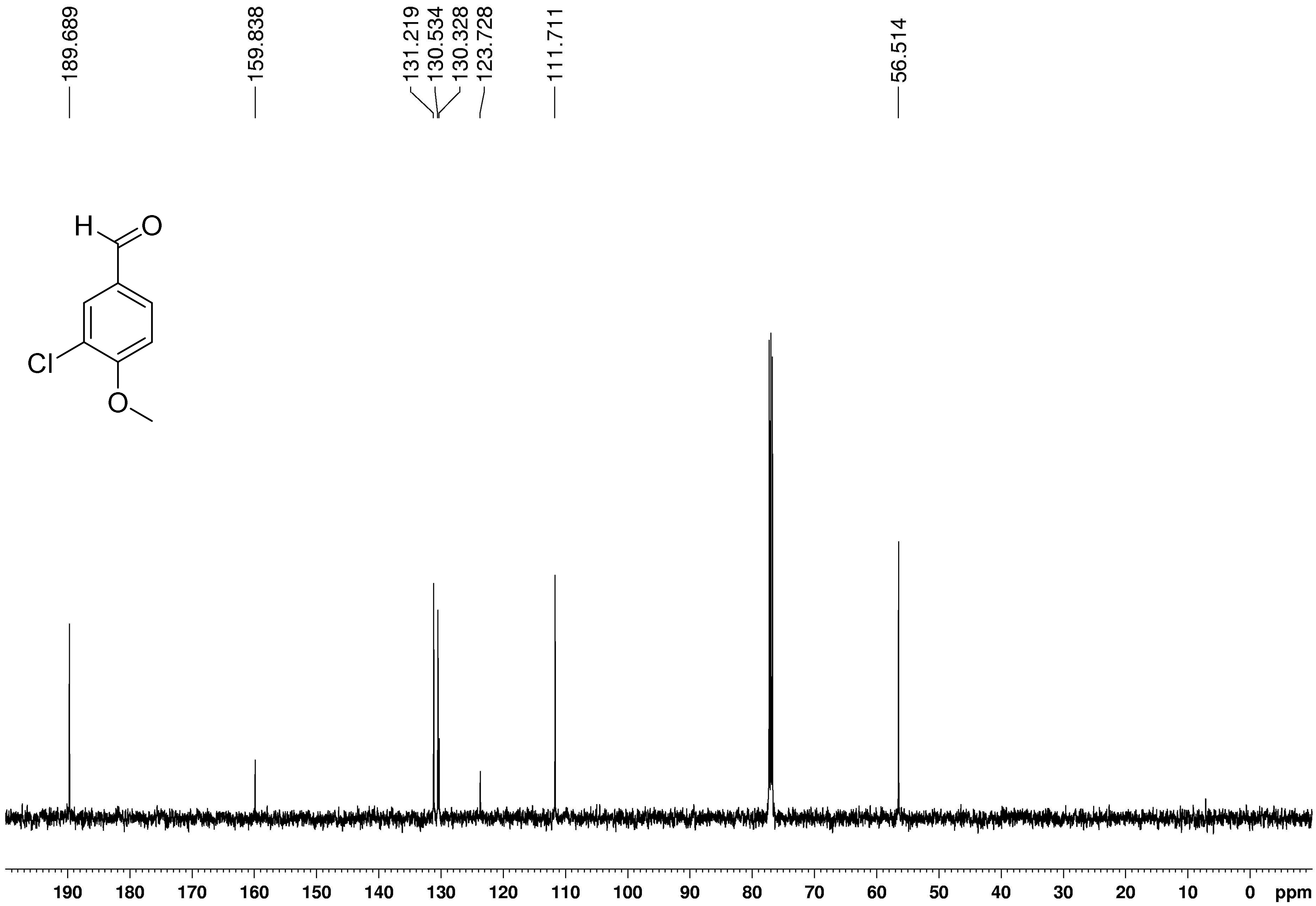

$^{13}C\{^{1}H\}$ NMR of compound **2x** (126 MHz, $CDCl_3$)

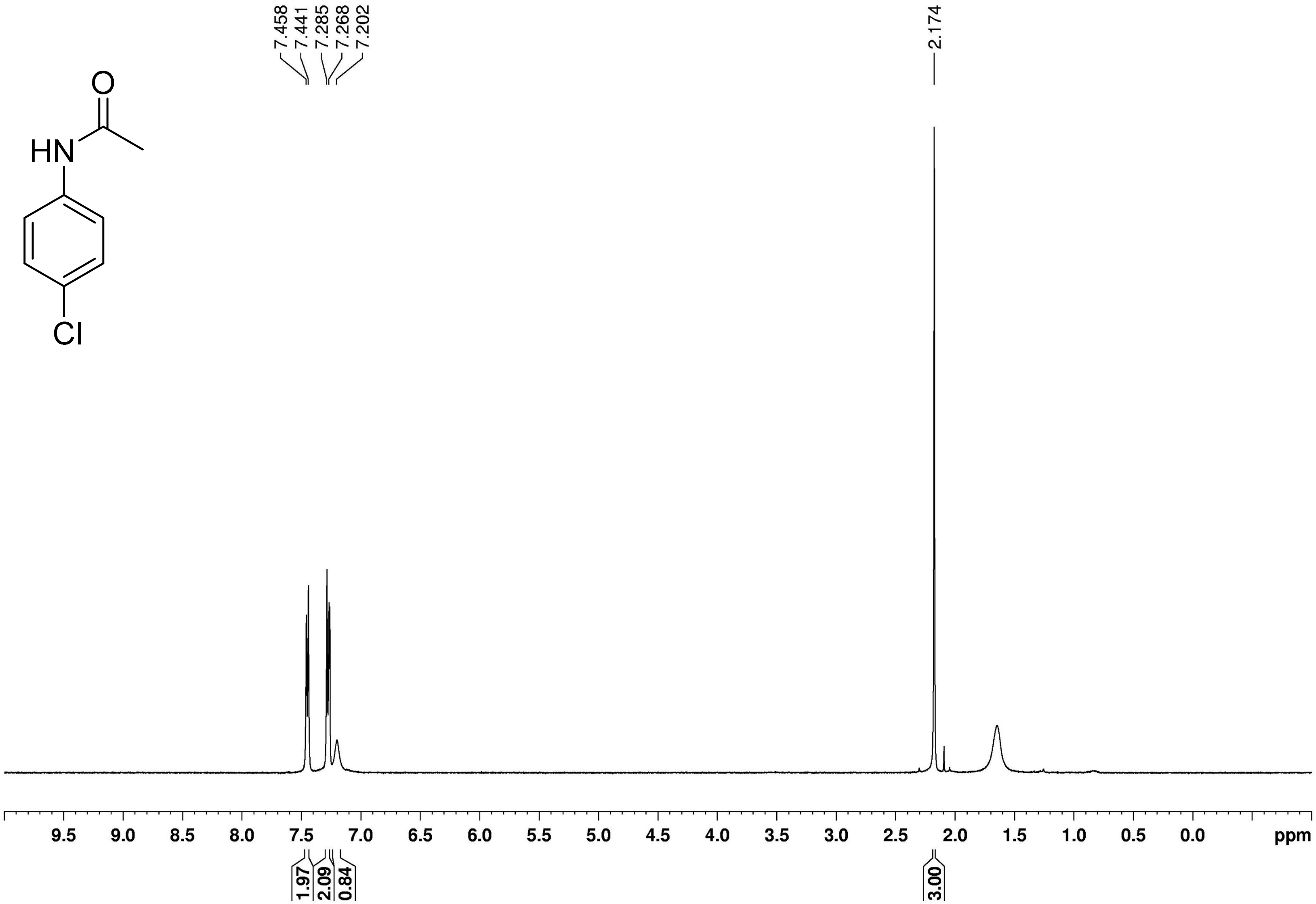

^{1}H NMR of compound **2y** (500 MHz, $CDCl_3$)

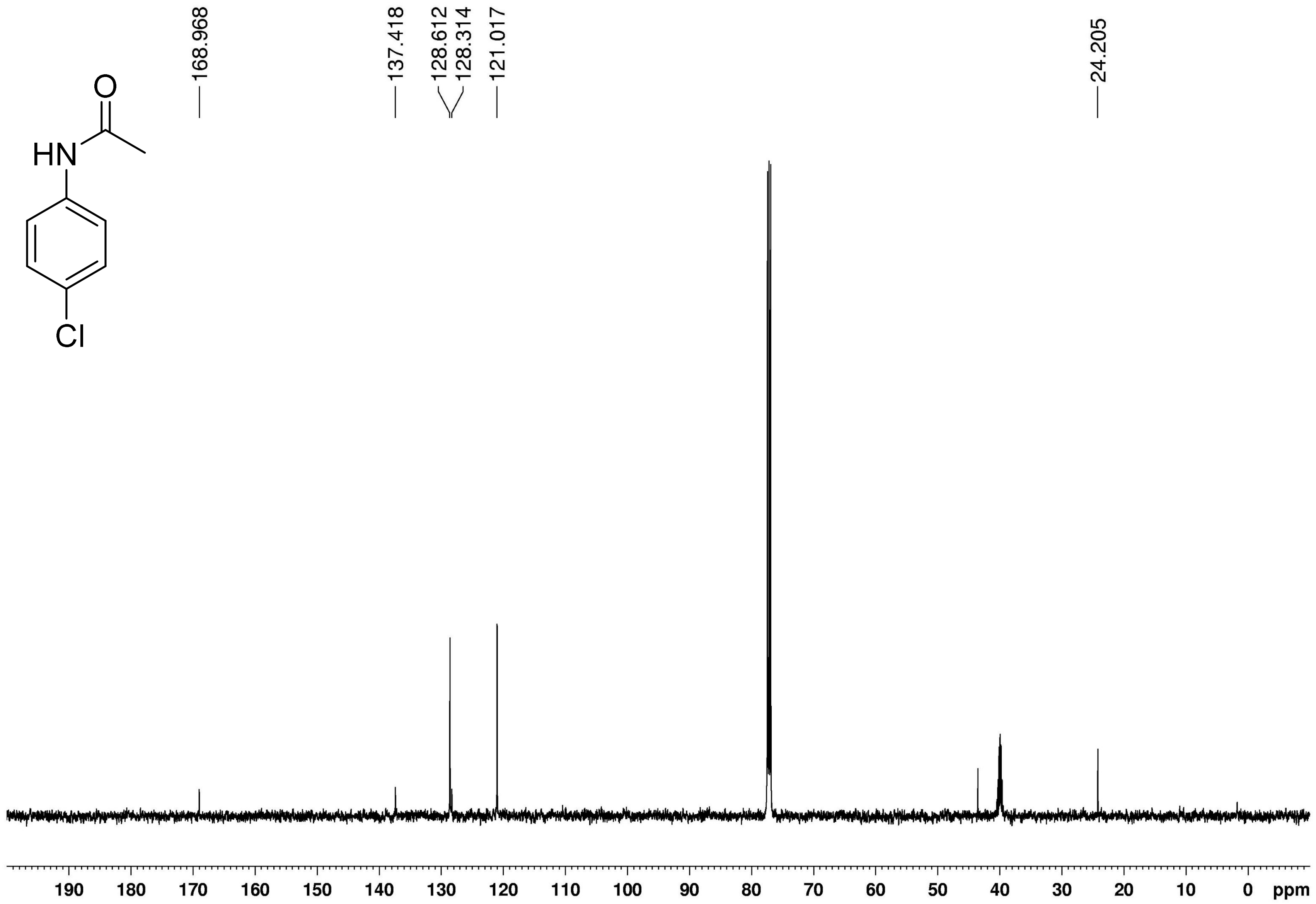

$^{13}C\{^1H\}$ NMR of compound **2y** (126 MHz, $CDCl_3$ + 2 drops of DMSO)

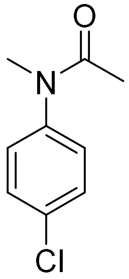

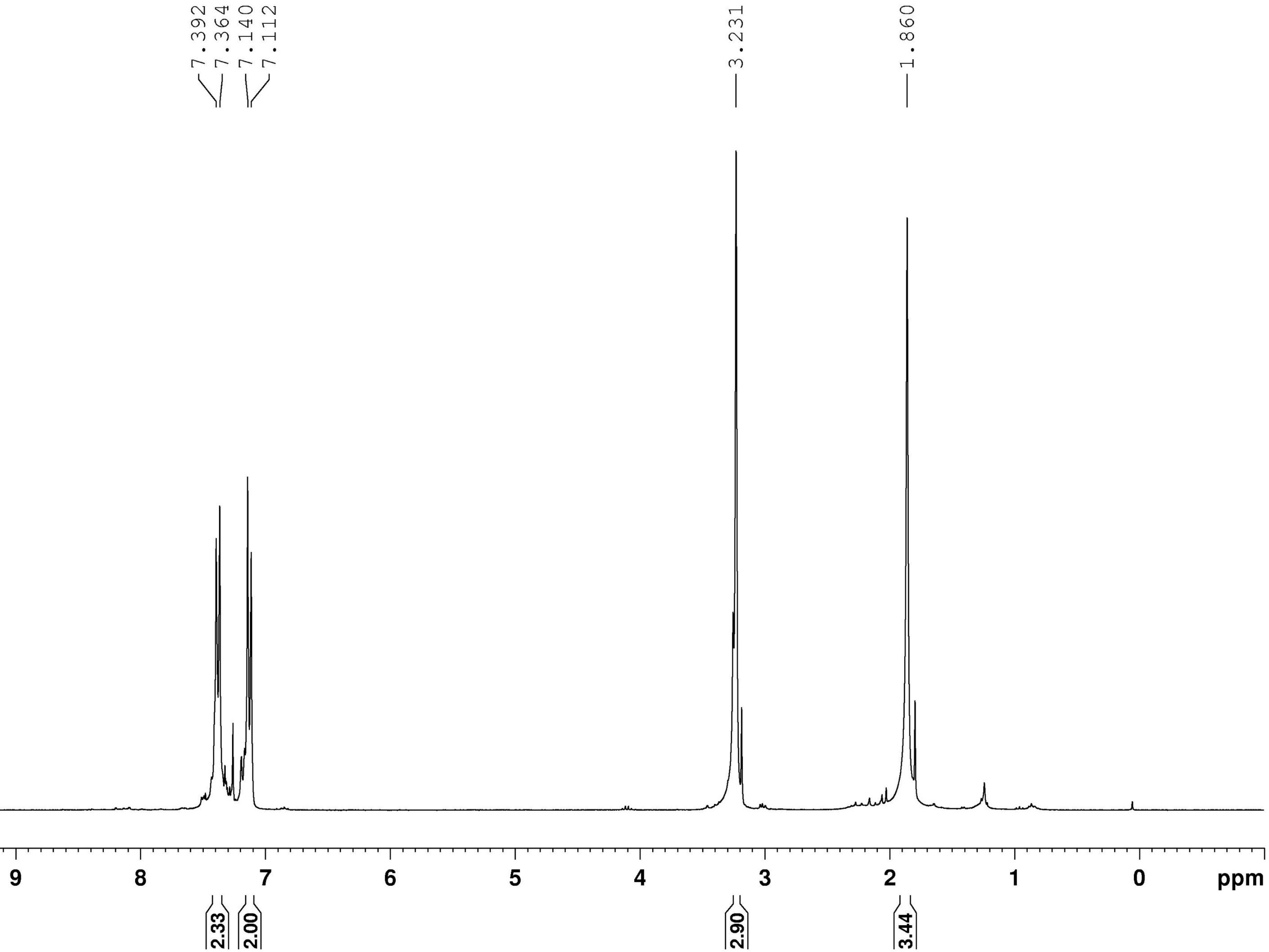

^{1}H NMR of compound **2z** (300 MHz, $CDCl_3$)

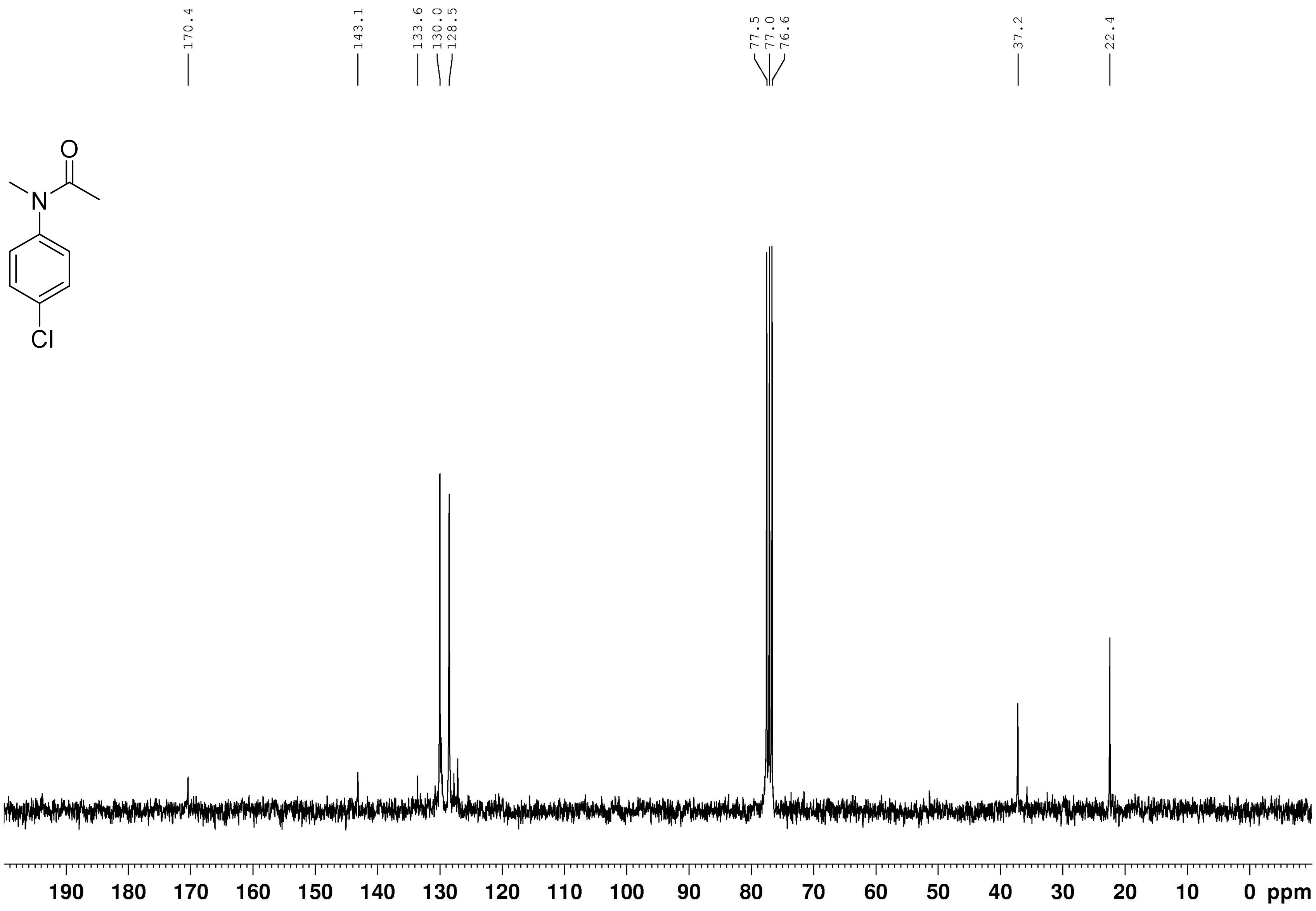

$^{13}C\{^1H\}$ NMR of compound **2z** (75 MHz, $CDCl_3$)

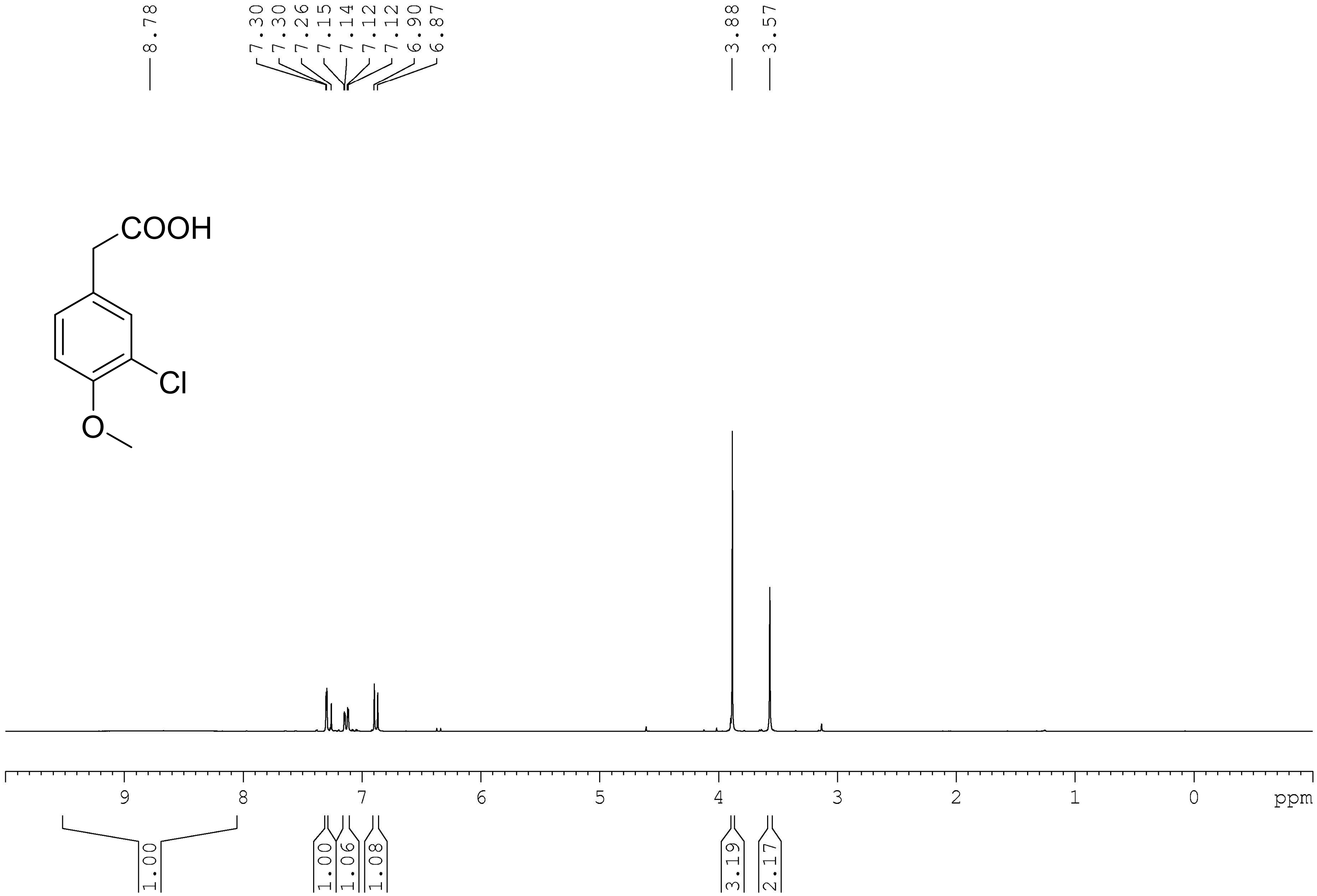

^{1}H NMR of compound **2aA** (300 MHz, $CDCl_3$)

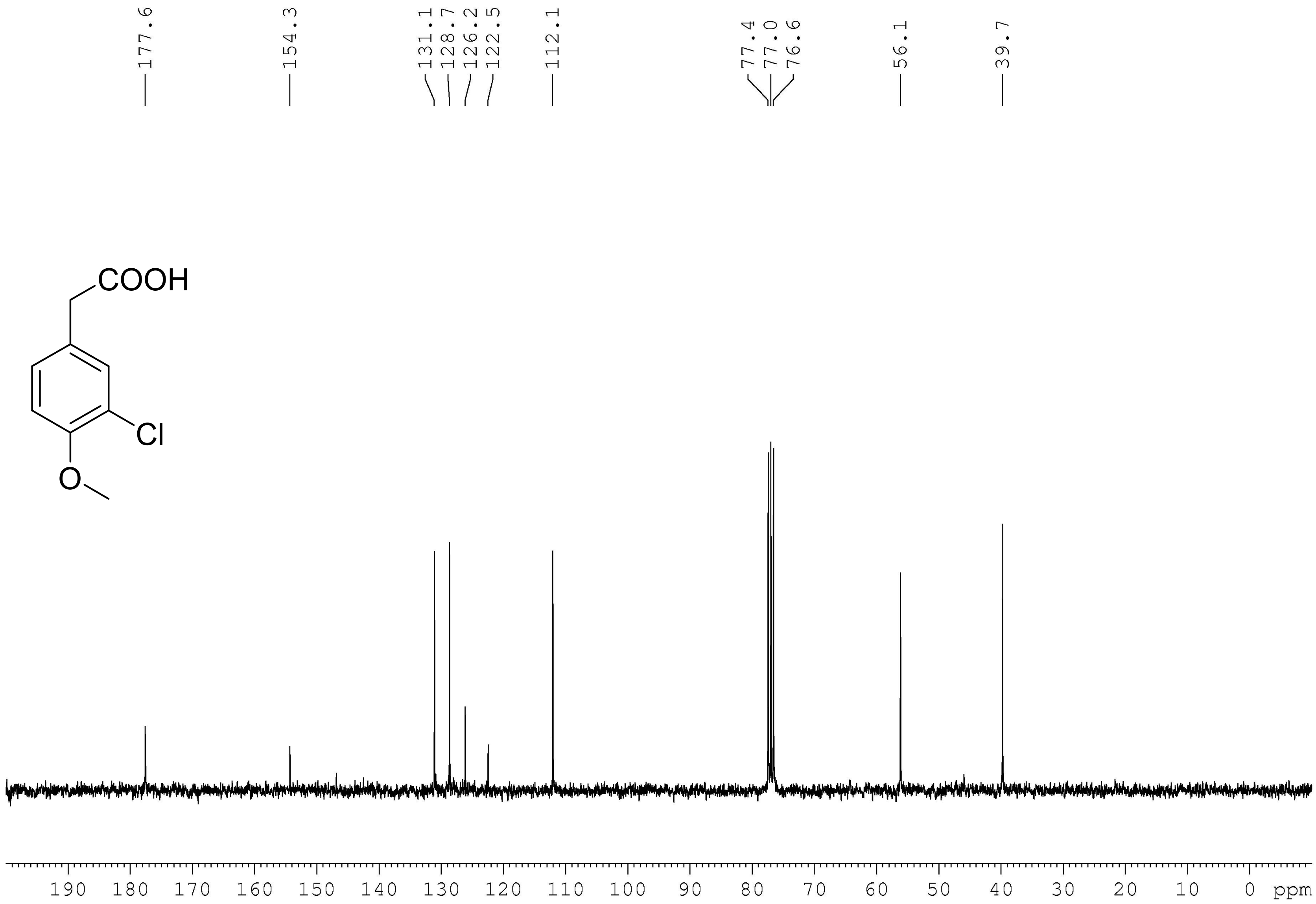

$^{13}C\{^{1}H\}$ NMR of compound **2aA** (75 MHz, $CDCl_3$)

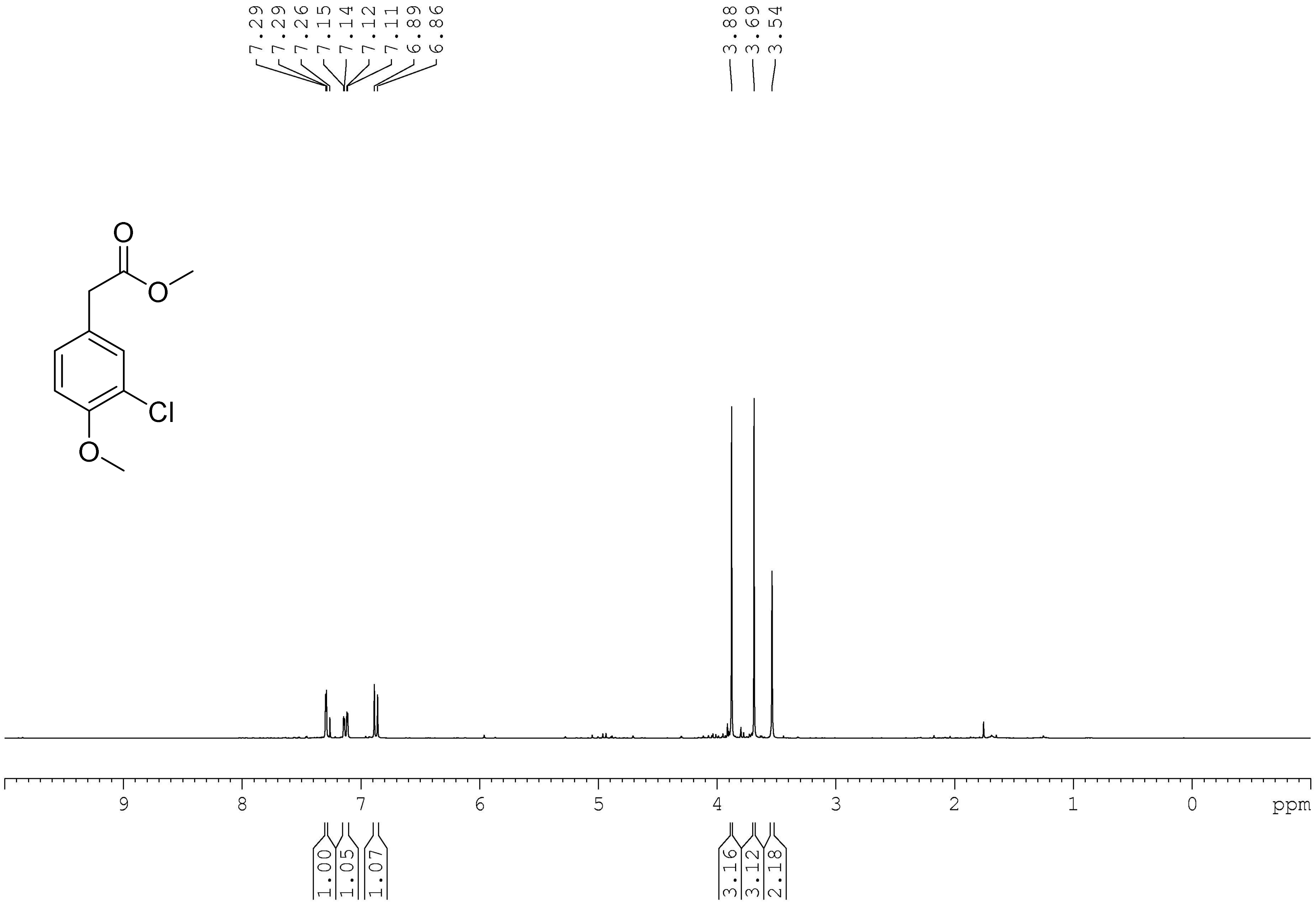

^{1}H NMR of compound **2aE** (300 MHz, $CDCl_3$)

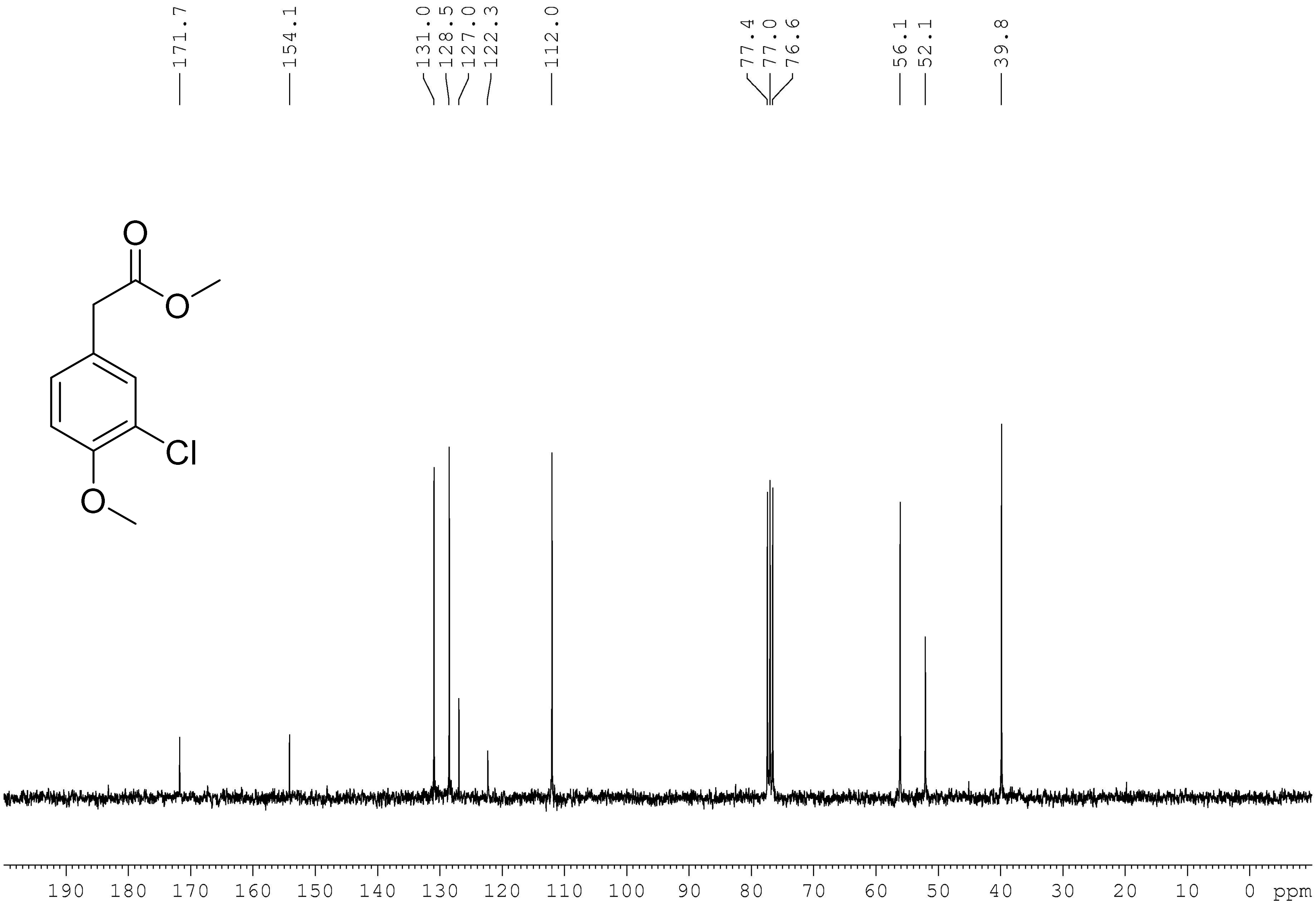

$^{13}C\{^{1}H\}$ NMR of compound **2aE** (75 MHz, $CDCl_3$)

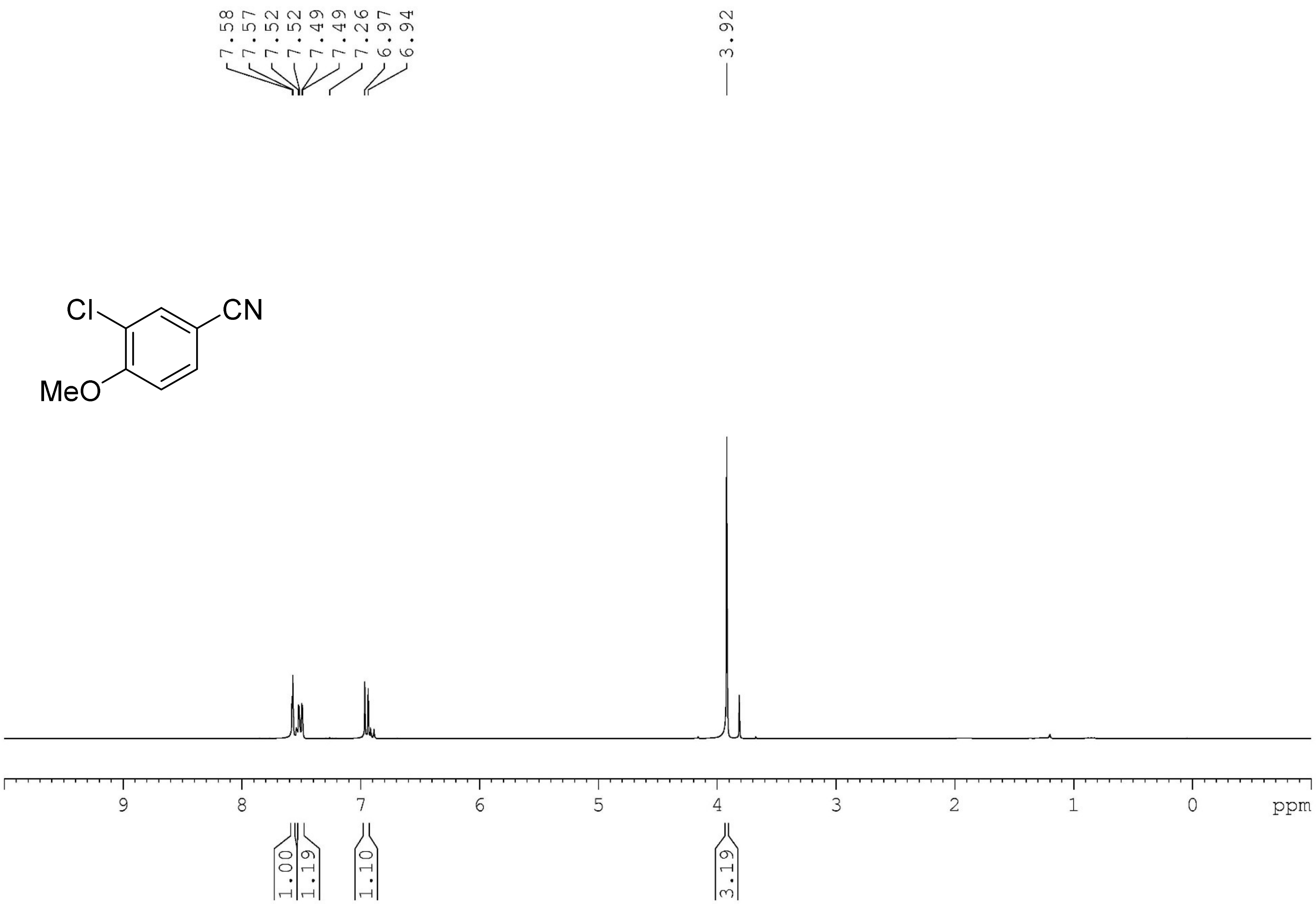

^{1}H NMR of compound **2aN** (300 MHz, $CDCl_3$)

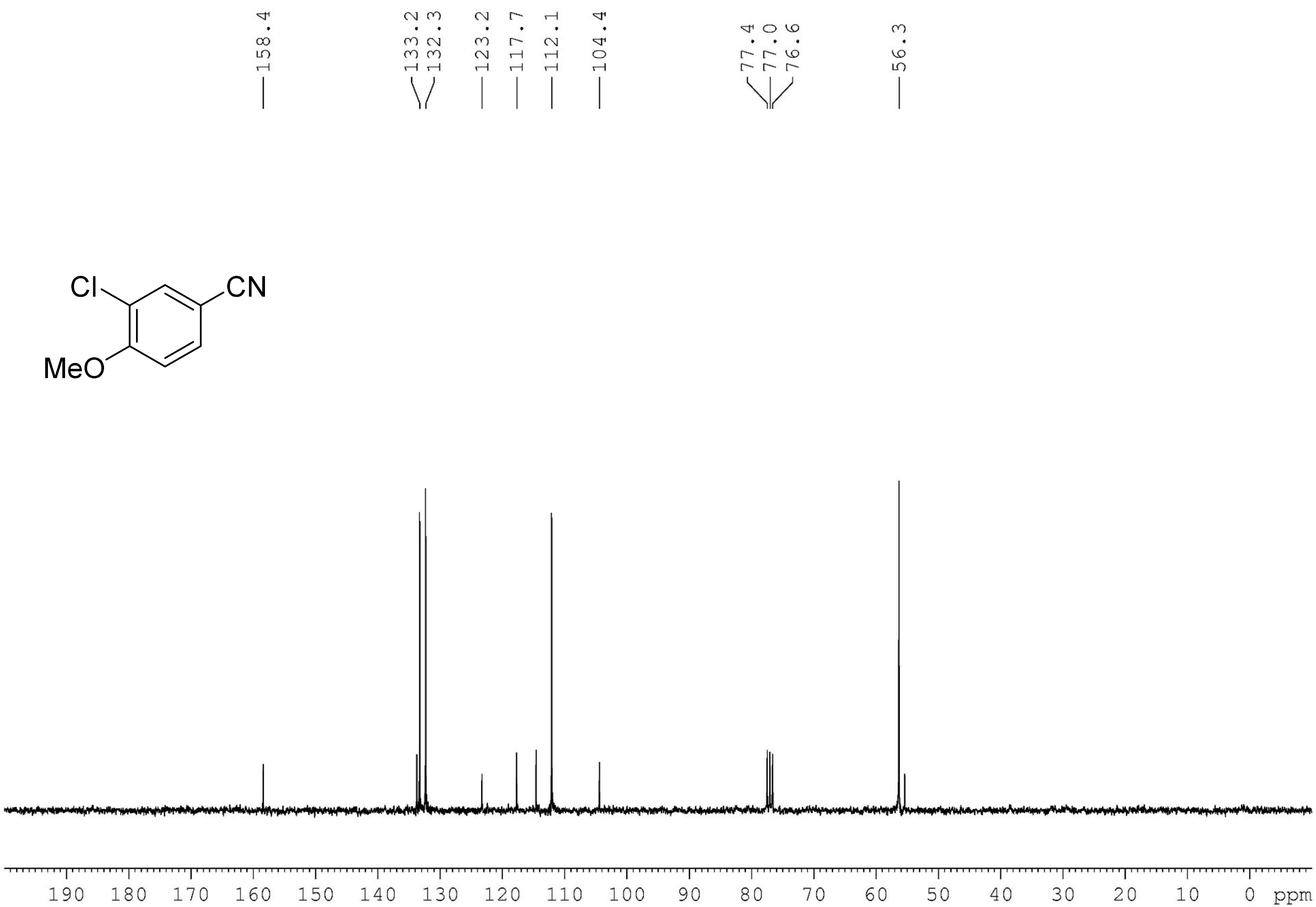

$^{13}C\{^{1}H\}$ NMR of compound **2aN** (75 MHz, $CDCl_3$)

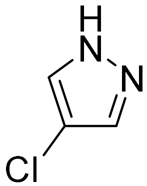

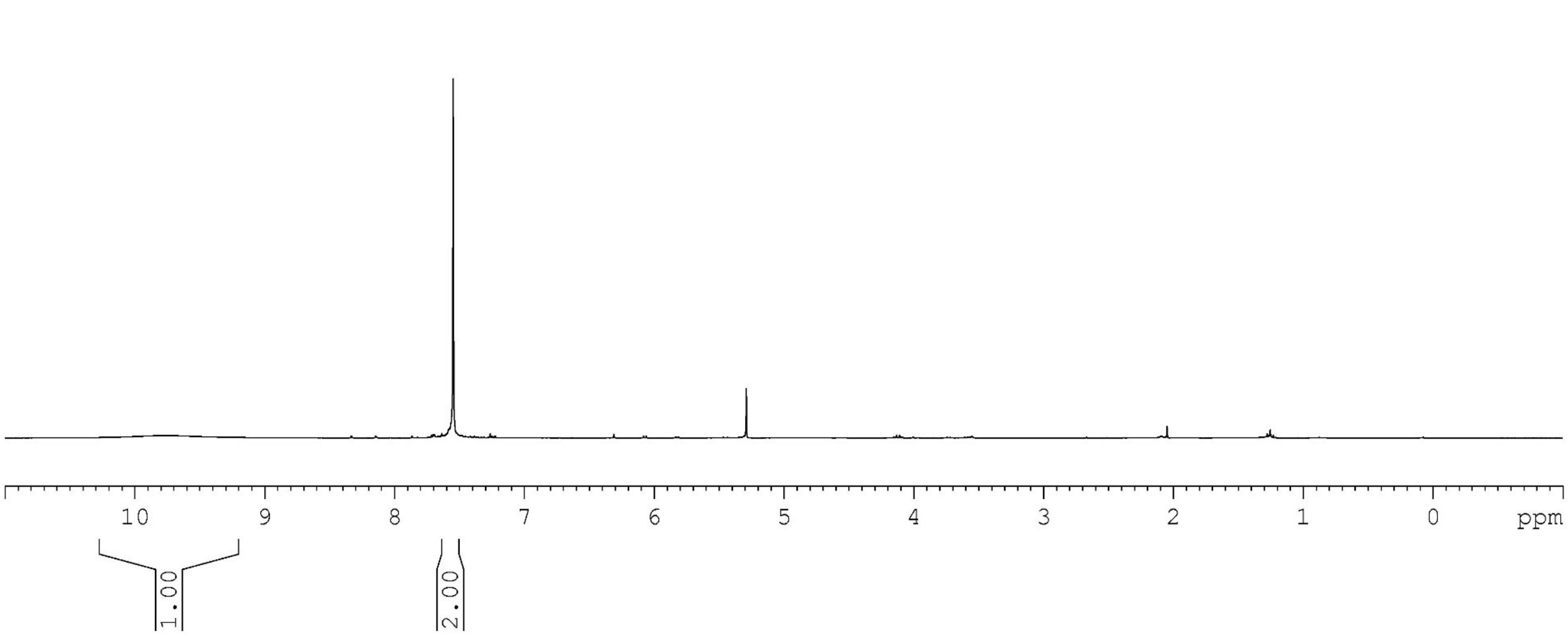

^1H NMR of compound **2pr** (300 MHz, $CDCl_3$)

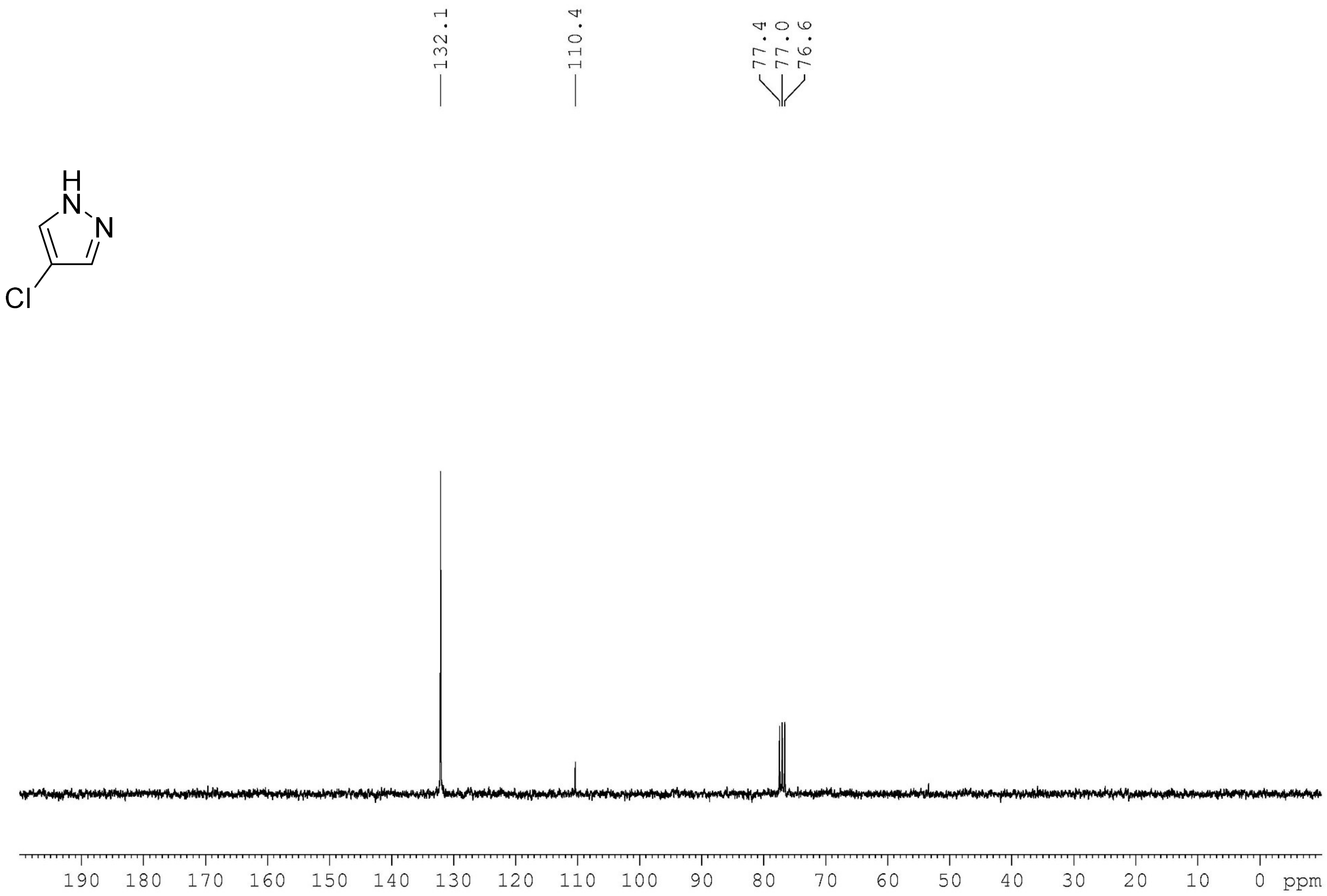

$^{13}C\{^{1}H\}$ NMR of compound **2pr** (75 MHz, $CDCl_3$)

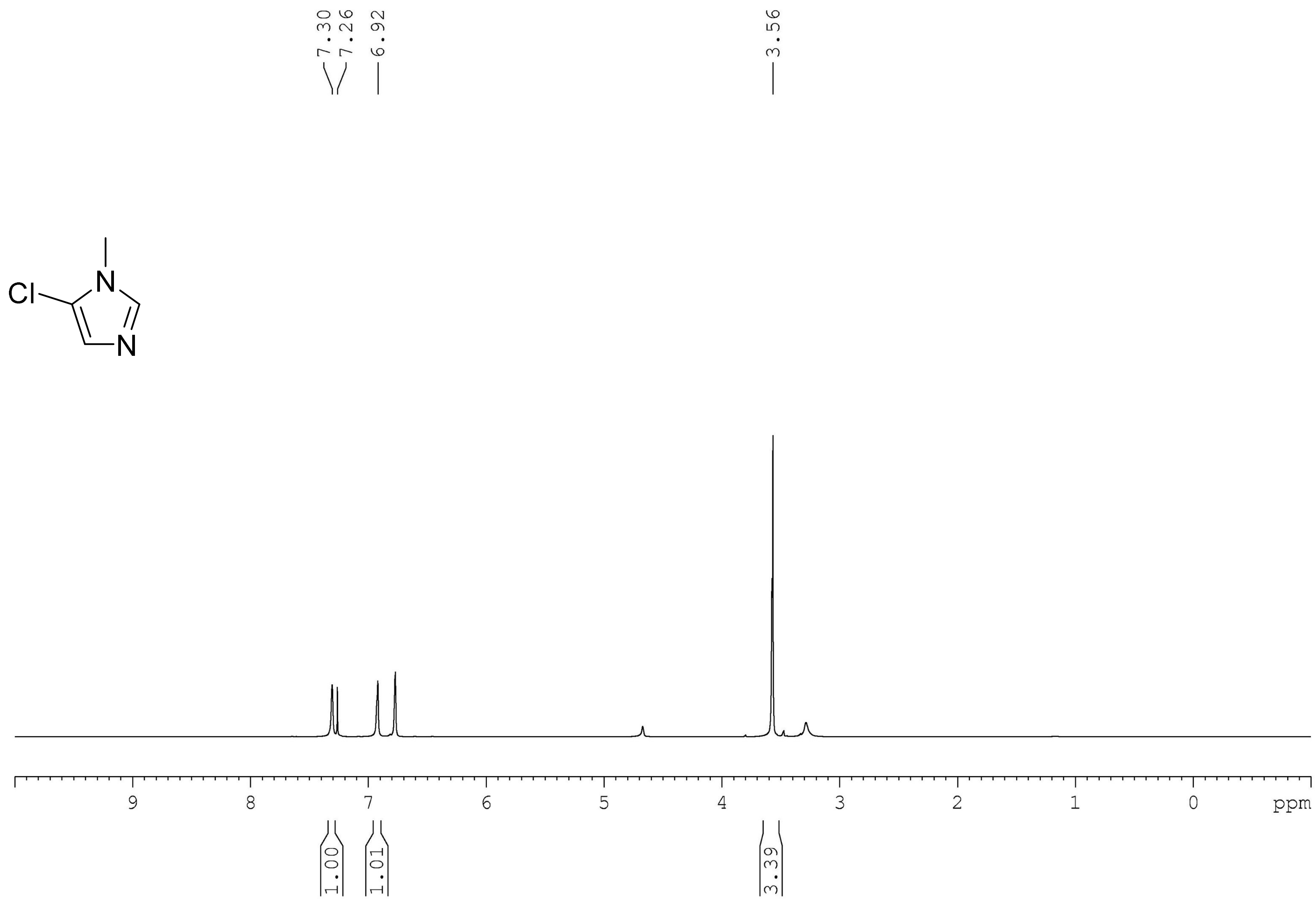

^{1}H NMR of compound **2im** (300 MHz, $CDCl_3$ + NaOH)

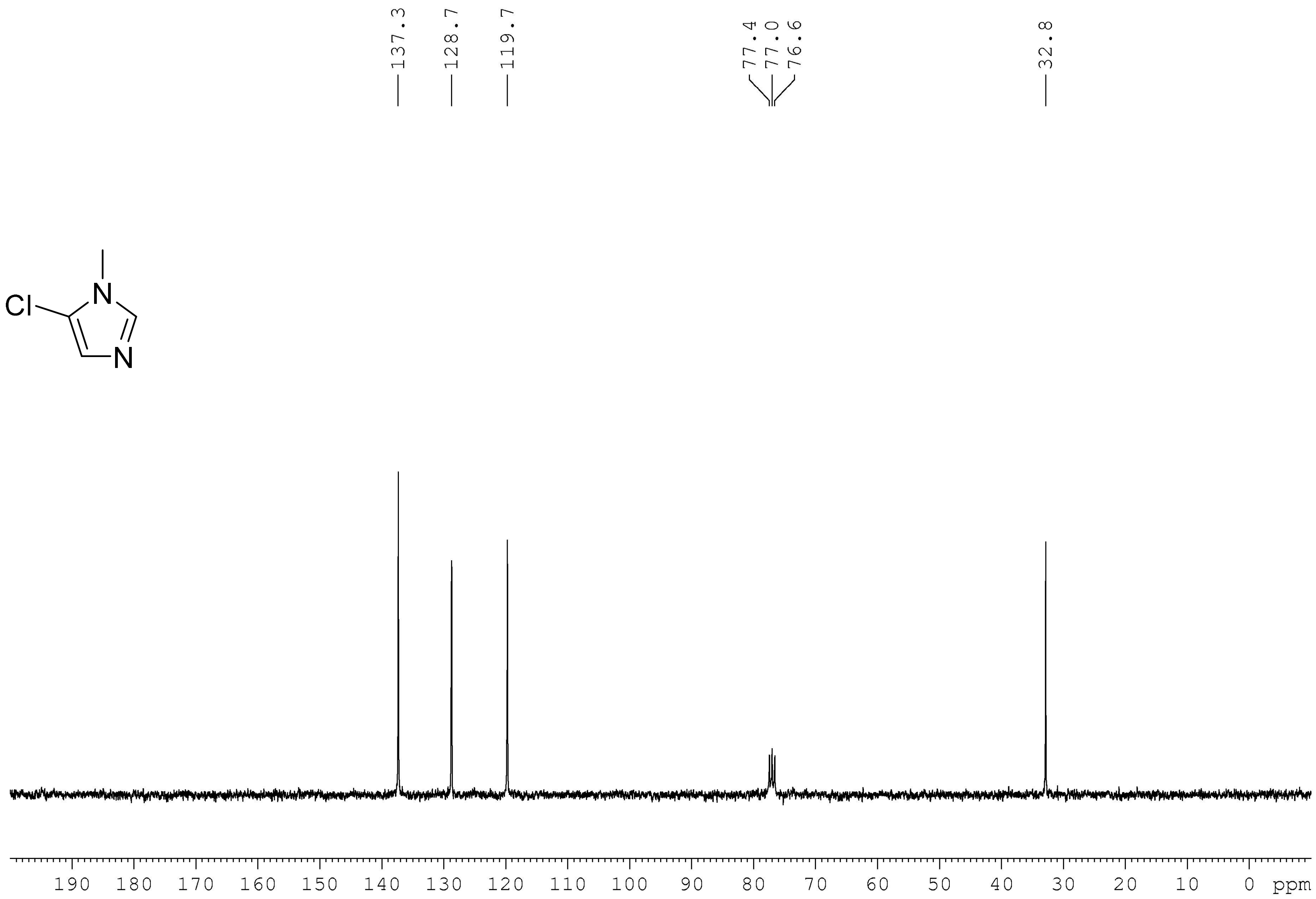

$^{13}C\{^{1}H\}$ NMR of compound **2im** (75 MHz, $CDCl_3$)

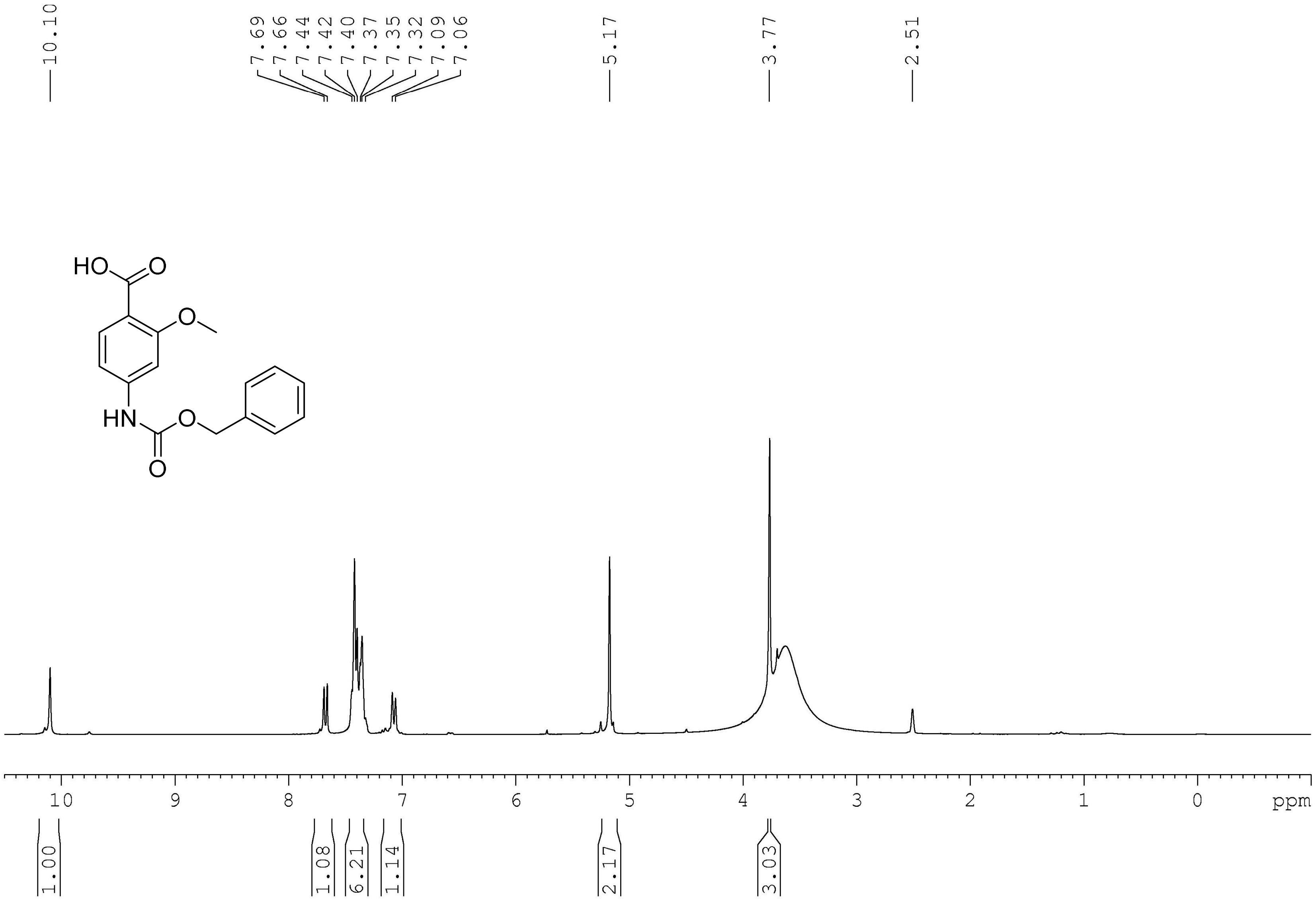

^{1}H NMR of compound **4** (300 MHz, d_6-DMSO)

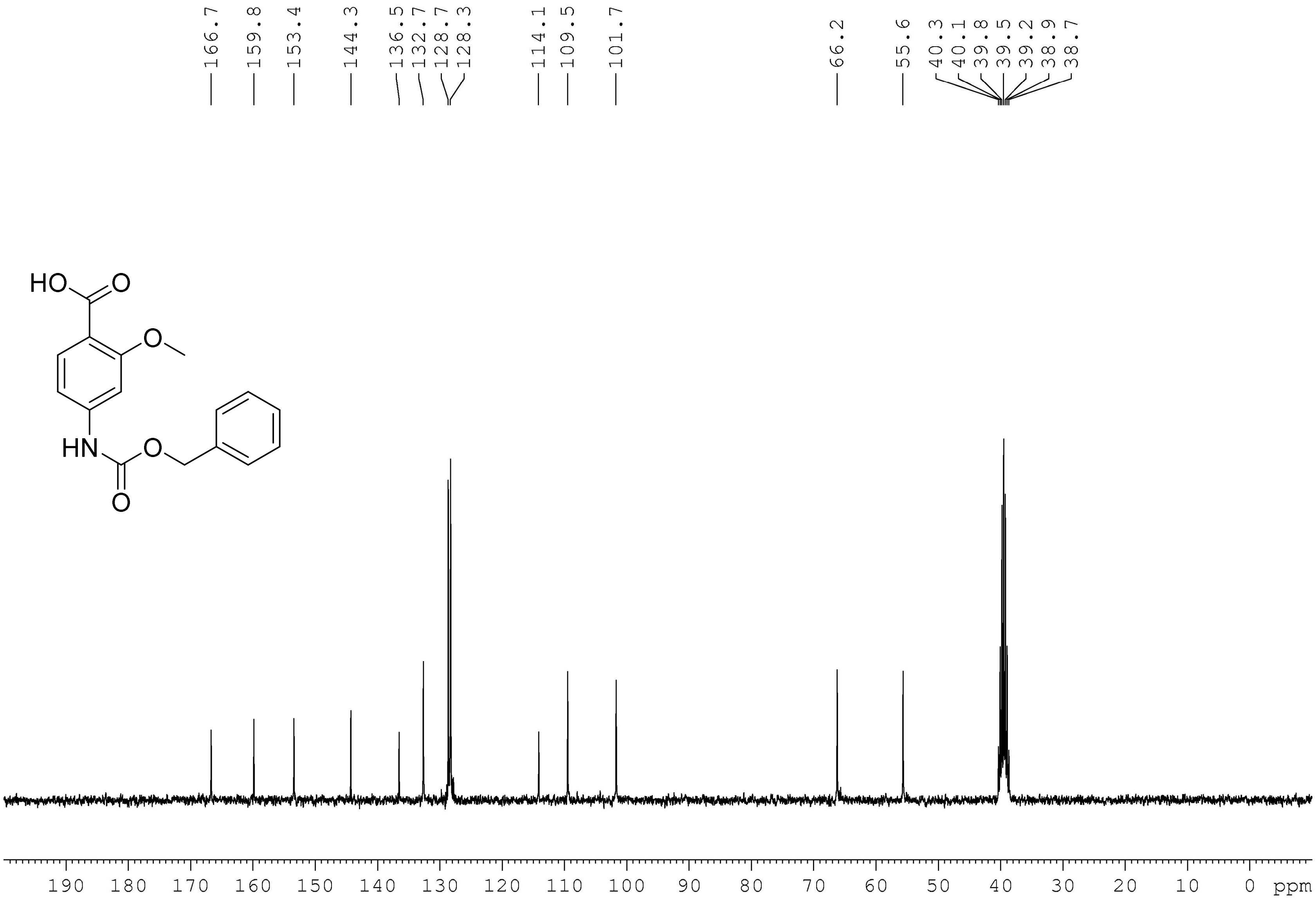

$^{13}C\{^{1}H\}$ NMR of compound **4** (75 MHz, d_6-DMSO)

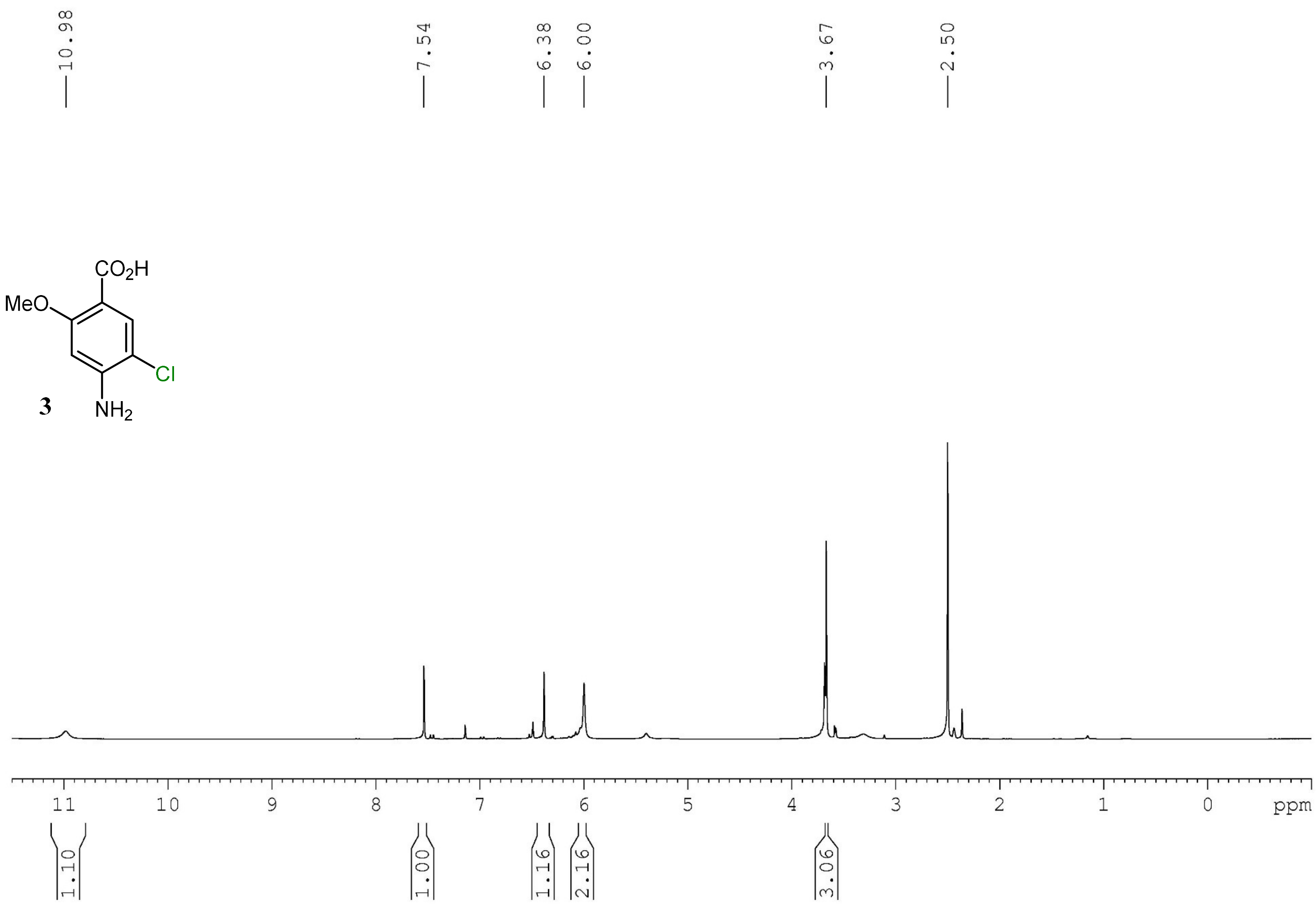

^{1}H NMR of compound **3** (300 MHz, d_6-DMSO)

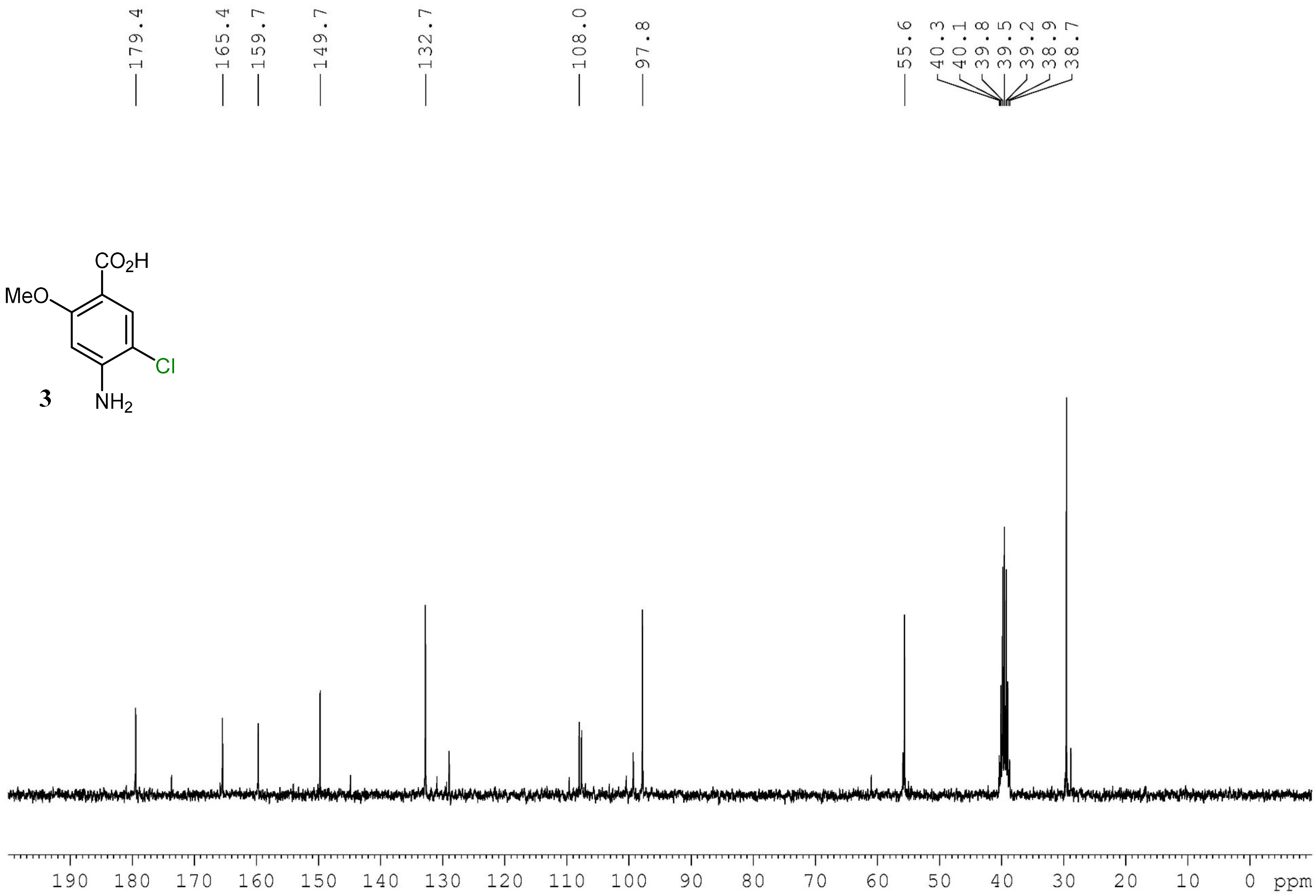

$^{13}C\{^{1}H\}$ NMR of compound **3** (75 MHz, d_6-DMSO)

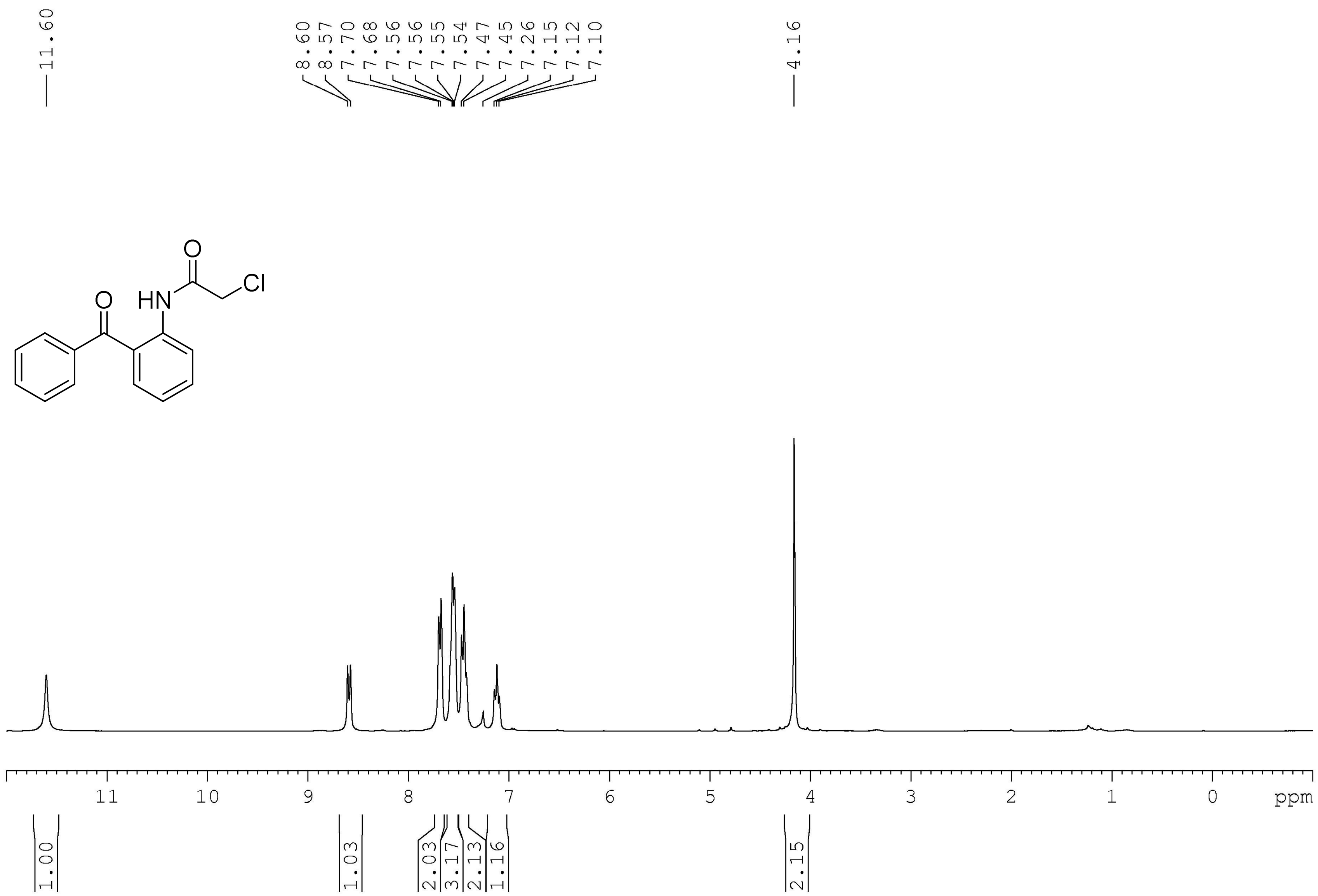

^{1}H NMR of compound **5** (300 MHz, $CDCl_3$)

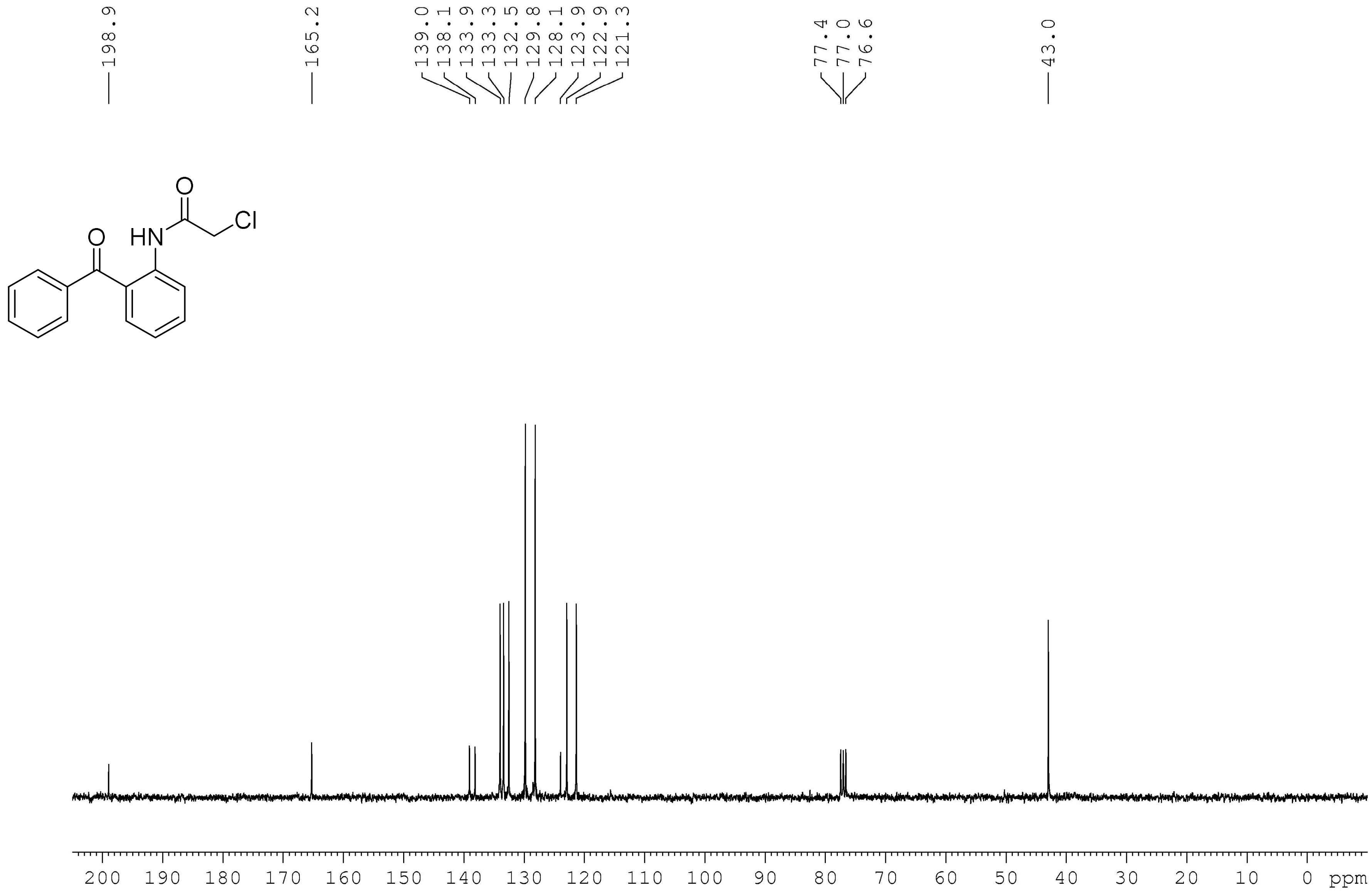

$^{13}C\{^{1}H\}$ NMR of compound **5** (75 MHz, $CDCl_3$)

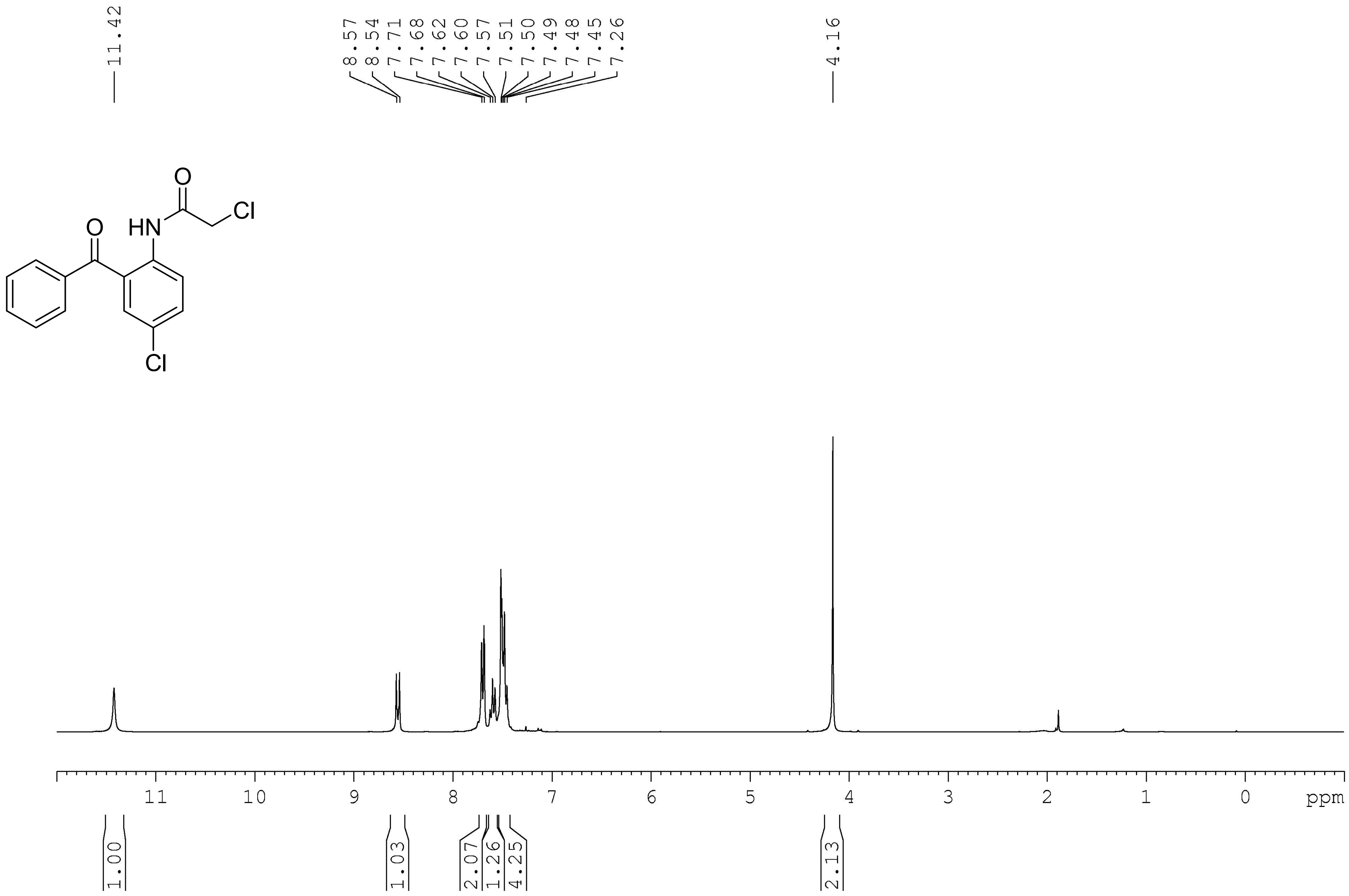

^{1}H NMR of compound **6** (300 MHz, $CDCl_3$)

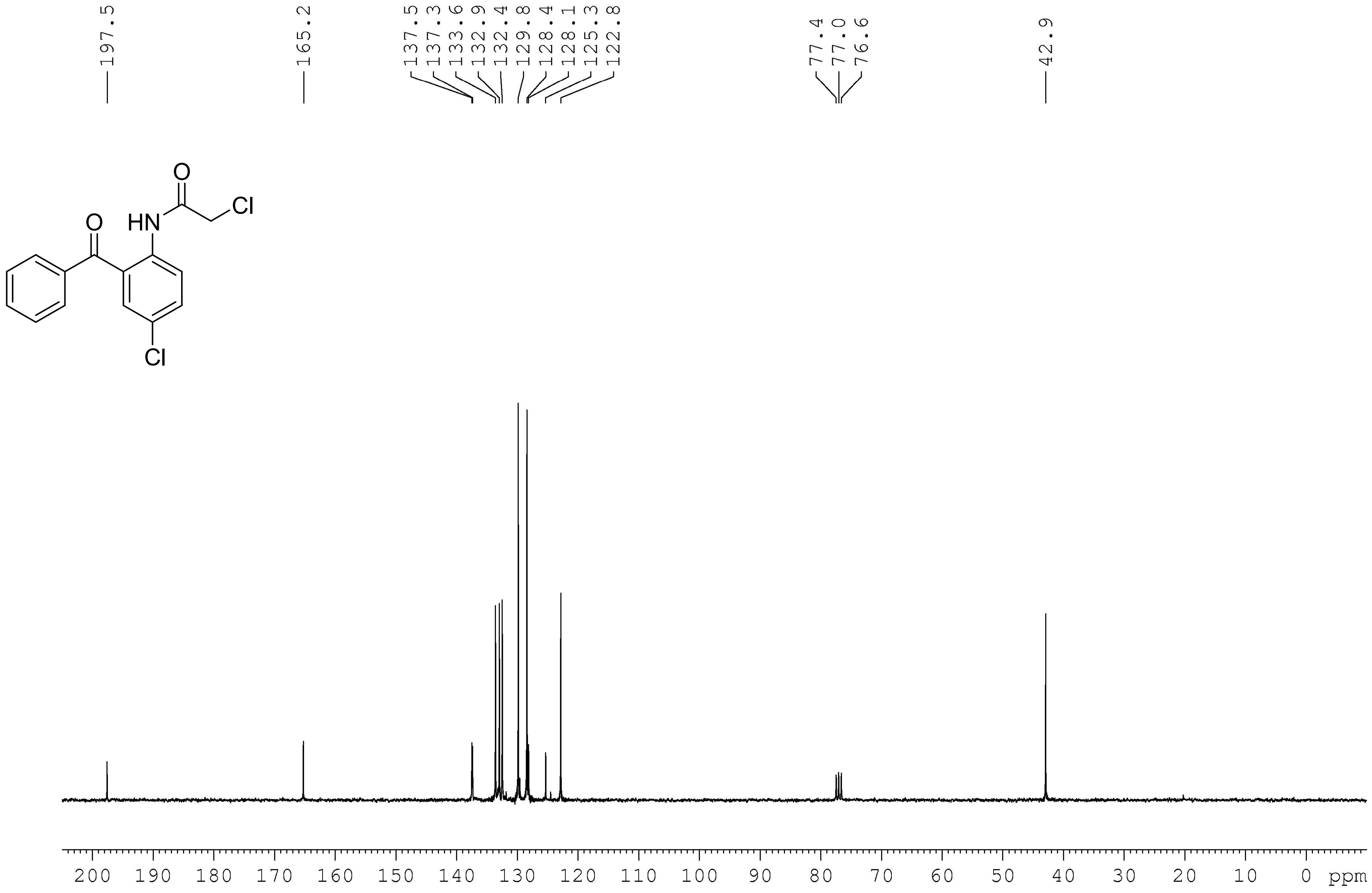

$^{13}C\{^1H\}$ NMR of compound **6** (75 MHz, $CDCl_3$)

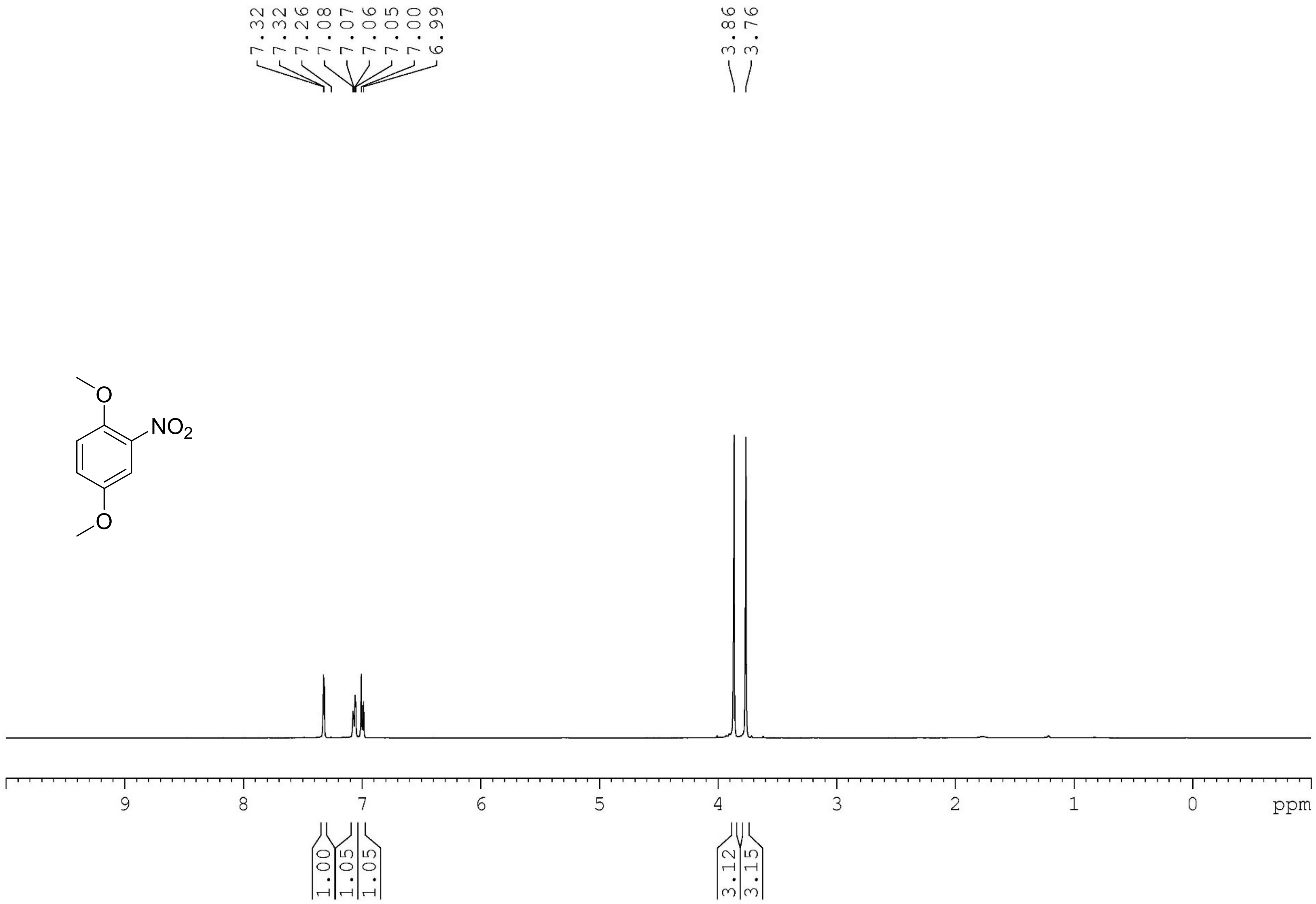

^{1}H NMR of compound **7** (500 MHz, $CDCl_3$)

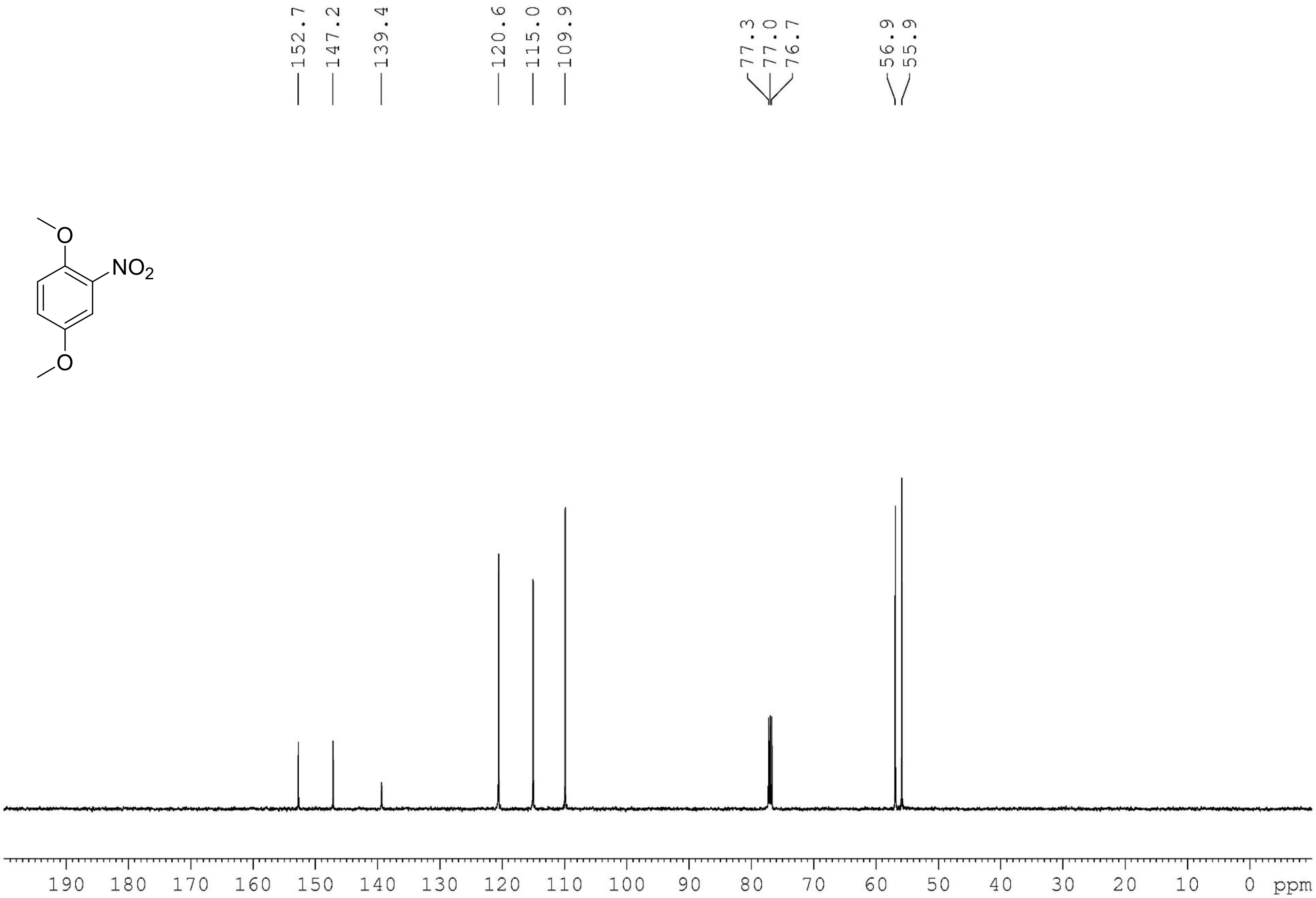

$^{13}C\{^{1}H\}$ NMR of compound **7** (126 MHz, $CDCl_3$)
