# Supplementary figures and images for: Nitrosonium Ion-Catalyzed Oxidative Chlorination of Arenes
Source: J Org Chem. 2026 Jan 5;91(2):892–903. doi: 10.1021/acs.joc.5c01914 (PMC12814551; doi:10.1021/acs.joc.5c01914)

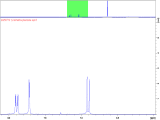

Supplement: Supplementary file 2 [file jo5c01914_si_002.zip › chlorination FID/1,2-dimethoxy-4-nitrobenzene/1/pdata/1/thumb.png]

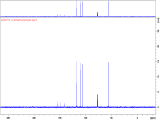

Supplement: Supplementary file 2 [file jo5c01914_si_002.zip › chlorination FID/1,2-dimethoxy-4-nitrobenzene/13/pdata/1/thumb.png]

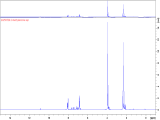

Supplement: Supplementary file 2 [file jo5c01914_si_002.zip › chlorination FID/1-methoxy-2-methyl-4-nitrobenzene/1/pdata/1/thumb.png]

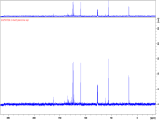

Supplement: Supplementary file 2 [file jo5c01914_si_002.zip › chlorination FID/1-methoxy-2-methyl-4-nitrobenzene/13/pdata/1/thumb.png]

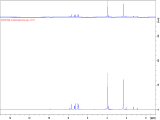

Supplement: Supplementary file 2 [file jo5c01914_si_002.zip › chlorination FID/1-methoxy-4-methyl-2-nitrobenzene/1/pdata/1/thumb.png]

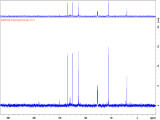

Supplement: Supplementary file 2 [file jo5c01914_si_002.zip › chlorination FID/1-methoxy-4-methyl-2-nitrobenzene/13/pdata/1/thumb.png]

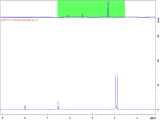

Supplement: Supplementary file 2 [file jo5c01914_si_002.zip › chlorination FID/2,4-dimethoxy-1-nitrobenzene/1/pdata/1/thumb.png]

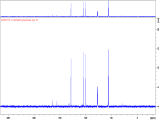

Supplement: Supplementary file 2 [file jo5c01914_si_002.zip › chlorination FID/2,4-dimethoxy-1-nitrobenzene/13/pdata/1/thumb.png]
